# Antidiabetic potential of a novel hydroxyphenyl-bi-benzopyran-hexol compound from *Cassia fistula* as an α-amylase inhibitor: Integrated *in silico* screening and *in vitro* validation using a nonlinear regression model

**DOI:** 10.1016/j.jaim.2026.101366

**Published:** 2026-07-24

**Authors:** Rajamanickam Baskar, Manoharan Harini, Sikkal Selvaraaju Selvapriya, Thangarasu Hemadevi, Arumugam Rajalakshmi, Kuppuswamy Kavitha, Rengarajulu Puvanakrishnan, Balasubramanian Ramesh

**Affiliations:** a Department of Gunapadam (Pharmacology), Government Siddha Medical College, The Tamil Nadu Dr. MGR Medical University, Chennai, Tamil Nadu, 600032, India; b PG and Research Department of Biotechnology, Sri Sankara Arts and Science College, University of Madras, Enathur, Kanchipuram, Tamil Nadu, 631561, India; c Government Siddha Medical College, The Tamil Nadu Dr. MGR Medical University, Chennai, Tamil Nadu, 600032, India; d Arignar Anna Government Hospital of Indian Medicine, Chennai, Tamil Nadu, 600106, India; e PG and Research Department of Microbiology, Sri Sankara Arts and Science College, University of Madras, Enathur, Kanchipuram, Tamil Nadu, 631561, India

**Keywords:** Diabetes mellitus, α-amylase inhibitor, Molecular dynamics simulation, *Cassia fistula*, α-amylase inhibition assay, Novel antidiabetic compounds

## Abstract

**Background:**

Diabetes mellitus is a critically important metabolic disease, causing persistent hyperglycemia. Blood glucose levels can be effectively maintained by inhibiting the activity of α-amylase. α-amylase inhibitors manage postprandial hyperglycemia by delaying carbohydrate digestion.

**Objectives:**

Identification of a potent α-amylase inhibitor from 915 bioactive compounds of Madhumukthi Kudineer Chooranum (MKC) by combining siddha knowledge with computational and experimental methods.

**Materials and methods:**

Molecular docking of bioactive compounds present in MKC with α-amylase was performed, and the top ten compounds that exhibited binding affinities better than the standard inhibitor acarbose were evaluated for Absorption, Distribution, Metabolism, Excretion, and Toxicity (ADMET) properties, followed by Molecular Dynamics Simulations (MDS). An *in vitro* enzyme inhibition assay was performed for the top lead for validation of the *in silico* findings.

**Results:**

Lead 2, Hydroxyphenyl-bi-benzopyran-hexol from *Cassia fistula* demonstrated the stable protein-ligand interaction with a binding energy of −69.70 ± 3.93 kJ/mol. The crude *Cassia fistula* stem bark extract containing Lead 2 showed 57% inhibition of α-amylase. The IC_50_ values were calculated using a nonlinear regression approach based on a 4-parameter logistic (4 PL) model, yielding a lower IC_50_ value of 237.25 μg/ml for the extract, compared to the IC_50_ value of 264.59 μg/ml for acarbose.

**Conclusion:**

By integrating traditional siddha knowledge with advanced computational screening and *in vitro* validation, this study proposed a novel Hydroxyphenyl-bi-benzopyran-hexol compound from *Cassia fistula* as a promising natural α-amylase inhibitor. However, these findings are to be validated using animal studies before clinical research leading to a novel drug.

## 1 Introduction

Type 2 diabetes mellitus (T2DM), a common public health concern, is characterized by chronic elevation of blood glucose resulting from insufficient insulin secretion and/or action. The International Diabetes Federation estimates that the number of adults living with diabetes in India will reach approximately 156.7 million by 2050 [1].

One of the therapeutic approaches to manage T2DM involves inhibiting digestive enzymes, specifically α-amylase, which is responsible for carbohydrate digestion [2,3]. By slowing the breakdown of starch into glucose, α-amylase inhibitors reduce postprandial hyperglycemia, thereby mitigating the complications associated with T2DM [4]. Inhibiting α-amylase is a promising strategy for managing type 2 diabetes and for controlling postprandial blood glucose levels. α-amylase inhibitors bind to the active-site residues Glu233 and Asp300, or bind to the pocket that surrounds the active site, preventing the breakdown of carbohydrates into glucose [5].

Current antidiabetic drugs can have various side effects depending on their class. Metformin is a widely prescribed oral antidiabetic drug for T2DM. While generally well-tolerated, metformin can cause critical side effects, like metformin-associated lactic acidosis [6], gastrointestinal adverse events [7], vitamin B12 deficiency [8], and rarely, hepatic dysfunction [9]. Sulfonylureas can lead to significant hypoglycemia and weight gain [10], and in some cases, cardiovascular risks are increased [11]. Similarly, meglitinide analogs are hindered by their frequent dosing requirements, high cost, risk of hypoglycemia, and peripheral edema [12], whereas α-glucosidase inhibitors are limited by their complex dosing schedules and gastrointestinal side effects [13].

Unlike other antidiabetics, the use of α-amylase inhibitors can help in weight management by reducing the overall calorific intake from carbohydrates [14]. Currently available α-amylase inhibitors, such as acarbose, miglitol, and voglibose, primarily lead to liver damage, heart failure, weight gain, hyperglycemia, abdominal pain, and flatulence [15]. Genetic polymorphisms in patients are strongly linked to variations in responses and adverse effects of antidiabetic drugs [16]. Addressing these side effects requires safer and more effective antidiabetic therapies, including natural or plant-based alternatives. Natural compounds, particularly those derived from medicinal plants, have gained attention for their potential to serve as α-amylase inhibitors due to their ability to modulate glucose metabolism with fewer adverse effects than synthetic drugs [2].

Recent advancements in computational biology have facilitated the use of *in silico* methods to identify and evaluate potential bioactive compounds. These approaches have enabled researchers to screen extensive libraries of natural products and predict their binding affinities and interactions with target enzymes [17]. By utilizing molecular docking in combination with molecular dynamics simulations, *in silico* studies have the potential to streamline the discovery of novel α-amylase inhibitors, ultimately leading to new therapeutic options for diabetes management [18].

Siddha medicine, an ancient system of South India, offers several advantages in managing diabetes through its holistic and natural approaches. Siddha formulations primarily consist of herbal ingredients, minerals, and animal products, which are known for their efficacy and minimal side effects compared to synthetic pharmaceuticals. It focuses on preventing diabetes-related complications by addressing overall health and promoting lifestyle changes that support metabolic balance [19].

Madhumukthi Kudineer Chooranum (MKC) is a traditional herbal formulation used in Siddha medicine, specifically formulated for the management of diabetes. This polyherbal formulation is known for its potent antidiabetic properties and has been widely used as part of the holistic approach in the treatment of diabetes mellitus, particularly T2DM [20].

This study investigates the natural compounds in the herbal formulation MKC to identify bioactive compounds that function as α-amylase inhibitors. The objectives of this study were virtual screening of bioactive compounds from MKC for their α-amylase inhibition, evaluation of Absorption, Distribution, Metabolism, Excretion, and Toxicity (ADMET) properties for the top lead compounds, stability and interaction analysis of selected lead compounds with α-amylase through molecular dynamics simulations, and validation of the *in silico* results by *in vitro* α-amylase inhibition assays.

## 2 Materials and methods

### 2.1 Identification and collection of bioactive compounds from MKC

The major plants used in the preparation of MKC are *Salacia oblonga, Azadirachta indica, Aegle marmelos, Tinospora cordifolia,* and *Cassia fistula* [20]. A screening database was created using the 915 bioactive compounds identified in these plants, which were obtained from the PubChem database [21].

### 2.2 Target pre-processing

The structure of human α-amylase (PDB ID: 4W93) with a resolution of 1.35 Å, obtained by X-ray diffraction, was retrieved from Research Collaboratory for Structural Bioinformatics - Protein Data Bank (RCSB - PDB) [22]. The enzyme target was prepared by removing the co-crystallized inhibitor and water molecules from the structure, and Swiss-PDB Viewer v4.1.0 [23] was used to fix the incomplete residues.

### 2.3 Molecular docking

Molecular docking was carried out to screen the ligands against the target enzyme using Vina-GPU v.2.0 [24] by setting a grid of 30 Å in all dimensions around TRP58 with the coordinates x = −2.635, y = 11.433, and z = −23.626, which was recognized as an effective binding pocket by the Computed Atlas of Surface Topography of proteins (CASTp 3.0) tool [25]. BIOVIA Discovery Studio Visualizer v21.1.0.20298 [26] was used to analyze and render the best binding conformations.

### 2.4 Evaluation of ADMET properties

ADMET properties were predicted for all the natural compounds, along with the positive control acarbose, using the SwissADME server [27]. The probability of causing Drug-Induced Liver Injury, acute oral toxicity, AMES mutagenesis, and carcinogenicity was calculated by the ADMETlab 3.0 tool [28].

### 2.5 MD simulations

MD simulations were carried out using the GROMACS 2022.4 software. Pdb2gmx program was used to generate the topology file of the target protein, and the CGenFF server built the topology parameters for the top three lead compounds and the positive control. MD simulations were carried out for five systems: one apoenzyme and four complexes, *viz.,* 4W93 – Lead1, 4W93 – Lead2, 4W93 – Lead3, and 4W93 – positive control. The Solvent box was created for the apo-protein and protein-ligand complexes with TIP3P water molecules and stabilized by three Sodium ions to ensure electroneutrality. NVT and NPT equilibrations were performed to attain 300 K temperature and 1 bar pressure, respectively, followed by the convergence of the system through energy minimization using the Steepest Descent algorithm. MD simulations of 500 ns were performed by the gmx mdrun program for all the systems. The tools gmx gyrate, gmx hbond, gmx rms, and gmx rmsf were used to calculate the Radius of gyration (Rg), the number of hydrogen bonds formed, Root Mean Square Deviation (RMSD), and Root Mean Square Fluctuation (RMSF) of the systems, respectively [29].

### 2.6 Calculation of binding energy using the thermodynamic parameters

Electrostatic, polar solvation, nonpolar solvation, and Van der Waals energies of the systems were calculated by Molecular Mechanics – Poisson Boltzmann Surface Area solvation (MM-PBSA) using the gmx_MMPBSA tool [30] to determine the total binding energies for each ligand.

### 2.7 Principal component analysis (PCA)

PCA was carried out for all five systems to investigate large-scale collective motions of the protein during MD simulation and to achieve dimensionality reduction. The covariance matrix of Cα atomic positional fluctuations was generated using the gmx covar in GROMACS v2022.04. Eigenvalues and eigenvectors were then computed to identify the dominant motion modes. The first two principal components, representing the highest variance, were projected along these components using gmx anaeig. A Free Energy landscape (FEL) based on PC1 and PC2 was constructed using the tool gmx sham [31].

### *2.8* *In vitro* α-amylase inhibition assay

α-amylase of concentration 0.5 U/ml was prepared using phosphate buffer (pH 6.9). *Cassia fistula* stem bark (CFSB) was collected and authenticated by Siddha Central Research Institute, Central Council for Research in Siddha, Ministry of Ayush, Government of India, Anna Government Hospital Campus, Arumbakkam, Chennai – 600106, with certificate number: PCOG002-ACF (Fig. S1). Double-distilled water extracts of CFSB and standard acarbose were prepared in different concentrations of 100, 200, 300, 400, and 500 μg/ml. From these solutions, 600 μL of each test sample was added to 30 μL of the α-amylase enzyme solution and incubated for 15 min at 37 °C. About 370 μl of substrate, 2-Chloro-4-Nitrophenyl-α-Maltotrioside (0.5 mg/ml) was added and incubated at 37 °C for 10 min. The absorbance of the sample was measured at 405 nm. The percentage inhibition was calculated using the following formula [32].

$$\text{Percentage}\;\text{inhibition}=\frac{{\text{Absorbance}}_{\text{control}}‐{\text{Absorbance}}_{\text{Test}}}{{\text{Absorbance}}_{\text{control}}}\times 100$$

The IC_50_ values were calculated using a nonlinear regression approach with a 4-parameter logistic (4 PL) model for fitting dose–response [33,34]. Python scipy.optimize.curve_fit function was used to fit the 4 PL model to estimate the best-fit parameters (A, B, C, and D).

The 4-parameter logistic equation is:

$$Y=\frac{A\;–\;D}{1+{\left(X/C\right)}^{B}}+D$$

Where **X** = inhibitor concentration, **Y** = % inhibition, **A** = minimum response (bottom asymptote), **D** = maximum response (top asymptote), **B** = Hill slope (curve steepness), and **C**=**IC_50_** (the concentration at which inhibition is 50%).

### 2.9 Statistical analysis

Experimental values were mentioned as mean ± SEM. Statistical analyses were performed using Jamovi v2.6.2.0. [35]. Descriptive statistics, independent t-tests, one-way ANOVA with Tukey's post hoc tests, Chi-square tests, and Pearson correlations were applied with a 95 % confidence level (α = 0.05) and p-values between p ≤ 0.05 and p ≤ 0.0001.

## 3 Results

### 3.1 Screening of bioactive compounds by molecular docking

Molecular docking of the 915 bioactive compounds (Table S1) with α-amylase was performed, and 123 compounds exhibited negative binding energies compared to the positive control acarbose. The binding affinities of the top ten compounds, along with the positive control and the interacting amino acids of the target, are shown in Table 1. The receptor-ligand interactions of the top three lead compounds and the positive control acarbose are shown in Fig. 1 as 2D diagrams. The results showed that all the top 10 leads exhibited stronger binding affinities to α-amylase compared to acarbose (−5.5 kcal/mol). Most of the lead compounds involved in binding with GLN302, GLY304, ASP317, ALA310, ARG267, and PHE348 are crucial for the catalytic activity of α-amylase. Important bonds observed between the receptor and lead compounds were hydrogen bonds, hydrophobic interactions, pi-stacking, and pi-alkyl interactions.

**Table 1:** Interaction analysis and Binding affinities of the lead compounds and positive control with the α-amylase enzyme.

Compound	Structure	Binding affinity (kcal/mol)	Binding Interactions			
			Van der	H Bond	C – H bond	Pi-Bond
			Waals			
			Interaction			
Lead 1 – (2S,4AR,6aR,7R,10R,10aS,10bS)-2-(furan-3-yl)-7-hydroxy-6a,10b-dimethyl-4a,5,6,6a,7,10,10a,10b-octahydro-1H-10,7-(epoxymethano)benzo[f]isochromene-4,12(2H)-dione		−7.7	GLN A:302	ARG A:267	TRP A:316	ALA A:310
			GLY A:304	THR A:314		
			ASP A:317			
			ARG A:346			
			PHE A:348			
Lead 2 – (2S,2′S,3S,3′S,4S)-3,3′,4,4′-Tetrahydro-2beta-(3,4-dihydroxyphenyl)-2′beta-(4-hydroxyphenyl)-4α,8′-bi[2H-1-benzopyran]-3beta,3′beta,5,5′,7,7′-hexol		−7.4	ARG A:267	GLN A:302		ALA A:310
			TRP A:269	ASP A:317		ASP A:317
			GLY A:304			ARG A:346
			TRP A:316			
			PHE A:348			
Lead 3-(2R,3R)-8-[(2S,4R)-7-hydroxy-2-(4-hydroxyphenyl)-3,4-dihydro-2H-chromen-4-yl]-2-(4-hydroxyphenyl)-3,4-dihydro-2H-chromene-3,5,7-triol		−7.2		GLN A:302	GLY A:351	ARG A:267
				GLY A:304		ALA A:310
						ASP A:317
						PHE A:348
Lead 4 - 12-methyl-3,7-dioxopodocarpa-8,11,13-triene-13-carboxylic acid		−7.2		GLN A:302		ALA A:310
				THR A:314		PHE A:348
Lead 5 - [(1R,2R,5R,6R,11R,12S,13R,16R,17R,19S,20R)-6-(furan-3-yl)-12,17-dihydroxy-1,5,11,16-tetramethyl-8-oxo-7,14-dioxapentacyclo[11.6.1.02,11.05,10.016,20]icos-9-en-19-yl] 2,3-dimethylbut-2-enoate		−7	ALA A:310			PHE A:348
			THR A:314			
Lead 6 – (1aR,4S,4aS,7R,7aR,7bS)-1,1,4,7-tetramethyl-2,3,4a,5,6,7,7a,7b-octahydro-1aH-cyclopropa[e]azulen-4-ol		−6.9		GLN A:302		ALA A:310
						TRP A:316
Lead 7 - (5R,9R,10R,13S,14S,17S)-17-[(1R)-1-[(2R,3S,4S)-3,4-dihydroxy-5,5-dimethyloxolan-2-yl]ethyl]-4,4,10,13,14-pentamethyl-1,2,5,6,9,11,12,15,16,17-decahydrocyclopenta[a]phenanthren-3-one		−6.9	LEU A:237			
			LYS A:257			
			ALA A:260			
			LYS A:261			
			THR A:264			
			GLY A:308			
			SER A:311			
Lead 8 - 12-Hydroxy-13-methylpodocarpa-8,11,13-triene-3,7-dione		−6.9		ARG A:267		PHE A:348
Lead 9 - (4aS,10aR)-1,1,4a-trimethyl-7-propan-2-yl-3,4,10,10a-tetrahydrophenanthrene-2,9-dione		−6.9				ALA A:310
						PHE A:348
Lead 10 - (2S,2′S,3S,3′S,4S)-3,3′,4,4′-Tetrahydro-2beta,2′beta-bis(4-hydroxyphenyl)-4alpha,8′-bi[2H-1-benzopyran]-3beta,3′beta,5,5′,7,7′-hexol		−6.8	PHE A:348	GLY A:309	GLY A:304	ALA A:310
				ASP A:317		
Positive control – Acarbose		−5.5		GLY A:304	GLY A:309	
				ALA A:310	GLY A:351	
				ASP A:317

**Fig. 1:**
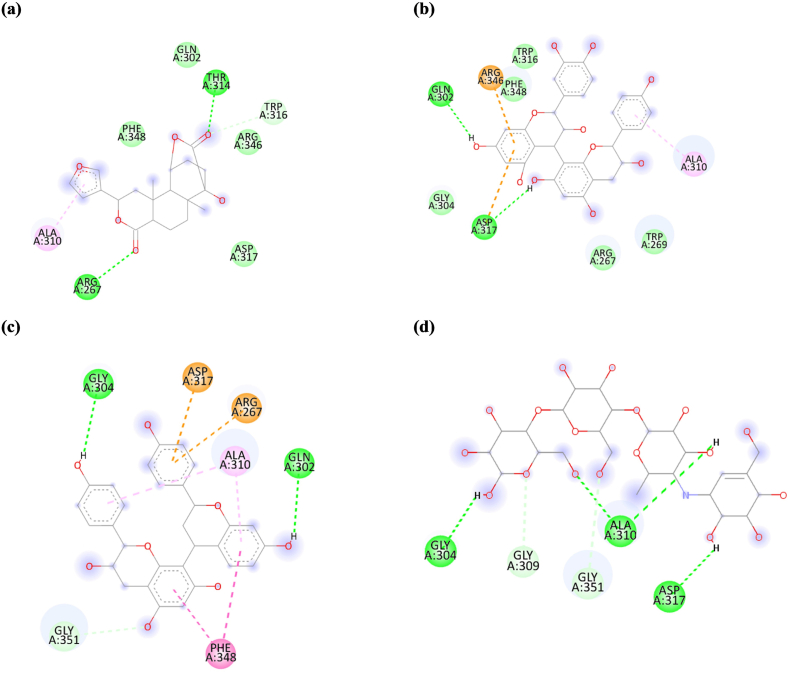
Amino acid interactions (Green – Hydrogen bond, Blue – Carbon–Hydrogen Bond, Tangerine – Pi-Anion/Cation, Magenta – Pi-Pi interaction, and Pink – Pi-Alkyl interaction) of lead compounds with α-amylase and (a) lead1, (b) lead2, (c) lead3, and (d) positive control (Acarbose). (For interpretation of the references to color in this figure legend, the reader is referred to the Web version of this article.)

### 3.2 ADMET evaluation

For all the natural compounds and positive control, pharmacokinetic properties, druglikeness, medicinal chemistry properties, toxicity, and carcinogenicity were evaluated (Tables S2 and S3). Among them, the ADMET properties of the top ten leads based on binding affinity were presented in Table 2. The drug-like properties of natural compounds, based on Lipinski's rule, were analysed by comparing them with the positive control, acarbose (Fig. S2). The top three lead compounds showed better drug-likeness and toxicity properties than acarbose. Lead1, Lead2, and Lead3 demonstrated high compliance with druglikeness rules [[36], [37], [38], [39], [40]]. Lead 1 complied with all five druglikeness rules, while acarbose violated all rules. The synthetic accessibility scores for Lead1, Lead2, and Lead3 (5.37, 5.28, and 4.99, respectively) indicated that these compounds are easier to synthesize than acarbose, which showed a higher score of 7.34. Drug-induced liver injury probability scores for the top leads, more particularly Lead 2 (0.151) and Lead 3 (0.019), were significantly lower than that of acarbose (0.689), signifying the reduced hepatotoxicity of these leads. On the other hand, Lead 1 exhibited an acute oral toxicity value of 0.228 better than the very high value of acarbose (0.727), indicating a safer toxicity profile. Lead 1 showed high GI absorption, while the positive control, acarbose, showed low GI absorption, suggesting better oral bioavailability. Even though Leads 2 and 3 exhibited low GI absorption, their overall druglikeness, safety profiles, and favourable pharmacokinetic properties can offset this. Hence, the top three leads were selected for further molecular dynamics studies along with the positive control acarbose.

**Table 2:** ADMET properties of the top 10 leads and the positive control.

Compound ID	Pharmacokinetics					Druglikeness					Medicinal chemistry			Toxicity and carcinogenesis^c^			
	GI absorption	BBB permeant	P-gp substrate	Cytochrome P450 inhibitor	Skin permeation log K_p_ (cm/s)	Lipinski	Ghose	Veber	Egan	Muegge	PAINS #alerts	Brenk #alerts	Synthetic Accessibility Score^b^	Drug Induced Liver Injury	Ames Muta genesis	Acute oral toxicity	Carcinogenicity
Lead1	High	No	Yes	No	−6.95	Yes	Yes	Yes	Yes	Yes	0	2	5.37	0.927	0.295	0.228	0.76
Lead2	Low	No	No	No except CYP3A4	−7.8	No (3)^a^	No (2)^a^	No (1)^a^	No (1)^a^	No (3)^a^	1	1	5.28	0.151	0.387	0.658	0.123
Lead3	Low	No	No	No except CYP3A4	−6.31	No (2)^a^	No (2)^a^	Yes	No (1)^a^	No (1)^a^	0	0	4.99	0.019	0.235	0.783	0.394
Lead4	High	Yes	Yes	No except CYP2C19 and CYP3A4	−6.17	Yes	Yes	Yes	Yes	yes	0	0	3.34	0.782	0.392	0.352	0.397
Lead5	High	No	Yes	No	−6.57	No (1)^a^	No (3)^a^	Yes	Yes	yes	0	2	6.86	0.97	0.395	0.678	0.229
Lead6	High	Yes	No	No except CYP2C19	−5	Yes	Yes	Yes	Yes	No (1)^a^	0	0	3.58	0.185	0.301	0.091	0.817
Lead7	High	No	Yes	No	−5.31	No (1)^a^	No (3)^a^	Yes	Yes	No (1)^a^	0	1	6.35	0.149	0.112	0.258	0.202
Lead8	High	Yes	Yes	No except CYP2C19 and CYP3A4	−5.91	Yes	Yes	Yes	Yes	yes	0	0	3.15	0.252	0.486	0.407	0.518
Lead9	High	Yes	Yes	No except CYP2C19 and CYP3A4	−5.19	Yes	Yes	Yes	Yes	yes	0	0	3.33	0.425	0.318	0.42	0.481
Lead10	Low	No	No	No except CYP3A4	−7.45	No (2)^a^	No (2)^a^	No (1)^a^	No (1)^a^	No (2)	0	0	5.19	0.085	0.342	0.657	0.16
Positive - Acarbose	Low	No	Yes	No	−16.29	No (3)^a^	No (4)^a^	No (1)^a^	No (1)^a^	No (5)^a^	0	1	7.34	0.689	0.16	0.727	0.038

a Number of violations based on the respective drug-likeness rules.

b Synthetic accessibility score ranges from 1 to 10 (1 indicates easy synthesis and 10 denotes difficulty), and.

c The probability values of Toxicity and carcinogenesis from 0 to 1 (0 represents less probability and 1 signifies high probability).

### 3.3 MD simulations

MD simulations were performed for 500 ns for five systems containing docked complexes of α-amylase and leads, *viz.,* 4W93-Lead1, 4W93-Lead2, 4W93-Lead3, 4W93-Positive, and unliganded 4W93. The RMSD throughout the trajectory of the unliganded protein was 0.2676 ± 0.04 nm, and for the complexes 4W93-Lead1, 4W93-Lead2, 4W93-Lead3, and 4W93-Positive, the RMSD values were 0.3516 ± 0.05, 0.2254 ± 0.02, 0.2523 ± 0.02, and 0.2577 ± 0.04 nm, respectively. All complexes exhibited lower RMSD values, indicating enhanced structural stability upon ligand binding. 4W93-Lead2 complex showed the lowest RMSD at 0.2254 ± 0.02 nm, indicating that Lead2 formed a very stable complex with the enzyme, which correlated with effective inhibition. RMSF of the complexes were 0.1492 ± 0.14, 0.1333 ± 0.08, 0.1261 ± 0.09, and 0.1307 ± 0.11 nm for 4W93-Lead1, 4W93-Lead2, 4W93-Lead3, and 4W93-Positive, respectively, and for the unliganded protein it was 0.129 ± 0.09 nm. Lower RMSF values in the ligand-bound forms suggested reduced flexibility and increased rigidity, particularly around the active site, which helped maintain the optimal orientation of key residues for inhibition. The radius of gyration value of the unliganded protein was 2.387 ± 0.012 nm, and 4W93-Lead1, 4W93-Lead2, 4W93-Lead3, and 4W93-Positive were 2.388 ± 0.019, 2.378 ± 0.011, 2.373 ± 0.011, and 2.385 ± 0.012 nm, respectively. The decrease in the radius of gyration for all complexes indicated compactness of the protein due to ligand binding, which indicated enhanced stability due to the binding of lead compounds. The average number of H-bonds formed by Lead1, Lead2, Lead3, and the positive compound after stabilization of the system with the target enzyme were 1.8, 2.3, 0.6, and 6.5, respectively. Lead 3 intermittently formed weak hydrogen bonds in the simulation. The higher average hydrogen bonds for Lead 2 strongly supported better anchorage in the binding site, which meant improved inhibitory efficacy. The graphs showing RMSD, RMSF, radius of gyration, and average number of hydrogen bonds formed after system stabilization are presented in Fig. 2.

**Fig. 2:**
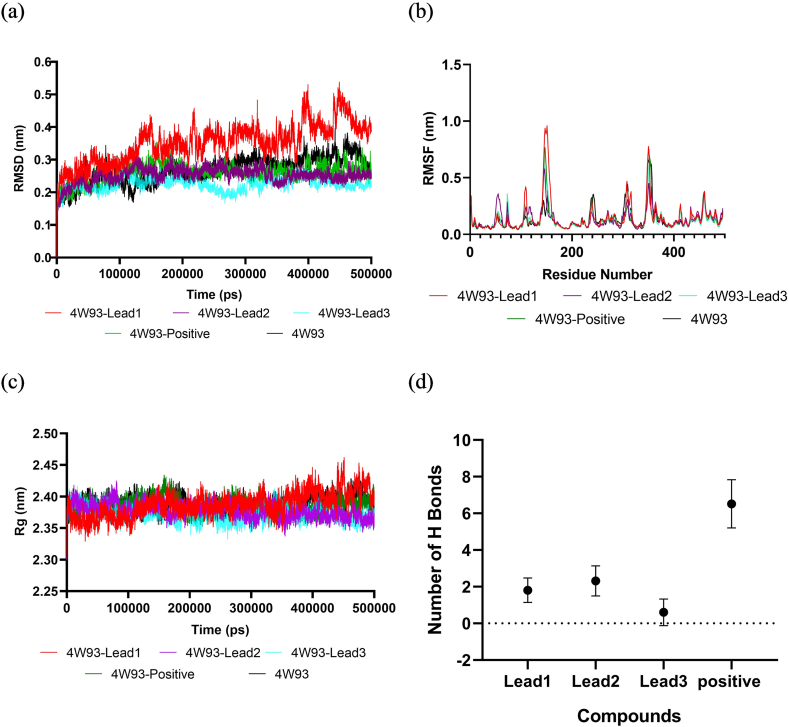
RMSD, RMSF, Radius of gyration, and average number of H-bonds plots. (a) RMSD values (nm) of unliganded 4W93 (black) enzyme and complexes 4W93-Lead1 (red), 4W93-Lead2 (magenta), 4W93-Lead3 (cyan), and 4W93-positive control, Acarbose (green) plotted against time in picoseconds (ps). (b) RMSF values (nm) of unliganded 4W93 (black) enzyme and complexes 4W93-Lead1 (red), 4W93-Lead2 (magenta), 4W93-Lead3 (cyan), and 4W93-positive control, Acarbose (green) plotted against the number of amino acid residues. (c) Radius of gyration values (nm) of unliganded 4W93 (black) enzyme and complexes 4W93-Lead1 (red), 4W93-Lead2 (magenta), 4W93-Lead3 (cyan), and 4W93-positive control, Acarbose (green) plotted against time in picoseconds (ps). (d) An average number of hydrogen bonds formed by Lead 1, Lead 2, Lead 3, and the positive control-Acarbose with the target enzyme after stabilization of the systems. (For interpretation of the references to color in this figure legend, the reader is referred to the Web version of this article.)

### 3.4 Binding energy calculation by MMPBSA and PCA

The binding energies of the three leads and the acarbose with the enzyme were derived using the gmx_MMPBSA tool (Table 3). Lead 2 showed the best binding energy of −82.71 ± 1.58 kJ/mol, followed closely by Lead 3 of binding energy −77.40 ± 5.56 kJ/mol when compared to other lead and the acarbose. The significantly more negative binding energy values for Lead 2 and Lead 3 compared to acarbose suggested that these leads inhibit α-amylase more strongly. The high negative Van der Waals (−109.11 ± 1.67 kJ/mol) and electrostatic energies (−88.49 ± 2.30 kJ/mol) for Lead 2 indicated a binding driven by both strong hydrophobic and polar interactions. PCA was carried out for all the complexes and apo protein to evaluate the conformational transitions of the protein. The scatter plots of the PCs and the free energy landscapes are represented in Fig. 3 and Fig. S3. The FEL showed the lowest energy minima with the Lead 2-protein complex than other leads.

**Table 3:** Thermodynamic calculation of binding energies for three leads and positive control.

Compound	Van der Waals energy (kJ/mol)	Electrostatic energy (kJ/mol)	Polar solvation energy (kJ/mol)	Non-polar solvation energy (kJ/mol)	Binding energy (kJ/mol)
**Lead 1**	−48.78 ± 2.30	−42.34 ± 3.01	59.07 ± 3.34	−6.06 ± 0.25	−38.11 ± 2.05
**Lead 2**	−109.11 ± 1.67	−88.49 ± 2.30	127.61 ± 2.30	−12.71 ± 0.12	**−82.71 ± 1.58**^a^
**Lead 3**	−164.64 ± 7.61	−47.61 ± 6.44	153.59 ± 9.79	−18.74 ± 0.66	**−77.40 ± 5.56**^a^
**Positive**	−146.35 ± 1.54	−132.17 ± 3.13	227.94 ± 2.97	−20.50 ± 0.12	−71.08 ± 1.67

*Values are in terms of mean ± SEM.

a Binding energy of Lead 2 and Lead 3 showed statistical significance when compared to positive, with a p-value <0.001 and α = 0.01.

**Fig. 3:**
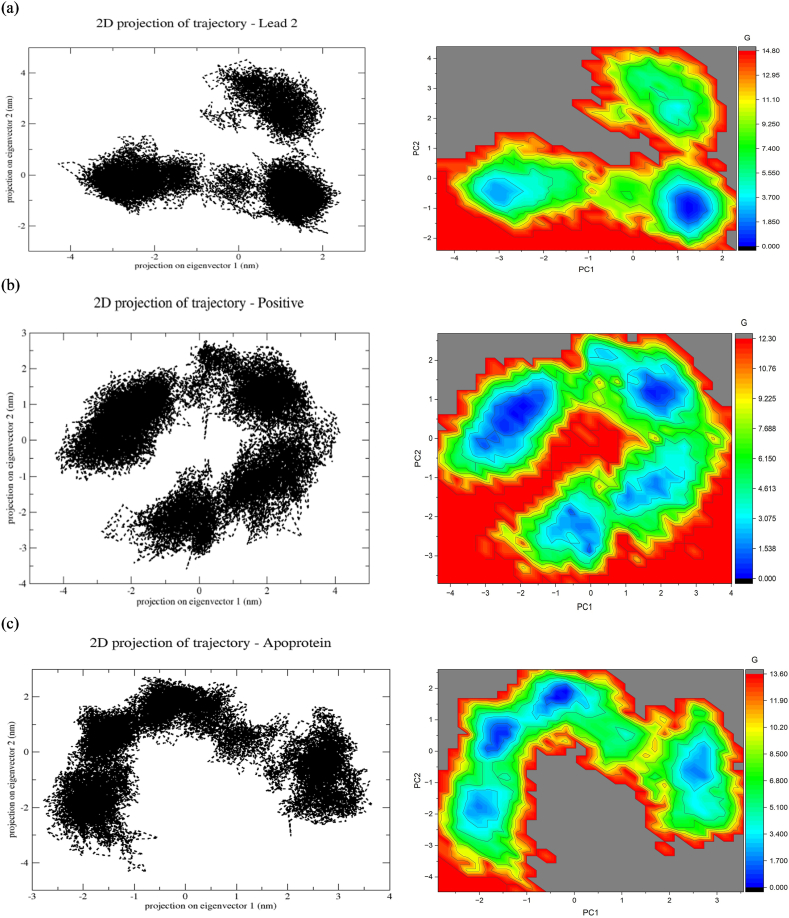
Principal component projection plot and Free energy landscape (FEL) of the protein (a) complexed with Lead 2, (b) complexed with positive control-acarbose, and (c) apoprotein.

### *3.5* *In vitro* enzyme inhibition assay

**Fig. 4:**
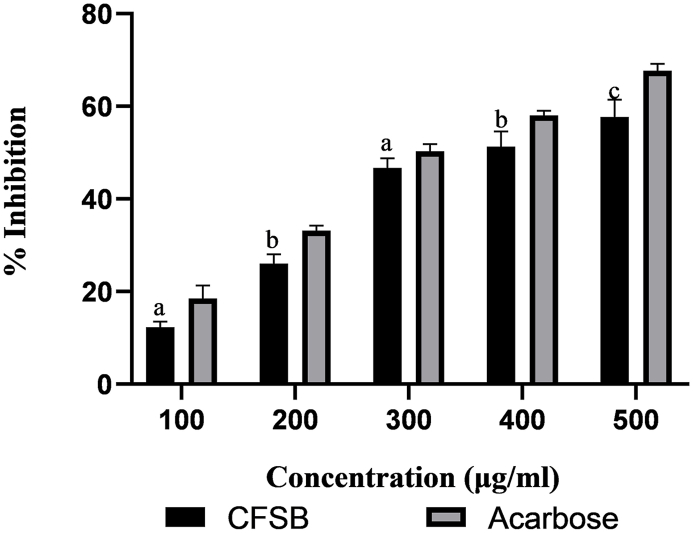
Percentage inhibition of α-amylase enzyme by CFSB and positive acarbose at different concentrations. % Inhibition plotted represents the mean of triplicate values calculated by ANOVA using Sidak's multiple comparison test with a significance level of 0.05 (α) and p-values of a ≤0.05, b ≤ 0.001, and c ≤ 0.001 for CFSB when compared to positive acarbose.

The potent α-amylase inhibitor Lead – 2 (2S,2′S,3S,3′S,4S)-3,3′,4,4′-Tetrahydro-2beta-(3,4-dihydroxyphenyl)-2′beta-(4-hydroxyphenyl)-4α,8′-bi[2H-1-benzopyran]-3beta,3′beta,5,5′,7,7' -hexol was identified to be present in CFSB as per PubChem records. CFSB, one of the components of MKC, was evaluated using an α-amylase inhibition assay. The CFSB extract showed concentration-dependent α-amylase inhibition. The percentage inhibition of α-amylase increased as the concentration of CFSB extract increased from 100 to 500 μg/ml. At the highest tested concentration (500 μg/ml), the CFSB extract exhibited 57% inhibition of α-amylase activity. The acarbose standard exhibited 67% of inhibition (Fig. 4).

**Fig. 5:**
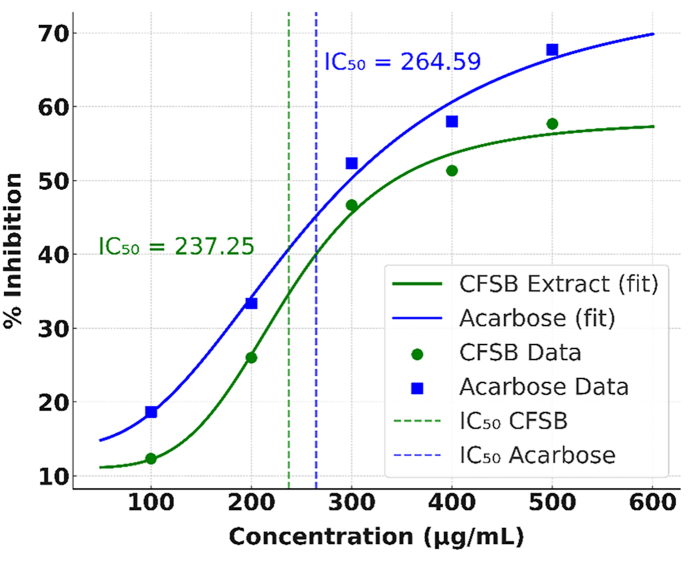
Dose–response curves for CFSB extract and the standard inhibitor acarbose against α-amylase enzyme activity. Data points represent the mean percentage inhibition values at increasing concentrations (100–500 μg/mL). Nonlinear regression analysis was performed using a four-parameter logistic model. Vertical dashed lines indicate the IC_50_ values for each compound: 237.25 μg/mL for CFSB extract and 264.59 μg/mL for acarbose.

As the relation between concentration of CFSB/Acarbose and % α-amylase inhibition did not show linearity, a nonlinear regression analysis was performed using a four-parameter logistic model to determine the IC_50_ values. The fitted curves displayed a sigmoidal dose-response profile for both CFSB extract and acarbose (Fig. 5). The estimated IC_50_ value for the CFSB extract was 237.25 μg/mL, whereas the IC_50_ for acarbose was slightly higher at 264.59 μg/mL, indicating a comparable inhibitory potential of the natural extract. The curve parameters revealed that the Hill slope (B) for CFSB (4.28) was steeper than that of acarbose (2.64), suggesting a sharper transition from low to high inhibition. The minimum and maximum asymptotes (A and D) for the CFSB extract were 11.08 and 58.14, respectively, while for acarbose, they were 14.07 and 76.24, respectively. The dose-response data were statistically significant with a good fit (R^2^ > 0.95) for both models.

## 4 Discussion

This study screened 915 bioactive compounds from five medicinal plants that are part of MKC and known for their antidiabetic properties. Three potential lead compounds demonstrating effective α-amylase inhibition were identified through *in silico* experiments. The top three compounds identified through molecular docking have demonstrated better binding affinities (−7.7 to −6.8 kcal/mol) than acarbose (−5.5 kcal/mol), suggesting their potential as more effective alternatives to synthetic α-amylase inhibitors. Acarbose was chosen as the positive control because it is a naturally derived α-amylase inhibitor, and it also showed less probability of carcinogenicity compared to miglitol and voglibose. Several studies have successfully incorporated similar approaches to find better alternatives to acarbose, such as triazole derivatives [41], Oxadiazole derivatives [42], Piperidine-substituted chalk ones [43], and imidazo derivatives [44]. However, the need for an effective novel α-amylase inhibitor without significant side effects still exists. Docking studies showed that most lead compounds form interactions with specific residues, such as GLN302, GLY304, ASP317, ALA310, ARG267, and PHE348. GLY304 and ALA310 are part of a flexible loop near the active site, which is crucial for substrate binding and enzyme inhibition [45]. ASP317 often acts as a nucleophile or is involved in substrate binding, as observed with other aspartic residues in similar studies [46]. Aromatic residues like PHE348 are known to engage in π-π interactions, which can be crucial for the binding of inhibitors to the active site of the enzyme, preventing access to substrates [47]. Binding pose analysis of lead compounds with α-amylase has revealed various non-covalent interactions. Van der Waals forces are fundamental in the formation of protein-ligand complexes, which are modeled using the Lennard-Jones potential during virtual screening of small organic molecules [48]. Hydrogen bonds are crucial for the stable protein-ligand binding in the active site of proteins [49]. Carbon-hydrogen bonds are often found in catalytic sites, probably solvent-shielded, which enhances binding stability and maintains active site geometry [50]. π-stacking interactions are also well documented in medicinal chemistry for their role in molecular recognition and drug design [51]. The strong binding affinities combined with well-defined interaction networks with the critical residues by hydrophobic and polar interactions, suggest that these compounds could effectively inhibit α-amylase activity by blocking the active site.

Despite acarbose being an established α-amylase inhibitor, ADME/Tox evaluation has shown poor druglikeness, high synthetic complexity, and higher toxicity predictions, emphasizing the need for safer novel leads. ADME/Tox evaluation of lead compounds has revealed favourable pharmacokinetic profiles. Lead 1 displays high GI absorption, whereas Leads 2, 3, and acarbose are classified as having low GI absorption. This difference indicates that Lead 1 could show better bioavailability than Lead 2 or Lead 3. However, Lead 1 is a P-gp substrate, which could lead to reduced intracellular concentrations, while Lead 2 and Lead 3 are not P-gp substrates, which may effectively enhance their bioavailability. All compounds, including the leads and acarbose, are predicted as BBB non permeators. This property is desirable as drugs intended for peripheral targets, such as α-amylase inhibitors, should ideally not cross the BBB to avoid CNS-related side effects [52,53]. In addition, the predicted drug-like properties were better for the leads when compared to the positive control, acarbose. The synthetic accessibility scores for the top three leads (between 4.99 and 5.37) are better than that of acarbose (7.34). This implies that the leads may possess simple structures and be more amenable to synthesis, an important factor for cost-effective drug development [54]. Furthermore, the toxicity prediction indicates that Lead 2 and Lead 3 might have considerably lower drug-induced liver injury potential (0.151 and 0.019, respectively) compared to acarbose (0.689). Similarly, their predicted acute oral toxicity and carcinogenicity values are more favourable [26]. These properties are crucial for the development of effective oral antidiabetic drugs with minimal adverse effects.

The results of the MD simulations of protein-ligand complexes further validated the stability within the active site of α-amylase. Generally, RMSD values should be less than 3 Å in MD simulations [55]. In this study, all complexes showed lower RMSD, particularly Lead 2, which showed RMSD within 2 Å and consistent interactions with important amino acids, indicating the formation of a stable complex. Lower RMSD values suggest that the binding of Lead 2 helps stabilize the conformation of the enzyme, thereby reducing fluctuations from the reference structure over time [28]. The lower RMSF values of Lead 2 compared to the unliganded enzyme also indicate reduced flexibility, which is often associated with increased binding affinity and specificity [56]. The reduced RMSD and RMSF values exhibited by bioactive compounds from medicinal plants have been shown to demonstrate enhanced stability and efficacy in enzyme inhibition [57]. The combined MD simulation results, such as lower RMSD and RMSF values, reduced Rg, and increased hydrogen bond count, indicate that the binding of lead compounds, particularly Lead 2, significantly stabilizes the enzyme structure compared to the unbound state. This stabilization is vital for effective enzyme inhibition, as it prevents conformational fluctuations that are necessary for catalytic activity.

Statistical analysis of the MM-PBSA binding energy components revealed the superiority of the lead compounds over the positive control, acarbose. Descriptive statistics showed that Lead 2 exhibited the most favourable net binding energy (−82.71 ± 1.58 kJ/mol), followed by Lead 3 (−77.40 ± 5.56 kJ/mol), while acarbose displayed a comparatively weaker binding energy (−71.08 ± 1.67 kJ/mol).

Independent sample t-tests showed that the net binding energy of Lead 2 was significantly more favourable than that of acarbose (p < 0.01, α = 0.05). Similarly, a one-way ANOVA across all leads and the control confirmed a statistically significant difference in the binding energies (F (3, 10.75) = 703.43, p < 0.001). Post hoc Tukey's test proved that Lead 2 and Lead 3 differed significantly from acarbose. These analyses support the thermodynamic inference that Lead 2, followed by Lead 3, form more stable, energetically favourable, and hydrophobically anchored complexes with α-amylase than with acarbose. The breakdown of Van der Waals and electrostatic contributions indicates that non-covalent interactions have a pivotal role in the stabilization of enzyme-ligand complex [58]. A previous study evaluated the α-amylase inhibitory activity of bioactive compounds, including ellagic acid, epicatechin, isocolumbin, luteolin, and shahidine, using MD simulation studies. They showed binding energies in the range between −10.237 kJ/mol and −59.681 kJ/mol [59].

PCA indicates that ligand binding strongly modulates protein dynamics. The Lead 2–protein complex forms a tight cluster, showing restricted motions and high structural stability, while the positive control shows moderate flexibility, and the apo-protein displays wide dispersion, reflecting high conformational mobility. The free energy landscape reveals deep low-energy minima for the Lead 2-protein complex, confirming a stable and energetically favourable conformation.

*In vitro* α-amylase inhibition assay corroborates the *in silico* findings, with *Cassia fistula* containing Lead 2 exhibiting significant inhibition (57 % at 500 μg/ml) at lower concentrations of crude extract, which is comparable to pure acarbose (68 %). Nonlinear regression analysis using a four-parameter logistic model determined the calculated IC_50_ of 237.25 μg/mL for CFSB, indicating a strong dose-dependent inhibition profile, supported by a steeper Hill slope compared to acarbose, which may reflect more efficient enzyme–ligand interaction. Previous studies have shown that *Cassia fistula* exhibits diverse medicinal properties, including hepatoprotective, antipyretic, antioxidant, anti-inflammatory, antimicrobial, and wound-healing effects. Its extracts have shown potential anticancer, antidiabetic, antiepileptic, and CNS-modulating activities. Additionally, it alleviates skin irritations and promotes overall therapeutic benefits [60]. In this study, *Cassia fistula* showed effective α-amylase inhibition at lower concentrations, while a previous study indicated an α-amylase inhibitory effect at a dose of 400 mg [61]. Other plant-based phytochemicals, such as acetyleugenol, apigenin, cinnamic acid, eriodictyol, myrcene, piperine, and rosmarinic acid, have shown varying degrees of α-amylase inhibition. Therefore, it is evident that the plant extracts could be a better alternative for synthetic drugs in α-amylase inhibition [62]. In the Ayurveda system of medicine, *Cassia* sp. is extensively used in the treatment of headache and fever [63]. Additionally, the *Cassia fistula* leaves paste is identified to reduce itching, oozing, burning sensation, eruptions, discoloration, pain, and inflammation in individuals with eczema [64]. Based on these, it was proposed that *Cassia fistula* could be used to heal diabetic wounds.

Previous reports have shown that a major class of α-amylase inhibitors are plant metabolites, such as flavonoids and tannins, which have exhibited a variety of mechanisms of action [2]. Supporting this, the Lead 2 compound, a potent α-amylase inhibitor proposed in this study, is known to be a proanthocyanin belonging to the flavonoid class [20]. Since the positive control, Acarbose works by competitive inhibition of α-amylase, and all the *in silico* properties and the enzyme inhibition assay of lead 2 exhibit similarity to Acarbose, it is proposed that the lead 2 also likely acts by competitive inhibition of α-amylase, which prevents the binding of starch to the enzyme and delays the breakdown of starch into glucose and maltose, thereby delaying glucose absorption and reducing postprandial hyperglycemia in diabetes mellitus.

Although acarbose exhibits better inhibition, its known side effects warrant the exploration of safer alternatives [65]. The combination of computational and experimental approaches used in this study provides a robust framework for identifying natural α-amylase inhibitors that could be further developed into antidiabetic therapeutics, as suggested by previous studies [2].

The overall findings of this study suggest that Lead 2 possesses significant potential as a safe and effective α-amylase inhibitor. Further research should focus on structure optimization, pharmacokinetic and toxicological assessments, mechanistic investigation, and preclinical/clinical trials to validate therapeutic potential. Additionally, sustainable harvesting and large-scale purification of *Cassia fistula* could facilitate its development as a commercial therapeutic agent, contributing to diabetes management strategies.

## 5 Conclusion

This study successfully identified three potential α-amylase inhibitors from medicinal plants known for their antidiabetic properties and evaluated the best lead compound. Using an integrative approach combining *in silico* molecular docking, ADME/Tox analysis, MD simulations, and *in vitro* inhibition assays, (2S,2′S,3S,3′S,4S)-3,3′,4,4′-tetrahydro-2beta-(3,4-dihydroxyphenyl)-2′beta-(4-hydroxyphenyl)-4α,8′-bi[2H-1-benzopyran]-beta,3′beta,5,5′,7,7′-hexol was identified as a promising lead compound. *In vitro* α-amylase inhibition assays further validated the efficacy of the plant *Cassia fistula* containing Lead 2. The crude extract showed an inhibitory activity of 57 %, comparable to the standard acarbose (67 %). IC_50_ values for the *Cassia fistula* stem bark (CFSB) extract and acarbose were calculated as 237.25 μg/mL and 264.59 μg/mL, respectively, using a nonlinear regression approach, indicating a comparable inhibitory potential of the natural extract, yet with a safer pharmacokinetic profile. Further purification, optimization, and clinical studies are essential to develop this compound as a potent therapeutic option for diabetes.

## Data availability statement

Upon request, the data supporting this study are available from the corresponding author.

## Author contributions

RB – Investigation, Resources, Writing – original draft; MH – Investigation, Writing – original draft; SSS – Investigation, Writing – review & editing; TH – Formal Analysis, Writing – review & editing; AR – Formal analysis, Writing – review & editing; KK – Validation, Writing – review & editing; RP – Validation, Data curation, Writing – review & editing; BR – Conceptualization, Supervision, Validation, Writing – review & editing.

## Declaration of AI-assisted technologies in the writing process

During the preparation of this manuscript, the authors used Grammarly™ and Paperpal™ tools for grammar checks and polishing the language. The authors reviewed and edited the content and take full responsibility for the accuracy and integrity of the manuscript.

## Funding details

This research did not receive any specific grant from funding agencies in the public, commercial, or not-for-profit sectors.

## Conflict of interest

The authors declare that they have no known competing financial interests or personal relationships that could have appeared to influence the work reported in this paper.

## Figures and Tables

**figs1:**
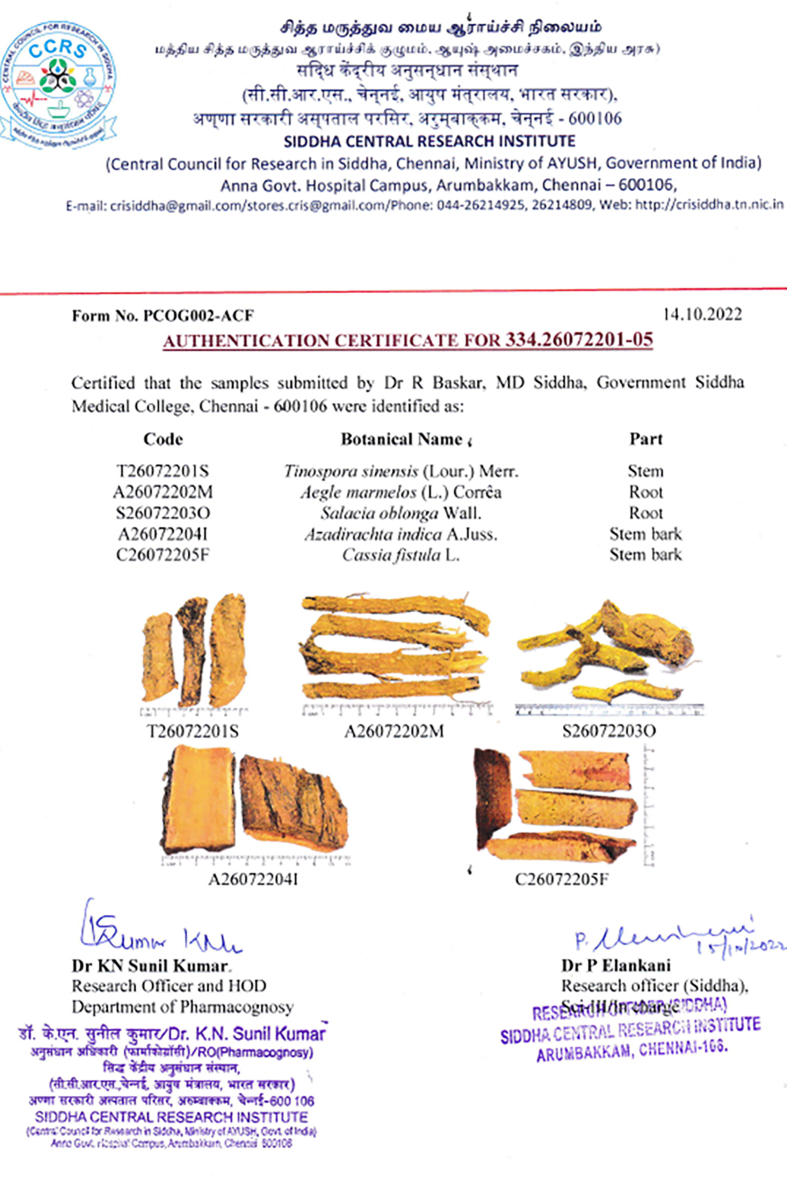


**figs2:**
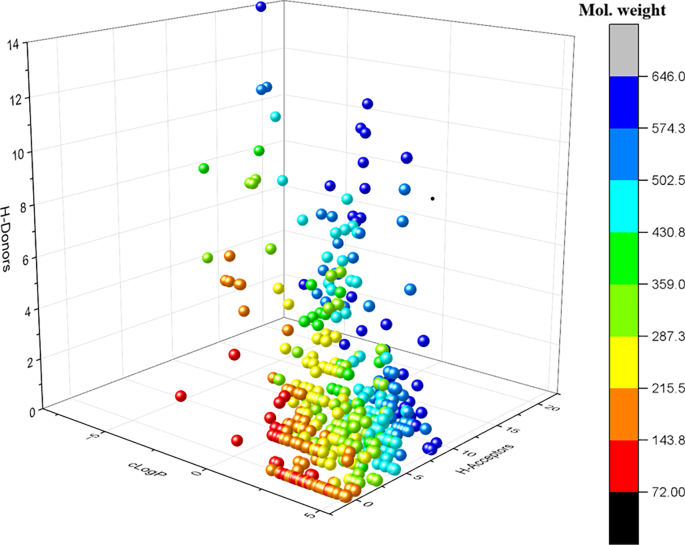


**figs3:**
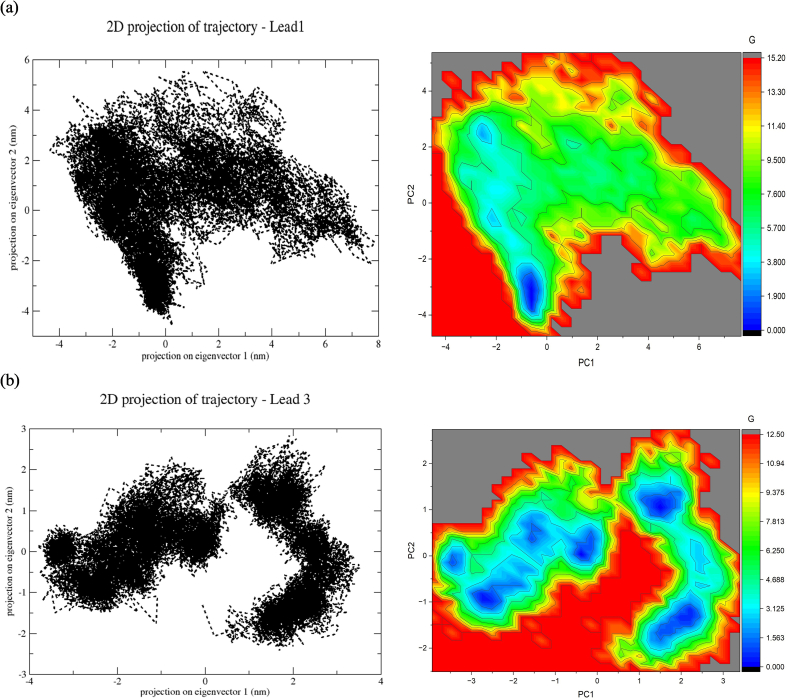

