# Supplementary material for: Antidiabetic potential of a novel hydroxyphenyl-bi-benzopyran-hexol compound from Cassia fistula as an α-amylase inhibitor: Integrated in silico screening and in vitro validation using a nonlinear regression model
Source: J Ayurveda Integr Med. 2026 Jul 24;17(4):101366. doi: 10.1016/j.jaim.2026.101366 (PMC13420717; doi:10.1016/j.jaim.2026.101366)
Supplement: Multimedia component 1 [file mmc1.docx]

**Table S1** List of natural compounds with PubChem ID

| **Compound ID** | **Compound Name** |
| --- | --- |
| 305 | Choline |
| 335 | o-Cresol |
| 342 | m-Cresol |
| 370 | Gallic Acid |
| 460 | Guaiacol |
| 753 | Glycerol |
| 860 | 9,12,15-Octadecatrienoic acid |
| 965 | 9-Octadecenoic acid |
| 1183 | Vanillin |
| 1203 | L-Epicatechin |
| 2263 | Dimethyl 10-(acetyloxy)-3,5-dihydroxy-4-(6a-hydroxy-7a-methyl-3a,6a,7,7a-tetrahydro-2,7-methanofuro[2,3-b]oxireno[e]oxepin-1a(2H)-yl)-4-methyl-8-[(2-methylbut-2-enoyl)oxy]octahydro-1H,7H-naphtho[1,8a-c:4,5-b'c']difuran-5,10a(8H)-dicarboxylate |
| 2353 | Berberine |
| 2355 | Bergapten |
| 2879 | P-Cresol |
| 3220 | Emodin |
| 3248 | 3-(2,5-Dihydroxyphenyl)-2-propenoic acid methyl ester |
| 3893 | Lauric Acid |
| 3931 | 9,12-Octadecadienoic acid |
| 4114 | Methoxsalen |
| 5793 | D-Glucose |
| 5984 | D-(-)-Fructose |
| 5988 | Sucrose |
| 5997 | Cholesterol |
| 6054 | 2-Phenylethanol |
| 6214 | Hexachloroethane |
| 6466 | Gibberellic acid |
| 6549 | Linalool |
| 6654 | alpha-PINENE |
| 6656 | 2-Butenoic acid, 2-methyl- |
| 6760 | Skimmianine |
| 6923 | 2-Tert-butylphenol |
| 6987 | Piperitone |
| 7213 | 2,4-Dihydroxybenzaldehyde |
| 7460 | alpha-PHELLANDRENE |
| 7461 | gamma-Terpinene |
| 7463 | P-Cymene |
| 7478 | 4-Methoxybenzoic acid |
| 7800 | Ethyl dodecanoate |
| 8163 | 2-Undecanone |
| 8181 | Methyl palmitate |
| 8193 | 1-Dodecanol |
| 8201 | Methyl stearate |
| 8203 | Methyl octadeca-9,12-dienoate |
| 8222 | Eicosane |
| 8417 | Scoparone |
| 8468 | Vanillic acid |
| 9064 | Cianidanol |
| 10168 | Rhein |
| 10205 | Plumbagin |
| 10207 | Aloe-emodin |
| 10208 | Chrysophanol |
| 10212 | Imperatorin |
| 10393 | 2-(4-Hydroxyphenyl)ethanol |
| 10416 | Malvalic acid |
| 10469 | Hexacosanoic acid |
| 10582 | Myrtenol |
| 10639 | Physcion |
| 10964 | Malonaldehyde |
| 11005 | Myristic acid |
| 11197 | Tetracosanoic acid |
| 11230 | 4-Carvomenthenol |
| 11463 | Terpinolene |
| 11622 | 2-Tridecanone |
| 11636 | Heptacosane |
| 11850 | Galactitol |
| 12177 | 2-Methylpent-2-enal |
| 12366 | Ethyl palmitate |
| 12377 | Dipropyl disulfide |
| 12405 | Docosane |
| 12409 | Nonacosane |
| 12410 | Hentriacontane |
| 12575 | 3-Hydroxy-4-methoxybenzoic acid |
| 12921 | Sterculic acid |
| 13187 | 2-Nonanone |
| 14896 | beta-Pinene |
| 16592 | Methyl propyl disulfide |
| 18818 | Sabinene |
| 19009 | Palmatine |
| 22311 | Limonene |
| 22383 | Dipropyl trisulfide |
| 26305 | Nodakenetin |
| 26519 | Tetratriacontane |
| 31238 | Tridecylbenzene |
| 31245 | 2-Methylpentanal |
| 31253 | Myrcene |
| 31289 | Nonanal |
| 31291 | Tetradecanal |
| 35349 | Ethyl propyl disulfide |
| 60961 | Adenosine |
| 64971 | Betulinic Acid |
| 65084 | (+)-Gallocatechin |
| 68066 | Atranorin |
| 68171 | 1-Hexacosanol |
| 68406 | 1-Octacosanol |
| 69502 | Alloimperatorin |
| 69894 | Isoscopoletin |
| 70954 | Cyclohexene, 1-methyl-4-(1-methylethyl)-, (R)- |
| 72276 | (-)-Epicatechin |
| 72277 | Epigallocatechin |
| 73111 | Sennoside A |
| 73145 | beta-Amyrin |
| 73170 | alpha-Amyrin |
| 73337 | Magnoflorine |
| 74138 | 1-Docosene |
| 75997 | Nonacosan-15-one |
| 76015 | 10-Undecyn-1-ol |
| 76295 | 1,4,5-Trihydroxyanthraquinone |
| 77409 | 7H-Furo[3,2-g][1]benzopyran-7-one, 2,3-dihydro-9-methoxy- |
| 79089 | 3,4-Dimethylthiophene |
| 80048 | alpha-Elemene |
| 81696 | alpha-D-Talopyranose |
| 83412 | 6-Methoxymellein |
| 91440 | Sennoside B |
| 92139 | alpha-Curcumene |
| 92221 | (+)-Camphene |
| 94162 | Sugiol |
| 94221 | (-)-trans-Carveol |
| 94249 | 2-Methyldecalin |
| 94403 | Farnesyl acetate |
| 98570 | Allocryptopine |
| 100017 | Nimbolide |
| 101977 | D-Citronellol |
| 104285 | Dipropyl tetrasulfide |
| 104884 | [(1R,2S,4R,6R,9R,10R,11R,12S,14R,15R,18R)-14-acetyloxy-6-(furan-3-yl)-10-(2-methoxy-2-oxoethyl)-7,9,11,15-tetramethyl-3,17-dioxapentacyclo[9.6.1.02,9.04,8.015,18]octadec-7-en-12-yl] 2-methylbut-2-enoate |
| 106648 | 2,3-Dihydro-9-hydroxy-7H-furo[3,2-g][1]benzopyran-7-one |
| 107526 | (2R,3S,4R,5R)-2,3,4,5,6-pentahydroxyhexanal |
| 107936 | gamma-Fagarine |
| 108058 | Nimbin |
| 122738 | Procyanidin B2 |
| 125213 | N-feruloyltyramine; Moupinamide |
| 125468 | Tiglic acid |
| 126566 | Mahmoodin |
| 129754 | Naheedin |
| 138824 | 3,7,11-Trimethyl-1-dodecanol |
| 157277 | Nimbandiol |
| 159931 | Zeorin |
| 167718 | Tembetarine |
| 173183 | Campesterol |
| 176920 | 4'-Methyl-epigallocatechin |
| 176996 | 6-Desacetylnimbin |
| 177090 | Nimbosone |
| 178770 | Nimocinol |
| 180429 | Nimolinin |
| 180932 | Palmatoside E |
| 184937 | Nimolicinol |
| 188289 | Unii-kki91P85GE |
| 189403 | 12-Hydroxy-13-methylpodocarpa-8,11,13-triene-3,7-dione |
| 189404 | 2,9(1H,3H)-Phenanthrenedione, 4,4a,10,10a-tetrahydro-7-hydroxy-1,1,4a,6-tetramethyl-, (4aS-trans)- |
| 189660 | 12,13-Dimethoxypodocarpa-8(14),9(11),12-triene-3,7-dione |
| 189704 | Nimbionol |
| 189706 | Nimbionone |
| 189726 | Margolonone |
| 189727 | (4bS,8aR)-2,4b,8,8-tetramethyl-7,10-dioxo-5,6,8a,9-tetrahydrophenanthrene-3-carboxylic Acid |
| 189728 | 12-Methyl-7-oxopodocarpa-8,11,13-triene-13-carboxylic acid |
| 193405 | Cassine |
| 216283 | Ammijin |
| 222284 | Beta-Sitosterol |
| 237332 | 5-Hydroxymethylfurfural |
| 259846 | Lupeol |
| 267137 | 4-(3-Methylbut-2-enoxy)benzoic acid |
| 283510 | 2-Methyltricosane |
| 304040 | Butyl p-tolyl sulfide |
| 330573 | (-)-cis-Carveol |
| 334704 | Marmesin |
| 361512 | Citreorosein |
| 400073 | 2H-1-Benzopyran-2-one, 7-[[(2E)-3,7-dimethyl-2,6-octadienyl]oxy]- |
| 439242 | Raffinose |
| 439357 | Alpha-D-Galactopyranose |
| 439503 | Salicin |
| 439531 | Stachyose |
| 440917 | D-Limonene |
| 440967 | (-)-beta-Pinene |
| 440968 | (-)-alpha-Pinene |
| 441005 | (+)-delta-Cadinene |
| 442015 | (2S,4AR,6aR,7R,10R,10aS,10bS)-2-(furan-3-yl)-7-hydroxy-6a,10b-dimethyl-4a,5,6,6a,7,10,10a,10b-octahydro-1H-10,7-(epoxymethano)benzo[f]isochromene-4,12(2H)-dione |
| 442068 | Palmarin |
| 442127 | Decursinol |
| 442153 | Abyssinone V |
| 442154 | Afzelechin |
| 442359 | alpha-Cubebene |
| 442731 | Pulmatin |
| 443158 | (-)-Linalool |
| 443639 | (-)-Epiafzelechin |
| 444539 | Cinnamic acid |
| 444899 | Arachidonic acid |
| 445638 | Palmitoleic acid |
| 445639 | Oleic Acid |
| 480764 | 8-Prenylnaringenin |
| 484588 | Euchrestaflavanone A |
| 500060 | [17-(3-furyl)-4,4,8,10,13-pentamethyl-3,16-dioxo-6,7,9,11,12,17-hexahydro-5H-cyclopenta[a]phenanthren-7-yl] acetate |
| 518975 | 8-Isopropyl-2,5-dimethyl-1,2,3,4-tetrahydronaphthalene |
| 520895 | 3,5-Diethyl-1,2,4-trithiolane |
| 521941 | Methyl butyl disulfide |
| 522458 | Butyl propyl disulfide |
| 525330 | Allyl propyl trisulfide |
| 529904 | Propenyl propyl disulfide |
| 575174 | Cassin |
| 584354 | 6-Methyl-4-chromanone |
| 595524 | Furan, 5-methyl-2,2'-methylenedi- |
| 600671 | Aegelinol |
| 611513 | Marmesin galactoside |
| 636663 | 5-(2-Hydroxyphenoxymethyl)furfural |
| 636837 | 22,23-Dihydronimocinol |
| 638072 | Squalene |
| 643654 | Malyngamide S |
| 1550607 | Auraptene |
| 1713001 | Nerylacetone |
| 1742210 | Caryophyllene oxide |
| 1795390 | 7-[[(2Z)-3,7-Dimethyl-2,6-octadienyl]oxy]-2H-1-benzopyran-2-one |
| 1810796 | (6S,10R)-6,10,14-trimethylpentadecan-2-one |
| 2723872 | D-Fructose |
| 3034112 | Deacetylgedunin |
| 3084213 | 7-[(6R)-6,7-dihydroxy-3,7-dimethyloct-2-enoxy]chromen-2-one |
| 4482272 | CID 4482272 |
| 5260170 | [6-(Furan-3-yl)-14-hydroxy-10-(2-methoxy-2-oxoethyl)-7,9,11,15-tetramethyl-3,17-dioxapentacyclo[9.6.1.02,9.04,8.015,18]octadec-7-en-12-yl] 2-methylbut-2-enoate |
| 5280343 | Quercetin |
| 5280435 | Phytol |
| 5280443 | Apigenin |
| 5280450 | Linoleic Acid |
| 5280459 | Quercitrin |
| 5280460 | Scopoletin |
| 5280489 | Beta-Carotene |
| 5280537 | Moupinamide |
| 5280704 | Cosmosiin |
| 5280794 | Stigmasterol |
| 5280804 | Isoquercitrin |
| 5280805 | Rutin |
| 5280862 | Isokaempferide |
| 5280863 | Kaempferol |
| 5280934 | Linolenic Acid |
| 5281119 | Myristoleic acid |
| 5281303 | Azadirachtin |
| 5281310 | [(1R,2S,4R,6R,9R,10S,11R,12S,14R,15R,18R)-14-Acetyloxy-6-(furan-3-yl)-10-(2-methoxy-2-oxoethyl)-7,9,11,15-tetramethyl-3,17-dioxapentacyclo[9.6.1.02,9.04,8.015,18]octadec-7-en-12-yl] (E)-2-methylbut-2-enoate |
| 5281426 | Umbelliferone |
| 5281515 | Caryophyllene |
| 5281520 | Humulene |
| 5281522 | Isocaryophyllene |
| 5281553 | beta-Ocimene |
| 5281672 | Myricetin |
| 5281876 | 3-Acetyl-1-tigloylazadirachtinin |
| 5282102 | Astragalin |
| 5282822 | Octadeca-9,12,15-trienoic acid |
| 5283640 | 24-Methylenelophenol |
| 5284421 | Methyl linoleate |
| 5288340 | 3-O-beta-D-galactopyranosyl-beta-D-galactopyranose |
| 5288377 | 3-O-beta-D-glucopyranuronosyl-beta-D-galactopyranose |
| 5315396 | Yinyanghuo D |
| 5315851 | 2-hydroxy-3-methyl-1-[(2S,3R,4S,5S,6R)-3,4,5-trihydroxy-6-(hydroxymethyl)oxan-2-yl]oxyanthracene-9,10-dione |
| 5316525 | Demethylsuberosin |
| 5316800 | 1,5-Dihydroxy-3-methylanthraquinone |
| 5316860 | Syringin |
| 5316891 | 7-Hydroxy-2,5-dimethyl-4H-1-benzopyran-4-one |
| 5318565 | Isofraxidin |
| 5318767 | Nicotiflorin |
| 5319336 | [(10R)-17-(furan-3-yl)-7-hydroxy-4,4,10,13-tetramethyl-3-oxo-2,5,6,7,8,9,11,12,16,17-decahydro-1H-cyclopenta[a]phenanthren-6-yl] acetate |
| 5319406 | 7-Geranyloxy-6-methoxycoumarin |
| 5319500 | Cassiachromone |
| 5319706 | Methyl linolenate |
| 5319765 | Methyl propyl trisulfide |
| 5320171 | [(1R,11R,16R)-17,19-diacetyloxy-8-(furan-3-yl)-4-hydroxy-1,9,11,16-tetramethyl-5,14-dioxapentacyclo[11.6.1.02,11.06,10.016,20]icos-9-en-12-yl] (E)-3-phenylprop-2-enoate |
| 5320250 | (Z)-beta-Ocimene |
| 5352019 | (2R,3R)-3-(Benzoylamino)-2-hydroxy-3-phenylpropionic acid 1,7beta-dihydroxy-2alpha-(benzoyloxy)-4,10beta-diacetoxy-9-oxo-5beta,20-epoxytaxa-11-ene-13alpha-yl ester |
| 5353609 | Methyl 2,5-dihydroxycinnamate |
| 5363269 | Ethyl oleate |
| 5367462 | 9,12,15-Octadecatrienoic acid, methyl ester |
| 5459840 | 20-Hydroxyecdysone |
| 5742590 | Sitogluside |
| 6324923 | Chrysophanein |
| 6434062 | Octatriene, dimethyl- |
| 6437066 | Salannin |
| 6442484 | Nimbilin |
| 6442906 | (+)-Nimocinolide |
| 6450230 | Marmin |
| 6451151 | Salacinol |
| 6451598 | 12-Methoxy-13-methylpodocarpa-8,11,13-trien-7-one |
| 6453932 | (4aS,6aR,6aS,6bR,10S,12aR,14bR)-10-[(2S,3R,4S,5S)-4,5-dihydroxy-3-[(2R,3R,4S,5S,6R)-3,4,5-trihydroxy-6-(hydroxymethyl)oxan-2-yl]oxyoxan-2-yl]oxy-2,2,6a,6b,9,9,12a-heptamethyl-1,3,4,5,6,6a,7,8,8a,10,11,12,13,14b-tetradecahydropicene-4a-carboxylic acid |
| 6482976 | N-[(2S)-2-hydroxy-2-(4-methoxyphenyl)ethyl]benzamide |
| 6918743 | [1-[(2R,3S,4S)-3,4-dihydroxy-2-(hydroxymethyl)thiolan-1-ium-1-yl]-2,4,5,6,7-pentahydroxyheptan-3-yl] sulfate |
| 9548705 | Germacrene a |
| 9796891 | Epoxyaurapten |
| 10031185 | (2R,3R,4S,5S,6R)-2-[[(1R,2S,3S)-7-hydroxy-1-(4-hydroxy-3,5-dimethoxyphenyl)-3-(hydroxymethyl)-6,8-dimethoxy-1,2,3,4-tetrahydronaphthalen-2-yl]methoxy]-6-(hydroxymethyl)oxane-3,4,5-triol |
| 10065647 | 2,9,10-Trimethoxy-5,6-dihydroisoquinolino[2,1-b]isoquinolin-3-one |
| 10098738 | Cordifoliside D |
| 10108651 | (R)-Aegeline |
| 10181133 | Cerevisterol |
| 10212035 | 2-(2'-Hydroxytetracosanoylamino)-octadecane-1,3,4-triol |
| 10239837 | Epiafzelechin-(4beta-8)-epicatechin |
| 10263440 | Epiprocurcumenol |
| 10348278 | (2R,3R,4S,5S,6R)-2-[[(1R,2R,3R)-7-hydroxy-1-(4-hydroxy-3,5-dimethoxyphenyl)-3-(hydroxymethyl)-6,8-dimethoxy-1,2,3,4-tetrahydronaphthalen-2-yl]methoxy]-6-(hydroxymethyl)oxane-3,4,5-triol |
| 10392456 | Cordifoliside E |
| 10438246 | (-)-2\|A-O-(\|A-D-Glucopyranosyl)lyoniresinol |
| 10442609 | (3R)-3-Methyl-5,8-dihydroxy-3,4-dihydro-1H-2-benzopyran-1-one |
| 10483388 | (+)-lyoniresinol-3-alpha-O-beta-D-glucopyranoside |
| 10505484 | 6-Deacetylnimbin |
| 10601920 | Kotalagenin 16-acetate |
| 10628287 | 11-epi azadirachtin D |
| 10767085 | 22-epi-20-Hydroxyecdysone |
| 10884852 | 2-(beta-D-Glucopyranosyloxy)-3,6-dimethoxybenzoic acid benzyl ester |
| 10906239 | Azadirone |
| 10977864 | 7-Hydroxy-2-[(S)-2-hydroxypropyl]-5-(hydroxymethyl)-4H-1-benzopyran-4-one |
| 11077057 | Benzyl 2-hydroxy-3,6-dimethoxybenzoate |
| 11087935 | Montanine |
| 11088324 | 7-[[(2E)-5-[(R)-3,3-Dimethyloxiranyl]-3-methyl-2-pentenyl]oxy]-2H-1-benzopyran-2-one |
| 11095397 | 4-Nitroindole-3-carboxaldehyde |
| 11119228 | Nimbiol |
| 11192900 | (5S,12S,13S,16S)-12-(4,4-dichloropentyl)-5-hydroxy-16-(2-hydroxypropan-2-yl)-4,4,13-trimethyl-3,11,15-trioxa-7,18-dithia-20,21-diazatricyclo[15.2.1.16,9]henicosa-1(19),6(21),8,17(20)-tetraene-2,10,14-trione |
| 11209134 | (E)-N-[(2S)-2-methoxy-2-(4-methoxyphenyl)ethyl]-3-phenylprop-2-enamide |
| 11243273 | (3R,6R)-3-Isopropyl-6-(4-methoxybenzyl)-4-methylmorpholine-2,5-dione |
| 11334829 | Nimbidiol |
| 11403749 | (S)-Aegeline |
| 11482406 | Xanthoarnol |
| 11767849 | Cerebroside C |
| 11770062 | Cubenol |
| 11798426 | Azadiradionolide |
| 11813223 | 7-[(E,4R,6R)-4,7-dihydroxy-3,7-dimethyl-6-[(2S,3R,4S,5S,6R)-3,4,5-trihydroxy-6-(hydroxymethyl)oxan-2-yl]oxyoct-2-enoxy]chromen-2-one |
| 11818411 | (E)-N-[(2S)-2-hydroxy-2-(4-hydroxyphenyl)ethyl]-3-phenylprop-2-enamide |
| 11870456 | (3S,8R,9R,10R,13R,14R,17R)-17-[(2R,5R)-5-ethyl-6-methylheptan-2-yl]-10,13-dimethyl-2,3,4,7,8,9,11,12,14,15,16,17-dodecahydro-1H-cyclopenta[a]phenanthren-3-ol |
| 12004512 | Gedunin |
| 12011153 | 7-Deacetylnimolicinol |
| 12046149 | Epicubenol |
| 12069125 | 17-Hydroxynimbocinol |
| 12072821 | O-Methylazadironolide |
| 12272224 | 2,5-Dimethyl-7-methoxychromone |
| 12302222 | tau-Cadinol |
| 12302226 | Amorphen-10-ol |
| 12302227 | (1S)-1,2,3,4,4aalpha,7,8,8aalpha-Octahydro-1,6-dimethyl-4beta-isopropylnaphthalen-1beta-ol |
| 12302228 | (1S,4S,4aS,8aS)-1,6-dimethyl-4-propan-2-yl-3,4,4a,7,8,8a-hexahydro-2H-naphthalen-1-ol |
| 12302243 | alpha-Calacorene |
| 12303902 | 8-Isopropyl-1,3-dimethyltricyclo(4.4.0.02,7)dec-3-ene |
| 12305246 | (1aR,4aR,7R,7aS,7bS)-1,1,7-trimethyl-4-methylidene-2,3,4a,5,6,7,7a,7b-octahydro-1aH-cyclopropa[e]azulene |
| 12305247 | Alloaromadendrene |
| 12306053 | (+)-alpha-Bulgarene |
| 12308714 | Azadiradione |
| 12308716 | 17-Epiazadiradione |
| 12309449 | delta-Elemene |
| 12309491 | ent-Epiafzelechin |
| 12310089 | 5-hydroxy-3-(4-hydroxyphenyl)-7-[(2R,3R,4S,5S,6R)-3,4,5-trihydroxy-6-(hydroxymethyl)oxan-2-yl]oxychromen-4-one |
| 12312690 | Makisterone A |
| 12313023 | (+)-gamma-Bulgarene |
| 12313376 | CID 12313376 |
| 12376292 | 2-Methyl-2-pentenal, (2Z)- |
| 12443210 | 5,7-Dihydroxy-2-(3-hydroxy-4-methoxyphenyl)-3-[(2S,3R,4S,5S,6R)-3,4,5-trihydroxy-6-(hydroxymethyl)oxan-2-yl]oxychromen-4-one |
| 12677834 | 1,2,3-Trithionane |
| 13370049 | Lambertic acid |
| 13458955 | Dihydrolupeol |
| 13821181 | Anhydroaegeline |
| 13834020 | [(5S,6R,7S,8R,9R,10R,13S,17R)-17-(furan-3-yl)-6-hydroxy-4,4,8,10,13-pentamethyl-3-oxo-5,6,7,9,11,12,16,17-octahydrocyclopenta[a]phenanthren-7-yl] acetate |
| 13856086 | [17-acetyloxy-17-(2-hydroxy-5-oxo-2H-furan-3-yl)-4,4,8,10,13-pentamethyl-3,16-dioxo-5,6,7,9,11,12-hexahydrocyclopenta[a]phenanthren-7-yl] acetate |
| 13856092 | 2-(7-acetyloxy-4,4,8,10,13-pentamethyl-3,16-dioxo-6,7,9,11,12,17-hexahydro-5H-cyclopenta[a]phenanthren-17-yl)acetic acid |
| 13858079 | (1R,2S,3S,5S,8R,11R,12R)-5-(furan-3-yl)-3,11-dimethyl-12-[(2S,3R,4S,5S,6R)-3,4,5-trihydroxy-6-(hydroxymethyl)oxan-2-yl]oxy-6,14-dioxatetracyclo[10.2.2.02,11.03,8]hexadec-15-ene-7,13-dione |
| 13875741 | Nimbocinol |
| 13875755 | [6-hydroxy-17-(2-hydroxy-5-oxo-2H-furan-4-yl)-4,4,8,10,13-pentamethyl-3-oxo-5,6,7,9,11,12,16,17-octahydrocyclopenta[a]phenanthren-7-yl] acetate |
| 13875766 | [7-acetyloxy-12-hydroxy-17-(2-hydroxy-5-oxo-2H-furan-3-yl)-4,4,8,10,13-pentamethyl-3-oxo-5,6,7,9,11,12,16,17-octahydrocyclopenta[a]phenanthren-11-yl] 2-hydroxy-2-methylpropanoate |
| 13875774 | Nimbocinolide |
| 13875775 | methyl 2-[6-(2-hydroxy-5-oxo-2H-furan-4-yl)-7,9,11,15-tetramethyl-14-oxo-3,17-dioxapentacyclo[9.6.1.02,9.04,8.015,18]octadeca-7,12-dien-10-yl]acetate |
| 13965525 | 7-[[(2E,6R)-6-Hydroxy-3,7-dimethyl-2,7-octadienyl]oxy]-2H-1-benzopyran-2-one |
| 14015932 | (2R,3R,4S)-8-[(2S,4R)-7-hydroxy-2-(4-hydroxyphenyl)-3,4-dihydro-2H-chromen-4-yl]-2-(4-hydroxyphenyl)-4-[(2R,3R)-3,5,7-trihydroxy-2-(4-hydroxyphenyl)-3,4-dihydro-2H-chromen-8-yl]-3,4-dihydro-2H-chromene-3,5,7-triol |
| 14015943 | Epiafzelechin-(4beta-8)-epiafzelechin |
| 14015944 | (2S,2'S,3S,3'S,4S)-3,3',4,4'-Tetrahydro-2beta,2'beta-bis(4-hydroxyphenyl)-4alpha,8'-bi[2H-1-benzopyran]-3beta,3'beta,5,5',7,7'-hexol |
| 14015948 | Epicatechin-(4beta-8)-epiafzelechin |
| 14015949 | (2S,2'S,3S,3'S,4S)-3,3',4,4'-Tetrahydro-2beta-(3,4-dihydroxyphenyl)-2'beta-(4-hydroxyphenyl)-4alpha,8'-bi[2H-1-benzopyran]-3beta,3'beta,5,5',7,7'-hexol |
| 14015956 | (2R,3R,4S,5S,6R)-2-[[(2R,3R)-5,7-dihydroxy-2-(4-hydroxyphenyl)-3,4-dihydro-2H-chromen-3-yl]oxy]-6-(hydroxymethyl)oxane-3,4,5-triol |
| 14015959 | (2S,2'S,3S,3'S,4S)-3,3',4,4'-Tetrahydro-2'beta-(3,4-dihydroxyphenyl)-2beta-(4-hydroxyphenyl)-4alpha,8'-bi[2H-1-benzopyran]-3beta,3'beta,5,5',7,7'-hexol |
| 14015964 | (2R,3R)-8-[(2S,4R)-7-hydroxy-2-(4-hydroxyphenyl)-3,4-dihydro-2H-chromen-4-yl]-2-(4-hydroxyphenyl)-3,4-dihydro-2H-chromene-3,5,7-triol |
| 14015965 | (2R,3R)-8-[(2S,4S)-7-hydroxy-2-(4-hydroxyphenyl)-3,4-dihydro-2H-chromen-4-yl]-2-(4-hydroxyphenyl)-3,4-dihydro-2H-chromene-3,5,7-triol |
| 14015967 | (2R,3R)-6-[(2S,4R)-7-hydroxy-2-(4-hydroxyphenyl)-3,4-dihydro-2H-chromen-4-yl]-2-(4-hydroxyphenyl)-3,4-dihydro-2H-chromene-3,5,7-triol |
| 14015968 | (2R,3R)-6-[(2S,4S)-7-hydroxy-2-(4-hydroxyphenyl)-3,4-dihydro-2H-chromen-4-yl]-2-(4-hydroxyphenyl)-3,4-dihydro-2H-chromene-3,5,7-triol |
| 14136864 | Isonimbinolide |
| 14194023 | Nimbanal |
| 14194109 | Tinosporaside |
| 14213968 | 6-Formylumbelliferone |
| 14218028 | (S)-5,7-Dihydroxy-2-(4-hydroxy-3-(3-methylbut-2-en-1-yl)phenyl)chroman-4-one |
| 14287157 | 6-Ethyl-2,3,4,4a,10,10aalpha-hexahydro-7-methoxy-1,1,4abeta-trimethylphenanthrene-9(1H)-one |
| 14287159 | (4aS,10aR)-6-ethyl-7-methoxy-1,1,4a-trimethyl-4,9,10,10a-tetrahydro-3H-phenanthren-2-one |
| 14309784 | 7-(2,6,7-Trihydroxy-7-methyl-3-methylideneoctoxy)chromen-2-one |
| 14458886 | 3-Deacetylsalannin |
| 14467538 | 28-Deoxonimbolide |
| 14492795 | 5,7-Dihydroxy-4'-methoxy-8,3'-di-C-prenylflavanone |
| 14563366 | Nimbolicin |
| 14752824 | N-(4-Acetoxyphenethyl)-3-methoxy-4-acetoxy-trans-cinnamamide |
| 14807789 | 5-Methylmellein |
| 14845542 | Limocinol |
| 14845550 | Limocinin |
| 15008366 | 7-[[(2E,6R)-6-Hydroxy-7-methoxy-3,7-dimethyl-2-octenyl]oxy]-2H-1-benzopyran-2-one |
| 15108321 | Methylsyringin |
| 15215479 | Tinosponone |
| 15485379 | Epoxy nimonol |
| 15560114 | 7-[(E,6S)-6,7-dihydroxy-3,7-dimethyloct-2-enoxy]chromen-2-one |
| 15560276 | alpha-Gurjunene |
| 15560423 | Kulactone |
| 15768008 | methyl (2S,4aS,8R,10aR,10bS)-2-(furan-3-yl)-4a-hydroxy-10b-methyl-4-oxo-8-[(2R,3R,4S,5R,6R)-3,4,5-triacetyloxy-6-(acetyloxymethyl)oxan-2-yl]oxy-1,2,5,6,8,9,10,10a-octahydrobenzo[f]isochromene-7-carboxylate |
| 15768009 | methyl (2S,4aR,8R,10aR,10bS)-2-(furan-3-yl)-4a-hydroxy-10b-methyl-4-oxo-8-[(2R,3R,4S,5R,6R)-3,4,5-triacetyloxy-6-(acetyloxymethyl)oxan-2-yl]oxy-1,2,5,6,8,9,10,10a-octahydrobenzo[f]isochromene-7-carboxylate |
| 15840160 | [(5R,7R,8R,9R,10R,13R,14R,17R)-17-hydroxy-4,4,8,10,13-pentamethyl-3-oxo-5,6,7,9,11,12,14,15,16,17-decahydrocyclopenta[a]phenanthren-7-yl] (E)-3-(3-hydroxy-4-methoxyphenyl)prop-2-enoate |
| 15885442 | Meliacinin |
| 15885443 | Azadironic acid |
| 15934443 | 7-acetyl-6-hydroxy-1,1,4a-trimethyl-3,4,10,10a-tetrahydro-2H-phenanthren-9-one |
| 16126804 | dimethyl (2aR,3S,4S,4aR,5S,7aS,8R,10R,10aS,10bR)-10-acetoxy-3,5-dihydroxy-4-[(1aR,2S,3aS,6aS,7S,7aS)-6a-hydroxy-7a-methyl-3a,6a,7,7a-tetrahydro-2,7-methanofuro[2,3-b]oxireno[e]oxepin-1a(2H)-yl]-4-methyl-8-{[(2E)-2-methylbut-2-enoyl]oxy}octahydro-1H-naphtho[1,8a-c:4,5-b'c']difuran-5,10a(8H)-dicarboxylate |
| 16396350 | CID 16396350 |
| 16722121 | azadirachtin H |
| 16722130 | azadirachtin I |
| 20056138 | [(5S,6R,7S,8R,9S,10R,13S,17S)-6-methoxy-4,4,8,10,13-pentamethyl-1,12,16-trioxo-17-[(Z)-3-oxobut-1-enyl]-5,6,7,9,11,17-hexahydrocyclopenta[a]phenanthren-7-yl] acetate |
| 20488062 | Hydron;phenoxide |
| 20976991 | Heptadecyl icosanoate |
| 21581301 | [(1S,4S,5R,6S,7S,8R,11S,12R,14S,15R)-12-acetyloxy-4,7-dihydroxy-11-(hydroxymethyl)-6-[(1S,2S,6S,8S,9R,11S)-2-hydroxy-11-methyl-5,7,10-trioxatetracyclo[6.3.1.02,6.09,11]dodec-3-en-9-yl]-6-methyl-3,9-dioxatetracyclo[6.6.1.01,5.011,15]pentadecan-14-yl] (E)-2-methylbut-2-enoate |
| 21581584 | Ohchinolide B |
| 21592304 | Desfurano-6alpha-hydroxyazadiradione |
| 21594203 | Triptotriterpenic acid A |
| 21597549 | 6alpha-Hydroxy-7alpha,15beta-epoxy-4beta,8-dimethyl-1,17-dioxo-18-nor-11,12-seco-5alpha-androsta-2,13-diene-4alpha,11-dicarboxylic acid dimethyl ester |
| 21600035 | [(1S,3R,5S,7R,8R,9R,10S,13S,17S)-1,7-diacetyloxy-17-[(3S,5R,6S)-5,6-dihydroxy-7,7-dimethyloxepan-3-yl]-4,4,8,10,13-pentamethyl-2,3,5,6,7,9,11,12,16,17-decahydro-1H-cyclopenta[a]phenanthren-3-yl] benzoate |
| 21603566 | 3beta,22beta-Dihydroxyolean-12-en-29-oic acid |
| 21625636 | Borapetoside F |
| 21626436 | (2R,3R,4S)-2-(4-hydroxyphenyl)-4-[(2R,3R)-3,5,7-trihydroxy-2-(4-hydroxyphenyl)-3,4-dihydro-2H-chromen-8-yl]-8-[(2R,3R,4R)-3,5,7-trihydroxy-2-(4-hydroxyphenyl)-3,4-dihydro-2H-chromen-4-yl]-3,4-dihydro-2H-chromene-3,5,7-triol |
| 21632833 | Margocin |
| 21632843 | 19-Hydroxyferruginol |
| 21725519 | Vepaol |
| 21725521 | Deacetylazadirachtinol |
| 21725522 | 3-Acetyl-11-methoxy-1-tigloylazadirachtinin |
| 23256847 | Azadirachtol |
| 24796982 | 23-Epivepaol |
| 24867638 | 1,8-Dihydroxy-3-methyl-4a,9a-dihydroanthracene-9,10-dione |
| 24879663 | multiplolide A |
| 25769005 | (3R)-1-Oxo-3-methyl-8-hydroxy-3,4-dihydro-1H-2-benzopyran-5-carboxylic acid |
| 40469553 | (1S,2R,4S,7S,8S,11R,12R,17S,19R)-7-(furan-3-yl)-19-hydroxy-1,8,12,16,16-pentamethyl-3,6-dioxapentacyclo[9.8.0.02,4.02,8.012,17]nonadec-13-ene-5,15-dione |
| 40469561 | [(1S,2R,4S,7S,8S,11R,12R,17S,19R)-7-(furan-3-yl)-1,8,12,16,16-pentamethyl-5,15-dioxo-3,6-dioxapentacyclo[9.8.0.02,4.02,8.012,17]nonadec-13-en-19-yl] acetate |
| 42433469 | (1aR,4R,4aS,7R,7aS,7bR)-1,1,4,7-tetramethyl-2,3,4a,5,6,7,7a,7b-octahydro-1aH-cyclopropa[e]azulen-4-ol |
| 42607958 | 5,7-Dihydroxy-4'-methoxy-8-C-prenyl-3'-(3-hydroxy-3-methylbutyl)flavanone |
| 42608071 | Mundulea flavanone B |
| 42608075 | Flowerine |
| 42608116 | Flowerone |
| 42626428 | (3R)-4-methylidene-1-(propan-2-yl)bicyclo[3.1.0]hexan-3-ol |
| 44146779 | Rutaretin |
| 44259428 | Myricetin 3-rutinoside |
| 44566526 | [(1R,2R,5S,6R,10R,11S,12S,15R,16R,18S,19R)-18-acetyloxy-6-(furan-3-yl)-11-hydroxy-1,5,10,15-tetramethyl-13-oxapentacyclo[10.6.1.02,10.05,9.015,19]nonadec-8-en-16-yl] acetate |
| 44567124 | Kulinone |
| 44567142 | Azadirol |
| 44575502 | 3-O-(6-O-alpha-D-Galactopyranosyl-beta-D-galactopyranosyl)-L-glycerol |
| 44575793 | (5R,9R,10R,13S,14S,17S)-17-[(2R,3S,5R)-5-[(2S)-3,3-dimethyloxiran-2-yl]-2-hydroxyoxolan-3-yl]-4,4,10,13,14-pentamethyl-1,2,5,6,9,11,12,15,16,17-decahydrocyclopenta[a]phenanthren-3-one |
| 44579695 | Anhydromarmeline |
| 44579696 | Dehydromarmeline |
| 44579743 | aegelinoside A |
| 44579744 | aegelinoside B |
| 44583637 | Rhein methylester |
| 44584063 | dimethyl (1S,4S,5R,6S,7S,8R,11S,12R,14S,15R)-12-acetyloxy-4,7-dihydroxy-6-[(1R,2S,6S,8R,9R,11S)-2-hydroxy-11-methyl-5,7,10-trioxatetracyclo[6.3.1.02,6.09,11]dodec-3-en-9-yl]-6-methyl-14-[(E)-2-methylbut-2-enoyl]oxy-3,9-dioxatetracyclo[6.6.1.01,5.011,15]pentadecane-4,11-dicarboxylate |
| 44614139 | Protoxylocarpin G |
| 44631202 | Jziqwnppbkfopt-ablzdqehsa- |
| 44715635 | Nimbinene |
| 45103626 | 2-Isopropenyl-4-methylanthra[2,3-b]furan-5,10-dione |
| 45268397 | (2S)-5,7-dihydroxy-2-[4-methoxy-3-(3-methylbut-2-enyl)phenyl]-8-(3-methylbut-2-enyl)-2,3-dihydrochromen-4-one |
| 45272307 | 2-[(2S)-2-Hydroxypropyl]-5-methyl-7-hydroxy-4H-1-benzopyran-4-one |
| 46173826 | (1S)-1-[(2R,3R,5R)-5-hydroxy-3-[(3S,5R,9R,10R,13S,14S,17S)-3-hydroxy-4,4,10,13,14-pentamethyl-2,3,5,6,9,11,12,15,16,17-decahydro-1H-cyclopenta[a]phenanthren-17-yl]oxolan-2-yl]-2-methylpropane-1,2-diol |
| 46201020 | 1-Methyl-2-(3-methyl-2-butenyloxy)anthraquinone |
| 46211187 | (+)-9'-Isovaleroxylariciresinol |
| 46224590 | 2-O-(alpha-L-rhamnopyranosyl)-alpha-L-rhamnopyranose |
| 46919586 | Neemfruitin B |
| 49863985 | Epoxyazadiradione |
| 49864004 | Desmethyllimocin B |
| 49864005 | [(5R,7R,8R,9R,10R,13S,17S)-17-[(3S,5R)-5,6-dihydroxy-6-(2-hydroxypropan-2-yl)oxan-3-yl]-4,4,8,10,13-pentamethyl-3-oxo-5,6,7,9,11,12,16,17-octahydrocyclopenta[a]phenanthren-7-yl] acetate |
| 49864006 | Neemfruitin A |
| 51402807 | 2-(3,4-dihydroxyphenyl)-5,7-dihydroxy-3-[(2S,3R,4R,5R,6S)-3,4,5-trihydroxy-6-(hydroxymethyl)oxan-2-yl]oxychromen-4-one |
| 51694242 | (2S,4aS,10aS)-2,6-dihydroxy-7-methoxy-1,1,4a-trimethyl-3,4,10,10a-tetrahydro-2H-phenanthren-9-one |
| 52951756 | 15-Hydroxyazadiradione |
| 52951892 | 17-Hydroxyazadiradione |
| 52951893 | 7-Deacetyl-7-benzoylepoxyazadiradione |
| 52951894 | 17-epi-17-Hydroxyazadiradione |
| 52951895 | 7-Acetyl-16,17-dehydro-16-hydroxyneotrichilenone |
| 52952011 | 20,21,22,23-Tetrahydro-23-oxoazadirone |
| 52952012 | Desfuranoazadiradione |
| 52952013 | 1,3-Diacetylvilasinin |
| 52952112 | 7-Deacetyl-7-benzoylgedunin |
| 52952113 | 7-Deacetyl-17-epinimolicinol |
| 52952216 | 6-Acetylnimbandiol |
| 52952322 | Ohchinin Acetate |
| 52952323 | 2',3'-Dihydrosalannin |
| 52952435 | 17-Defurano-17-oxosalannin |
| 52952436 | Alpha-Nimolactone |
| 52952437 | Beta-Nimolactone |
| 53438729 | 1,4-Dihydroxy-6,7-dimethoxy-3-methyl-9,10-dioxo-9,10-dihydroanthracene-2-carboxylic acid |
| 54580354 | 7-Benzoylnimbocinol |
| 54580355 | [(1R,2S,5R,7S,8S,9R,12R,13R,15S,16R)-13-acetyloxy-15-hydroxy-1,5,7,12-tetramethyl-5-[(1S)-1-[(3S)-5-oxooxolan-3-yl]ethyl]-10-oxatetracyclo[7.6.1.02,7.012,16]hexadecan-8-yl] 2-methylbutanoate |
| 54581351 | dimethyl (1S,4S,5R,6S,7S,8R,11S,12R,14S,15R)-12-acetyloxy-7-hydroxy-6-[(2S,4R,6R,9R,11S)-2-hydroxy-4-methoxy-11-methyl-7,10-dioxatetracyclo[6.3.1.02,6.09,11]dodecan-9-yl]-6-methyl-14-(2-methylbutanoyloxy)-3,9-dioxatetracyclo[6.6.1.01,5.011,15]pentadecane-4,11-dicarboxylate |
| 54581352 | [(1R,2S,4R,9R,10R,11R,12S,14R,15R,18R)-14-acetyloxy-10-(2-methoxy-2-oxoethyl)-7,9,11,15-tetramethyl-6-oxo-3,17-dioxapentacyclo[9.6.1.02,9.04,8.015,18]octadec-7-en-12-yl] (Z)-2-methylbut-2-enoate |
| 54583360 | dimethyl (1S,4S,5R,6S,7S,8R,11S,12R,14S,15R)-12-acetyloxy-7-hydroxy-6-[(2S,4S,6R,9R,11S)-2-hydroxy-4-methoxy-11-methyl-7,10-dioxatetracyclo[6.3.1.02,6.09,11]dodecan-9-yl]-6-methyl-14-(2-methylbutanoyloxy)-3,9-dioxatetracyclo[6.6.1.01,5.011,15]pentadecane-4,11-dicarboxylate |
| 54583361 | [(1R,2S,4R,6R,9R,10R,11R,12S,14R,15R,18R)-6-(furan-3-yl)-14-hydroxy-10-(2-methoxy-2-oxoethyl)-7,9,11,15-tetramethyl-3,17-dioxapentacyclo[9.6.1.02,9.04,8.015,18]octadec-7-en-12-yl] (Z)-2-methylbut-2-enoate |
| 54585273 | methyl (1S,2R,3S,4R,8S,9S,10R,13R,15R)-2-acetyloxy-13-(furan-3-yl)-4-hydroxy-4,8,10,12-tetramethyl-7-oxo-16-oxatetracyclo[8.6.0.03,8.011,15]hexadeca-5,11-diene-9-carboxylate |
| 54586223 | dimethyl (1S,4S,5R,6S,7R,8R,10S,14S,16R,18S,19R,22S,23R,25S,26R)-23-acetyloxy-7,14-dihydroxy-4-methoxy-6-methyl-25-(2-methylbutanoyloxy)-3,9,11,17,20-pentaoxaoctacyclo[17.6.1.18,15.01,5.06,18.07,16.010,14.022,26]heptacos-12-ene-4,22-dicarboxylate |
| 54587249 | [(1R,2S,4R,6R,9R,10R,11R,12S,14R,15R,18R)-14-acetyloxy-6-(furan-3-yl)-10-(2-methoxy-2-oxoethyl)-7,9,11,15-tetramethyl-3,17-dioxapentacyclo[9.6.1.02,9.04,8.015,18]octadec-7-en-12-yl] (Z)-2-methylbut-2-enoate |
| 54670067 | Ascorbic Acid |
| 54758525 | [(1S,2R,3R,5R,7R,10S,11R,14R,15S)-15-[(3S,5R)-5-[(2S)-3,3-dimethyloxiran-2-yl]-2-hydroxyoxolan-3-yl]-3-hydroxy-2,6,6,10-tetramethyl-7-pentacyclo[12.3.1.01,14.02,11.05,10]octadecanyl] 3-methylbutanoate |
| 54758526 | [(1S,2R,3R,5R,7R,10S,11R,14R,15S)-15-[(3S,5R)-5-[(2S)-3,3-dimethyloxiran-2-yl]-2-hydroxyoxolan-3-yl]-3-hydroxy-2,6,6,10-tetramethyl-7-pentacyclo[12.3.1.01,14.02,11.05,10]octadecanyl] 3-methylbut-2-enoate |
| 56841069 | Nimolinone |
| 56958440 | (2S)-3alpha,2alpha-[(3R)-3-Hydroxy-1-oxabutane-1,3-diyl]-2,3-dihydro-7H-furo[3,2-g][1]benzopyran-7-one |
| 56958777 | (2S)-2alpha-(1-Hydroxy-1-methylethyl)-3alpha,9-dihydroxy-2,3-dihydro-7H-furo[3,2-g][1]benzopyran-7-one |
| 70689030 | (22R)-2beta,3beta,5,14,25-Pentahydroxy-20,22-(isopropylidenedioxy)-5beta-cholest-7-en-6-one |
| 70697879 | 3-Acetyl-7-tigloylvilasinin lactone |
| 70697889 | (5alpha,7alpha,17alpha)-4,4,8-Trimethyl-3,16-dioxo-17-(2-oxo-2,5-dihydrofuran-3-yl)androsta-1,14-dien-7-yl acetate |
| 71338636 | 1-[(Prop-1-en-1-yl)disulfanyl]butane |
| 71413104 | 1,8-Dihydroxy-3-(hydroxymethyl)-6-methylanthracene-9,10-dione |
| 71584574 | R-(+)-Marmin-6'-octanoate |
| 71584688 | R-(+)-Marmin-6'-undecanoate |
| 71584689 | R-(+)-Marmin-6'-palmitate |
| 71584690 | R-(+)-Marmin-6'-linoleate |
| 71584691 | R-(+)-Marmin-6'-cis-vaccenoate |
| 71717738 | 7-[[(2E,6R)-7-Chloro-6-hydroxy-3,7-dimethyl-2-octen-1-yl]oxy]-2H-1-benzopyran-2-one |
| 71720036 | Isophellodenol C |
| 72738894 | [4-[2-[3-(4-Acetyloxy-3-methoxyphenyl)prop-2-enoylamino]ethyl]phenyl] acetate |
| 73076982 | [15-(Furan-3-yl)-15-hydroxy-2,7,7,11,16-pentamethyl-6,14-dioxo-4-oxapentacyclo[9.7.0.02,8.03,5.012,16]octadec-12-en-10-yl] acetate |
| 73187989 | N-[2-(4-methoxyphenyl)ethenyl]-3-phenylprop-2-enamide |
| 73356511 | Nimonol |
| 73797339 | [1,7-diacetyloxy-17-(5,6-dihydroxy-7,7-dimethyloxepan-3-yl)-4,4,8,10,13-pentamethyl-2,3,5,6,7,9,11,12,16,17-decahydro-1H-cyclopenta[a]phenanthren-3-yl] benzoate |
| 73804953 | [12-Acetyloxy-4,7-dihydroxy-11-(hydroxymethyl)-6-(2-hydroxy-11-methyl-5,7,10-trioxatetracyclo[6.3.1.02,6.09,11]dodec-3-en-9-yl)-6-methyl-3,9-dioxatetracyclo[6.6.1.01,5.011,15]pentadecan-14-yl] 2-methylbut-2-enoate |
| 73813111 | [6-hydroxy-17-(2-methoxy-5-oxo-2H-furan-4-yl)-4,4,8,10,13-pentamethyl-3-oxo-5,6,7,9,11,12,16,17-octahydrocyclopenta[a]phenanthren-7-yl] 3-methylbut-2-enoate |
| 73824950 | Dimethyl 12-acetyloxy-4,7-dihydroxy-6-(2-hydroxy-4-methoxy-11-methyl-5,7,10-trioxatetracyclo[6.3.1.02,6.09,11]dodecan-9-yl)-6-methyl-14-(2-methylbut-2-enoyloxy)-3,9-dioxatetracyclo[6.6.1.01,5.011,15]pentadecane-4,11-dicarboxylate |
| 73824951 | Dimethyl 4,7-dihydroxy-6-(2-hydroxy-11-methyl-5,7,10-trioxatetracyclo[6.3.1.02,6.09,11]dodec-3-en-9-yl)-6-methyl-14-(2-methylbut-2-enoyloxy)-12-(3-phenylprop-2-enoyloxy)-3,9-dioxatetracyclo[6.6.1.01,5.011,15]pentadecane-4,11-dicarboxylate |
| 73824953 | Dimethyl 23-acetyloxy-7,14-dihydroxy-4-methoxy-6,16-dimethyl-25-(2-methylbut-2-enoyloxy)-3,9,11,17,20-pentaoxaoctacyclo[17.6.1.18,15.01,5.06,18.07,16.010,14.022,26]heptacos-12-ene-4,22-dicarboxylate |
| 74073445 | 4,5-Dihydroxy-2-methyl-2,3,4,5-tetrahydrooxecin-10-one |
| 74075981 | 1-[4-(3-hydroxy-4,4,10,13,14-pentamethyl-2,3,5,6,9,11,12,15,16,17-decahydro-1H-cyclopenta[a]phenanthren-17-yl)-5-methoxyoxolan-2-yl]-2-methylpropane-1,2-diol |
| 74336648 | (7-Hydroxy-2-methyl-5,10-dioxooxecan-4-yl) acetate |
| 74978378 | 2-(3,4-Dihydroxyphenyl)-5-hydroxy-7-methoxy-3-[3,4,5-trihydroxy-6-[[3,4,5-trihydroxy-6-(hydroxymethyl)oxan-2-yl]oxymethyl]oxan-2-yl]oxychromen-4-one |
| 75050399 | N-[2-[4-(3-methylbut-2-enoxy)phenyl]ethenyl]-3-phenylprop-2-enamide |
| 75050400 | N-[2-[4-(3-methylbut-2-enoxy)phenyl]ethyl]-3-phenylprop-2-enamide |
| 75111036 | Cordifolioside A |
| 75971805 | [6-(Furan-3-yl)-1,7,11,15,15-pentamethyl-5,14-dioxo-3-oxapentacyclo[8.8.0.02,4.02,7.011,16]octadec-12-en-18-yl] benzoate |
| 75971891 | [7-(Furan-3-yl)-1,8,12,16,16-pentamethyl-5,15-dioxo-3,6-dioxapentacyclo[9.8.0.02,4.02,8.012,17]nonadec-13-en-19-yl] benzoate |
| 75972015 | [14-Acetyloxy-10-(2-methoxy-2-oxoethyl)-7,9,11,15-tetramethyl-6-oxo-3,17-dioxapentacyclo[9.6.1.02,9.04,8.015,18]octadec-7-en-12-yl] 2-methylbut-2-enoate |
| 76152128 | [13-Acetyloxy-15-hydroxy-1,5,7,12-tetramethyl-5-[1-(5-oxooxolan-3-yl)ethyl]-10-oxatetracyclo[7.6.1.02,7.012,16]hexadecan-8-yl] 2-methylbutanoate |
| 76153908 | Methyl 2-acetyloxy-13-(furan-3-yl)-4-hydroxy-4,8,10,12-tetramethyl-7-oxo-16-oxatetracyclo[8.6.0.03,8.011,15]hexadeca-5,11-diene-9-carboxylate |
| 76311433 | CID 76311433 |
| 76316558 | Isomeldenin |
| 76316561 | Isosalanninolide |
| 76317961 | [(5R,6R,7S,8R,9R,10R,13S,17R)-17-(furan-3-yl)-6-hydroxy-4,4,8,10,13-pentamethyl-3-oxo-1,2,5,6,7,9,11,12,16,17-decahydrocyclopenta[a]phenanthren-7-yl] acetate |
| 76327056 | 11beta-Azadirachtin H |
| 76685092 | [(5R,7R,8R,9R,10R,13S,17R)-17-[(2R)-2-hydroxy-5-oxo-2H-furan-4-yl]-4,4,8,10,13-pentamethyl-3-oxo-5,6,7,9,11,12,16,17-octahydrocyclopenta[a]phenanthren-7-yl] acetate |
| 77916018 | [16-Acetyloxy-18-hydroxy-1,5,10,15-tetramethyl-7-oxo-6-(5-oxooxolan-3-yl)-13-oxapentacyclo[10.6.1.02,10.05,9.015,19]nonadec-8-en-11-yl] 2-methylbut-2-enoate |
| 78148404 | [6-(Furan-3-yl)-14-hydroxy-10-(2-methoxy-2-oxoethyl)-7,9,11,15-tetramethyl-3,17-dioxapentacyclo[9.6.1.02,9.04,8.015,18]octadec-7-en-12-yl] benzoate |
| 78157935 | 6-(4-Hydroxy-3-methyl-2-butenyl)-7-hydroxy coumarin |
| 78167002 | 5-(1,2-dihydroxy-2-methylpropyl)-3-(3-hydroxy-4,4,10,13,14-pentamethyl-2,3,5,6,9,11,12,15,16,17-decahydro-1H-cyclopenta[a]phenanthren-17-yl)oxolan-2-one |
| 85302768 | [17-(2,5-dihydroxy-2,5-dihydrofuran-3-yl)-11,12-dihydroxy-6-methoxy-4,4,8,10,13-pentamethyl-1,16-dioxo-6,7,9,11,12,17-hexahydro-5H-cyclopenta[a]phenanthren-7-yl] 3-methylbut-2-enoate |
| 85596052 | Heptacosyl eicosanoate |
| 85776164 | Eicosatrienoic acid methylester |
| 90470576 | Fistacacidin |
| 91884898 | CID 91884898 |
| 91886694 | 2',3'-Dehydrosalannol |
| 100926540 | Tinocordifolin |
| 100926541 | Tinocordifolioside |
| 100996181 | 26-Hydroxy-1,3-friedelanedione |
| 101026859 | Meliacinol |
| 101034965 | (4aS,10aS)-6-hydroxy-1,1,4a-trimethyl-7-propan-2-yl-3,4,10,10a-tetrahydrophenanthrene-2,9-dione |
| 101153492 | Meliacinolactol |
| 101153494 | Limocin D |
| 101280240 | Acetyltrichilenone |
| 101289833 | Meldenin |
| 101355584 | Meliacinanhydride |
| 101529198 | Margocilin |
| 101570727 | 6-[(2E)-3-Methyl-4-acetoxy-2-butenyl]-7-hydroxy-2H-1-benzopyran-2-one |
| 101602319 | Limocinone |
| 101602320 | Limocin A |
| 101602321 | Limocin B |
| 101634707 | methyl 2-[(1S,2R,3S,4R,8R,9S,10R,13R,15S)-13-(furan-3-yl)-2,4-dihydroxy-4,8,10,12-tetramethyl-7-oxo-16-oxatetracyclo[8.6.0.03,8.011,15]hexadeca-5,11-dien-9-yl]acetate |
| 101676207 | Cordifoliside B |
| 101676208 | Cordifoliside C |
| 101676711 | Cordifoliside A |
| 101915817 | Cordioside |
| 101916313 | Tinocordioside |
| 101919043 | dimethyl (1S,5R,6S,7S,8R,11S,12R,14R,15R)-7,14-dihydroxy-6-[(1S,2S,6S,8S,9R,11S)-3-hydroxy-11-methyl-5,7,10-trioxatetracyclo[6.3.1.02,6.09,11]dodec-3-en-9-yl]-6-methyl-12-[(E)-2-methylbut-2-enoyl]oxy-3,9-dioxatetracyclo[6.6.1.01,5.011,15]pentadecane-4,11-dicarboxylate |
| 101936072 | methyl 2-[(1R,2S,4S,6R,9R,10S,11R,15R,18S)-6-(furan-3-yl)-7,9,11,15-tetramethyl-12-oxo-3,17-dioxapentacyclo[9.6.1.02,9.04,8.015,18]octadeca-7,13-dien-10-yl]acetate |
| 101999884 | 11-Hydroxyazadirachtin B |
| 102034873 | Shahidine |
| 102063005 | methyl (1S,4S,5R,6S,7S,8R,11R,12R,14S,15S)-12-acetyloxy-4,7-dihydroxy-6-[(1S,2S,6S,8S,9R,11S)-2-hydroxy-11-methyl-5,7,10-trioxatetracyclo[6.3.1.02,6.09,11]dodec-3-en-9-yl]-6,11-dimethyl-14-[(E)-2-methylbut-2-enoyl]oxy-3,9-dioxatetracyclo[6.6.1.01,5.011,15]pentadecane-4-carboxylate |
| 102149247 | [(1R,2S,4R,6R,9R,10R,11R,12S,14R,15R,18R)-14-acetyloxy-10-(2-methoxy-2-oxoethyl)-7,9,11,15-tetramethyl-6-(5-oxo-1,2-dihydropyrrol-4-yl)-3,17-dioxapentacyclo[9.6.1.02,9.04,8.015,18]octadec-7-en-12-yl] (E)-2-methylbut-2-enoate |
| 102285347 | 6-Deacetylnimbinene |
| 102316534 | Azadirolic acid |
| 102316535 | Azadiradionol |
| 118855989 | palmatosideA |
| 123981968 | 6-(4-Acetoxy-3-methyl-2-butenyl)-7-hydroxycoumarin |
| 124305339 | (1aR,4R,4aR,7R,7aR,7bS)-1,1,4,7-tetramethyl-2,3,4a,5,6,7,7a,7b-octahydro-1aH-cyclopropa[e]azulen-4-ol |
| 124629574 | methyl 2-[(1R,2S,4S,6R,9R,10S,11R,15R,18S)-6-(furan-3-yl)-7,9,11,15-tetramethyl-12,16-dioxo-3,17-dioxapentacyclo[9.6.1.02,9.04,8.015,18]octadeca-7,13-dien-10-yl]acetate |
| 129010007 | (2S)-5-hydroxy-2-(3-hydroxy-4-methoxyphenyl)-7-[(2S,3R,4S,5S,6R)-3,4,5-trihydroxy-6-[[(2R,3R,4S,5R,6S)-3,4,5-trihydroxy-6-methyloxan-2-yl]oxymethyl]oxan-2-yl]oxy-2,3-dihydrochromen-4-one |
| 129712290 | (E)-4-[[(E)-hex-4-en-3-yl]disulfanyl]hex-2-ene |
| 131676058 | [(1R,2S,6R,9R,10R,11R,12S,14R,15R)-14-acetyloxy-6-(furan-3-yl)-10-(2-methoxy-2-oxoethyl)-7,9,11,15-tetramethyl-3,17-dioxapentacyclo[9.6.1.02,9.04,8.015,18]octadec-7-en-12-yl] (E)-2-methylbut-2-enoate |
| 131698851 | dimethyl (1S,4S,5R,6S,7S,11S,12R,14S,15R)-12-acetyloxy-4,7-dihydroxy-6-[(1S,2S,6S,9R,11S)-2-hydroxy-11-methyl-5,7,10-trioxatetracyclo[6.3.1.02,6.09,11]dodec-3-en-9-yl]-6-methyl-14-[(E)-2-methylbut-2-enoyl]oxy-3,9-dioxatetracyclo[6.6.1.01,5.011,15]pentadecane-4,11-dicarboxylate |
| 131705161 | (1aR,4S,4aS,7R,7aR,7bS)-1,1,4,7-tetramethyl-2,3,4a,5,6,7,7a,7b-octahydro-1aH-cyclopropa[e]azulen-4-ol |
| 131875206 | [(5R,7R,8R,9R,10R,13S,14R,17R)-17-(furan-3-yl)-4,4,8,10,13-pentamethyl-3,15-dioxo-6,7,9,11,12,14,16,17-octahydro-5H-cyclopenta[a]phenanthren-7-yl] acetate |
| 132990894 | Gal(a1-6)D-Ido(a1-6)Glc(a1-2b)Psif |
| 135369651 | Hexadecanoate;hydron |
| 136360357 | 5-Hydroxyl methyl furfural |
| 138115244 | methyl 2-[(1R,2S,4R,9R,11R,15R,18R)-6-(furan-3-yl)-7,9,11,15-tetramethyl-12,16-dioxo-3,17-dioxapentacyclo[9.6.1.02,9.04,8.015,18]octadeca-7,13-dien-10-yl]acetate |
| 139057051 | Hydron;octadecanoate |
| 139057501 | [(5S,6S,7R,8S,9S,10S,13R,17S)-17-(furan-3-yl)-6-hydroxy-4,4,8,10,13-pentamethyl-3-oxo-5,6,7,9,11,12,16,17-octahydrocyclopenta[a]phenanthren-7-yl] acetate |
| 152743364 | 1,3,3-trimethyl-2-[(1E,3E,5E,7E,9E)-3,7,12,16-tetramethyl-18-(2,6,6-trimethylcyclohexen-1-yl)octadeca-1,3,5,7,9,11,13,15,17-nonaenyl]cyclohexene |
| 154496877 | (1aR,7S,7aS,7bS)-1,1,4,7-tetramethyl-1a,2,3,5,6,7,7a,7b-octahydrocyclopropa[e]azulene |
| 154497094 | methyl 2-[(1S,2S,4S,6S,9R,10R,11R,15S,18R)-6-(furan-3-yl)-7,9,11,15-tetramethyl-12,16-dioxo-3,17-dioxapentacyclo[9.6.1.02,9.04,8.015,18]octadeca-7,13-dien-10-yl]acetate |
| 154497120 | (5R,9R,10R,13S,14S,17S)-17-[(1R)-1-[(2R,3S,4S)-3,4-dihydroxy-5,5-dimethyloxolan-2-yl]ethyl]-4,4,10,13,14-pentamethyl-1,2,5,6,9,11,12,15,16,17-decahydrocyclopenta[a]phenanthren-3-one |
| 154497153 | (1aR,4S,4aS,7S,7aS,7bR)-1,1,4,7-tetramethyl-2,3,4,5,6,7,7a,7b-octahydro-1aH-cyclopropa[h]azulen-4a-ol |
| 154497731 | (1R,4S,5S,7S)-4,10,11,11-tetramethyltricyclo[5.3.1.01,5]undec-9-ene |
| 162819794 | [(E,3R)-2-hydroxy-2,6-dimethyl-8-(2-oxochromen-7-yl)oxyoct-6-en-3-yl] octadec-11-enoate |
| 162842140 | (4aS,10aR)-6-hydroxy-7-[(2S)-1-hydroxypropan-2-yl]-1,1,4a-trimethyl-3,4,10,10a-tetrahydrophenanthrene-2,9-dione |
| 162845059 | (6Z,9Z)-12-[(1S,2S)-2-pentylcyclopropyl]dodeca-6,9-dienoic acid |
| 162845288 | 9-(3-Methyl-2-oxobut-3-enoxy)-2,3-dihydrofuro[3,2-g]chromen-7-one |
| 162847066 | 7-[(6R)-7-chloro-6-hydroxy-3,7-dimethyloct-2-enoxy]chromen-2-one |
| 162847297 | (1S,2R,4S,8aR)-1-[(2S)-2-(furan-3-yl)-2-hydroxyethyl]-2-hydroxy-5-methoxycarbonyl-1-methyl-4-[(2R,3R,4S,5R,6R)-3,4,5-trihydroxy-6-(hydroxymethyl)oxan-2-yl]oxy-3,4,6,7,8,8a-hexahydronaphthalene-2-carboxylic acid |
| 162847298 | (1S,2R,4S,8aR)-1-[(2S)-2-(furan-3-yl)-2-hydroxyethyl]-2-hydroxy-5-methoxycarbonyl-1-methyl-4-[(2R,3R,4S,5S,6R)-3,4,5-trihydroxy-6-(hydroxymethyl)oxan-2-yl]oxy-3,4,6,7,8,8a-hexahydronaphthalene-2-carboxylic acid |
| 162853040 | [(1R,2S,4R,6R,9R,10R,11R,12S,14R,15R,18R)-6-(furan-3-yl)-14-hydroxy-10-(2-methoxy-2-oxoethyl)-7,9,11,15-tetramethyl-3,17-dioxapentacyclo[9.6.1.02,9.04,8.015,18]octadec-7-en-12-yl] 2-methylbut-2-enoate |
| 162853682 | [14-acetyloxy-6-(2-hydroxy-5-oxo-2H-furan-4-yl)-10-(2-methoxy-2-oxoethyl)-7,9,11,15-tetramethyl-3,17-dioxapentacyclo[9.6.1.02,9.04,8.015,18]octadec-7-en-12-yl] 2-methylbut-2-enoate |
| 162853683 | [(1R,2S,4R,6R,9R,10R,11R,12S,14R,15R,18R)-14-acetyloxy-6-[(2S)-2-hydroxy-5-oxo-2H-furan-4-yl]-10-(2-methoxy-2-oxoethyl)-7,9,11,15-tetramethyl-3,17-dioxapentacyclo[9.6.1.02,9.04,8.015,18]octadec-7-en-12-yl] (E)-2-methylbut-2-enoate |
| 162857811 | N-[(2R)-2-hydroxy-2-[4-[(2S)-2-hydroxy-3-methylbut-3-enoxy]phenyl]ethyl]-3-phenylprop-2-enamide |
| 162859638 | 10-Hydroxy-7-(4-hydroxy-4-methylpent-2-enyl)-2,9,13,17,17-pentamethyl-5-oxapentacyclo[10.8.0.02,9.04,8.013,18]icos-1(20)-ene-6,16-dione |
| 162867112 | 9-[(2S)-2-hydroxy-3-methylbut-3-enoxy]-2,3-dihydrofuro[3,2-g]chromen-7-one |
| 162867118 | 7-hydroxy-6-[(2R)-2-hydroxy-3-(hydroxymethyl)but-3-enyl]chromen-2-one |
| 162867119 | 9-(3-Methylbut-2-enoxy)-2,3-dihydrofuro[3,2-g]chromen-7-one |
| 162867134 | (1S,4aR,4bS,7S,10aR)-1,4a,7-trimethyl-7-(2-sulfooxyethyl)-3,4,4b,5,6,8,10,10a-octahydro-2H-phenanthrene-1-carboxylic acid |
| 162867348 | N-[(2S)-2-(4-methoxyphenyl)-2-[(2R,3R,4S,5S,6R)-3,4,5-trihydroxy-6-(hydroxymethyl)oxan-2-yl]oxyethyl]-3-phenylprop-2-enamide |
| 162874247 | [(5R,7R,8R,9R,10R,13S,17S)-17-[(1S,4S,6R)-1-(2-hydroxypropan-2-yl)-2,7-dioxabicyclo[4.1.0]heptan-4-yl]-4,4,8,10,13-pentamethyl-3-oxo-5,6,7,9,11,12,16,17-octahydrocyclopenta[a]phenanthren-7-yl] acetate |
| 162874358 | [(1R,2R,5S,6S,10R,11S,12R,15R,16R,18S,19R)-16-acetyloxy-11-hydroxy-1,5,10,15-tetramethyl-6-[(3S)-oxolan-3-yl]-13-oxapentacyclo[10.6.1.02,10.05,9.015,19]nonadec-8-en-18-yl] 3-methylbut-2-enoate |
| 162876410 | methyl (1S,4R,5R,6R,7R,8R,11R,12S,14R,15S)-12-acetyloxy-4,7-dihydroxy-6-[(1S,2S,6S,8S,9R,11S)-2-hydroxy-11-methyl-5,7,10-trioxatetracyclo[6.3.1.02,6.09,11]dodec-3-en-9-yl]-6-methyl-14-[(E)-2-methylbut-2-enoyl]oxy-3,9-dioxatetracyclo[6.6.1.01,5.011,15]pentadecane-11-carboxylate |
| 162876411 | methyl (1S,4S,5R,6S,7S,8R,11S,12R,14S,15R)-12-acetyloxy-4,7-dihydroxy-6-[(1S,2R,6S,8S,9R,11S)-2-hydroxy-11-methyl-5,7,10-trioxatetracyclo[6.3.1.02,6.09,11]dodec-3-en-9-yl]-6-methyl-14-[(E)-2-methylbut-2-enoyl]oxy-3,9-dioxatetracyclo[6.6.1.01,5.011,15]pentadecane-11-carboxylate |
| 162878377 | 1,5-Dimethylcycloocta-2,4,6-trien-1-ol |
| 162878378 | (1S,2Z,4Z,6Z)-1,5-dimethylcycloocta-2,4,6-trien-1-ol |
| 162880289 | [(5R,7R,8R,9R,10R,13S,17R)-17-(furan-3-yl)-4,4,8,10,13-pentamethyl-3-oxo-5,6,7,9,11,12,16,17-octahydrocyclopenta[a]phenanthren-7-yl] benzoate |
| 162884805 | [(5R,7R,8R,9R,10R,13S,17R)-17-[(2S)-2-methoxy-5-oxo-2H-furan-4-yl]-4,4,8,10,13-pentamethyl-3-oxo-5,6,7,9,11,12,16,17-octahydrocyclopenta[a]phenanthren-7-yl] acetate |
| 162885142 | (2R)-10-[(1S,2R)-2-hexylcyclopropyl]-2-hydroxydecanoic acid |
| 162885807 | [(2S,3R,5R,9R,10R,13R,14S,17S)-2,14-diacetyloxy-10,13-dimethyl-6-oxo-17-[(2R,3R,5R)-2,3,6-triacetyloxy-5-ethyl-6-methylheptan-2-yl]-2,3,4,5,9,11,12,15,16,17-decahydro-1H-cyclopenta[a]phenanthren-3-yl] acetate |
| 162888038 | (5R,9R,10R,13S,14S,17S)-17-[(1R)-1-[(2S,3R,4S)-3,4-dihydroxy-5,5-dimethyloxolan-2-yl]ethyl]-4,4,10,13,14-pentamethyl-1,2,5,6,9,11,12,15,16,17-decahydrocyclopenta[a]phenanthren-3-one |
| 162890111 | [(1S,4S,5R,6S,7S,8R,11S,12R,14R,15S)-12-acetyloxy-4,7-dihydroxy-11-(hydroxymethyl)-6-[(1S,2R,6S,8S,9R,11S)-2-hydroxy-11-methyl-5,7,10-trioxatetracyclo[6.3.1.02,6.09,11]dodec-3-en-9-yl]-6-methyl-3,9-dioxatetracyclo[6.6.1.01,5.011,15]pentadecan-14-yl] (E)-2-methylbut-2-enoate |
| 162893251 | methyl 2-[(1S,2R,3R,8R,9S,10R,13R,15R)-13-(furan-3-yl)-2-hydroxy-4,8,10,12-tetramethyl-7-oxo-16-oxatetracyclo[8.6.0.03,8.011,15]hexadeca-4,11-dien-9-yl]acetate |
| 162895380 | (5R,10S,13S,14S,17S)-4,4,10,13,14-pentamethyl-17-[(2S)-6-methylhept-5-en-2-yl]-1,2,3,5,6,7,11,12,15,17-decahydrocyclopenta[a]phenanthren-16-one |
| 162895538 | (1S,4R,6R,7R,8E,10S)-6,7-dihydroxy-4-methyl-3,11-dioxabicyclo[8.1.0]undec-8-en-2-one |
| 162897369 | [(1S,2R,4R,6S,7S,10R,11R,16S,17R,18S)-6-(furan-3-yl)-17-hydroxy-1,7,11,15,15-pentamethyl-14-oxo-3-oxapentacyclo[8.8.0.02,4.02,7.011,16]octadec-12-en-18-yl] acetate |
| 162898546 | 15-[5-(1,2-Dihydroxy-2-methylpropyl)-2-methoxyoxolan-3-yl]-6,6,11,14,18-pentamethyl-2-oxapentacyclo[9.7.0.01,3.05,10.014,18]octadec-5(10)-en-7-one |
| 162899427 | 1-[(6aR)-2-[(2S,3R,4S,5S,6R)-4,5-dihydroxy-6-(hydroxymethyl)-3-[(2S,3R,4S,5S,6R)-3,4,5-trihydroxy-6-(hydroxymethyl)oxan-2-yl]oxyoxan-2-yl]oxy-1-methoxy-5,6,6a,7-tetrahydro-4H-dibenzo[de,g]quinolin-6-yl]ethanone |
| 162900804 | [(1S,2R,4S,6S,7S,10R,11R,12S,16R,18R)-6-(furan-3-yl)-12-methoxy-1,7,11,15,15-pentamethyl-5,14-dioxo-3-oxapentacyclo[8.8.0.02,4.02,7.011,16]octadecan-18-yl] acetate |
| 162902728 | methyl 2-[(1S,2R,4R,8R,9S,10R,13R)-2-acetyloxy-13-(furan-3-yl)-4-hydroxy-4,8,10,12-tetramethyl-7-oxo-16-oxatetracyclo[8.6.0.03,8.011,15]hexadeca-5,11-dien-9-yl]acetate |
| 162903500 | [(1R,2R,3R,4S)-2-[2-(furan-3-yl)ethyl]-2,4-dimethyl-3-pentylcyclohexyl]methanol |
| 162905858 | [(5R,6R,7S,8R,9R,10R,13S,17R)-6-hydroxy-17-[(2R)-2-hydroxy-5-oxo-2H-furan-4-yl]-4,4,8,10,13-pentamethyl-3-oxo-5,6,7,9,11,12,16,17-octahydrocyclopenta[a]phenanthren-7-yl] acetate |
| 162910735 | methyl (2S,6R,6aR,9R,10aS,10bS)-2-(furan-3-yl)-9-hydroxy-6a,10b-dimethyl-4-oxo-6-[(2R,3R,4S,5S,6R)-3,4,5-trihydroxy-6-(hydroxymethyl)oxan-2-yl]oxy-1,2,6,9,10,10a-hexahydrobenzo[f]isochromene-7-carboxylate |
| 162910955 | [(5R,6R,7S,8R,9R,10R,13S,17R)-6-hydroxy-17-[(2S)-2-methoxy-5-oxo-2H-furan-4-yl]-4,4,8,10,13-pentamethyl-3-oxo-5,6,7,9,11,12,16,17-octahydrocyclopenta[a]phenanthren-7-yl] 3-methylbut-2-enoate |
| 162912527 | (1aR,1bR,6aR,7R,7aS)-7-(2-hydroxypropan-2-yl)-1b,4-dimethyl-1a,5,6,6a,7,7a-hexahydroazuleno[1,2-b]oxiren-2-one |
| 162913077 | GlcA(b1-3)Gal(b1-2)a-Rha |
| 162917693 | [(2R,5R)-5-acetyloxy-4-[(5S,7R,8R,9R,10R,13S,17R)-7-hydroxy-4,4,8,10,13-pentamethyl-3-oxo-5,6,7,9,11,12,16,17-octahydrocyclopenta[a]phenanthren-17-yl]-2,5-dihydrofuran-2-yl] acetate |
| 162918748 | [(1S,2R,4S,6S,7S,10R,11R,16S,18R)-6-(furan-3-yl)-1,7,11,15,15-pentamethyl-5,14-dioxo-3-oxapentacyclo[8.8.0.02,4.02,7.011,16]octadec-12-en-18-yl] acetate |
| 162920427 | dimethyl (1S,4S,5R,6S,7R,8S,10R,12S,14S,15S,16R,18S,19R,22S,23R,25S,26R)-7,12,14,25-tetrahydroxy-6,16-dimethyl-23-[(E)-2-methylbut-2-enoyl]oxy-3,9,11,17,20-pentaoxaoctacyclo[17.6.1.18,15.01,5.06,18.07,16.010,14.022,26]heptacosane-4,22-dicarboxylate |
| 162920428 | dimethyl (1S,4S,5R,6S,7R,8S,10S,12S,14S,15S,16R,18S,19R,22S,23R,25S,26R)-7,12,14,25-tetrahydroxy-6,16-dimethyl-23-[(E)-2-methylbut-2-enoyl]oxy-3,9,11,17,20-pentaoxaoctacyclo[17.6.1.18,15.01,5.06,18.07,16.010,14.022,26]heptacosane-4,22-dicarboxylate |
| 162920595 | (1R,2R,4aR,6aR,7R,10aR,10bR)-2-[(2S)-1,4-dihydroxybutan-2-yl]-1-hydroxy-6a-(hydroxymethyl)-7,10b-dimethyl-2,4a,5,6,7,10a-hexahydro-1H-benzo[f]isochromene-4,10-dione |
| 162921038 | [(5R,6R,7S,8R,9R,10R,13S,17R)-6-hydroxy-17-[(2S)-2-hydroxy-5-oxo-2H-furan-3-yl]-4,4,8,10,13-pentamethyl-3-oxo-5,6,7,9,11,12,16,17-octahydrocyclopenta[a]phenanthren-7-yl] acetate |
| 162921039 | [(5S,6R,7S,8R,9S,10R,13S,17R)-6-hydroxy-17-[(2S)-2-hydroxy-5-oxo-2H-furan-3-yl]-4,4,8,10,13-pentamethyl-3-oxo-5,6,7,9,11,12,16,17-octahydrocyclopenta[a]phenanthren-7-yl] acetate |
| 162921836 | (1S,2R,4S,4aS,5R,8aS)-1-[(2S)-2-(furan-3-yl)-2-hydroxyethyl]-2,5-dihydroxy-5-methoxycarbonyl-1,4a-dimethyl-4-[(2R,3R,4S,5R,6R)-3,4,5-trihydroxy-6-(hydroxymethyl)oxan-2-yl]oxy-3,4,6,7,8,8a-hexahydronaphthalene-2-carboxylic acid |
| 162921838 | (1S,2S,4S,4aS,5R,8aS)-1-[(2S)-2-(furan-3-yl)-2-hydroxyethyl]-2,5-dihydroxy-5-methoxycarbonyl-1,4a-dimethyl-4-[(2R,3R,4S,5S,6R)-3,4,5-trihydroxy-6-(hydroxymethyl)oxan-2-yl]oxy-3,4,6,7,8,8a-hexahydronaphthalene-2-carboxylic acid |
| 162922533 | methyl 2-[(1S,2S,4R,6R,9R,10R,11R,15R,18R)-6-[(2S)-2-hydroxy-5-oxo-2H-furan-4-yl]-7,9,11,15-tetramethyl-14-oxo-3,17-dioxapentacyclo[9.6.1.02,9.04,8.015,18]octadeca-7,12-dien-10-yl]acetate |
| 162922614 | (2S,3R,4R,5S,6S)-2-[[(2S,3S,4S)-3,4-dihydroxy-4-(hydroxymethyl)oxolan-2-yl]oxymethyl]-6-[[(1S,2R,4S,5S)-5-hydroxy-1,7,7-trimethyl-2-bicyclo[2.2.1]heptanyl]oxy]oxane-3,4,5-triol |
| 162925476 | 4-[(2R)-2-hydroxy-3-methylbut-3-enoxy]-[1,3]dioxolo[4,5-h]chromen-8-one |
| 162925999 | [(5S,6R,7S,8R,9R,10R,13S)-6-hydroxy-4,4,8,10,13-pentamethyl-3,16-dioxo-6,7,9,11,12,17-hexahydro-5H-cyclopenta[a]phenanthren-7-yl] acetate |
| 162934403 | [(5R,7R,8R,9R,10R,13S,17S)-17-[(3R,5R)-5-methoxyoxolan-3-yl]-4,4,8,10,13-pentamethyl-3-oxo-5,6,7,9,11,12,16,17-octahydrocyclopenta[a]phenanthren-7-yl] benzoate |
| 162935328 | methyl (2S,4aR,8R,10aS,10bS)-2-(furan-3-yl)-10b-methyl-4-oxo-8-[(2R,3R,4S,5R,6R)-3,4,5-triacetyloxy-6-(acetyloxymethyl)oxan-2-yl]oxy-2,4a,5,6,8,9,10,10a-octahydro-1H-benzo[f]isochromene-7-carboxylate |
| 162935329 | methyl (2R,4aR,8R,10aR,10bR)-2-(furan-3-yl)-10b-methyl-4-oxo-8-[(2R,3R,4S,5R,6R)-3,4,5-triacetyloxy-6-(acetyloxymethyl)oxan-2-yl]oxy-2,4a,5,6,8,9,10,10a-octahydro-1H-benzo[f]isochromene-7-carboxylate |
| 162938666 | [(4bR,8aS)-2,4b,8,8a-tetramethyl-10-oxo-6,9-dihydro-5H-phenanthren-3-yl] acetate |
| 162940876 | [(1S,4R,6R,7R,8E,10S)-7-hydroxy-4-methyl-2-oxo-3,11-dioxabicyclo[8.1.0]undec-8-en-6-yl] acetate |
| 162944734 | (6aR)-2-[(2S,3R,4S,5S,6R)-4,5-dihydroxy-6-(hydroxymethyl)-3-[(2S,3R,4S,5S,6R)-3,4,5-trihydroxy-6-(hydroxymethyl)oxan-2-yl]oxyoxan-2-yl]oxy-1-methoxy-5,6,6a,7-tetrahydro-4H-dibenzo[de,g]quinoline-6-carbaldehyde |
| 162946727 | (1S,2S,4S,7S,9S,12S,13S,16S)-4-(furan-3-yl)-12-hydroxy-2,16-dimethyl-13-[(2S,3R,4S,5S,6R)-3,4,5-trihydroxy-6-(hydroxymethyl)oxan-2-yl]oxy-5,10-dioxatetracyclo[7.6.1.02,7.012,16]hexadecane-6,11-dione |
| 162949507 | [(1R,2S,4R,6R,9R,10R,11R,12S,14R,15R,18R)-14-acetyloxy-10-(2-methoxy-2-oxoethyl)-7,9,11,15-tetramethyl-6-(5-oxo-1,2-dihydropyrrol-3-yl)-3,17-dioxapentacyclo[9.6.1.02,9.04,8.015,18]octadec-7-en-12-yl] (E)-2-methylbut-2-enoate |
| 162951523 | [(1R,2S,4R,6R,9S,10S,11S,12S,14R,15R,18R)-14-hydroxy-6-[(2S)-2-hydroxy-5-oxo-2H-furan-3-yl]-10-(2-methoxy-2-oxoethyl)-7,9,11,15-tetramethyl-3,17-dioxapentacyclo[9.6.1.02,9.04,8.015,18]octadec-7-en-12-yl] 3-methylbut-2-enoate |
| 162952996 | [(5S,6R,7S,8R,9R,10R,13S,17R)-17-(2,3-dihydrofuran-4-yl)-6-hydroxy-4,4,8,10,13-pentamethyl-3-oxo-5,6,7,9,11,12,16,17-octahydrocyclopenta[a]phenanthren-7-yl] acetate |
| 162953557 | 2-[(5S,7R,8S,9R,10S,13S,17S)-7-acetyloxy-4,4,8,10,13-pentamethyl-3,16-dioxo-6,7,9,11,12,17-hexahydro-5H-cyclopenta[a]phenanthren-17-yl]acetic acid |
| 162955224 | [(1R,2S,4R,6R,9R,10R,11R,14R,15R,18R)-6-[(2S)-2-(2-hydroxypropan-2-yl)-5-oxo-2H-furan-4-yl]-10-(2-methoxy-2-oxoethyl)-7,9,11,15-tetramethyl-3,17-dioxapentacyclo[9.6.1.02,9.04,8.015,18]octadec-7-en-14-yl] 3-methylbut-2-enoate |
| 162956849 | [(5R,6R,7S,8R,9R,10R,13S,17R)-17-[(2R)-2-hydroxy-5-oxo-2H-furan-3-yl]-6-methoxy-4,4,8,10,13-pentamethyl-3-oxo-5,6,7,9,11,12,16,17-octahydrocyclopenta[a]phenanthren-7-yl] acetate |
| 162957338 | N-[(1R,2S)-1-[4-(2-cyanoethoxy)phenyl]-1,3-dihydroxypropan-2-yl]formamide |
| 162963098 | 4-[[(3R,4S)-4-[(S)-hydroxy-(4-hydroxy-3-methoxyphenyl)methyl]oxolan-3-yl]methyl]-2-methoxyphenol |
| 162963358 | (1S,4aR,4bR,7R,10aR)-1,4a,7-trimethyl-7-(2-sulfooxyethyl)-3,4,4b,5,6,10a-hexahydro-2H-phenanthrene-1-carboxylic acid |
| 162963628 | methyl 2-[(1R,2S,4S,6R,9S,10S,11S,15R,18S)-6-(furan-3-yl)-7,9,11,15-tetramethyl-12,16-dioxo-3,17-dioxapentacyclo[9.6.1.02,9.04,8.015,18]octadeca-7,13-dien-10-yl]acetate |
| 162964435 | methyl 2-[(1S,2S,4R,6R,9R,10R,11R,15R,18R)-6-[(2R)-2-hydroxy-5-oxo-2H-furan-3-yl]-7,9,11,15-tetramethyl-14-oxo-3,17-dioxapentacyclo[9.6.1.02,9.04,8.015,18]octadeca-7,12-dien-10-yl]acetate |
| 162965363 | (3R,8R,9R,10S,13R,14R,17S)-17-[(2R,5R)-5-ethyl-6-methylheptan-2-yl]-10,13-dimethyl-2,3,4,7,8,9,11,12,14,15,16,17-dodecahydro-1H-cyclopenta[a]phenanthren-3-ol |
| 162968547 | [(1S,4R,6R,7R,8E,10S)-6-hydroxy-4-methyl-2-oxo-3,11-dioxabicyclo[8.1.0]undec-8-en-7-yl] acetate |
| 162970859 | methyl 2-[(1S,2R,3S,8R,9S,10R,13R,15S)-2-acetyloxy-13-(furan-3-yl)-4,8,10,12-tetramethyl-7-oxo-16-oxatetracyclo[8.6.0.03,8.011,15]hexadeca-4,11-dien-9-yl]acetate |
| 162971118 | (2R,3R,4S,5S,6S)-2-[[(3S,8S,9S,10R,13R,14S,17R)-17-[(2R,5R)-5-ethyl-6-methylheptan-2-yl]-10,13-dimethyl-2,3,4,7,8,9,11,12,14,15,16,17-dodecahydro-1H-cyclopenta[a]phenanthren-3-yl]oxy]-6-methoxyoxane-3,4,5-triol |
| 162971309 | (2R,3S,4R,5R,6S)-2-[4-[(3S,3aR,6S,6aR)-6-[3-methoxy-4-[(2R,3S,4R,5R,6S)-3,4,5-trihydroxy-6-(hydroxymethyl)oxan-2-yl]oxyphenyl]-1,3,3a,4,6,6a-hexahydrofuro[3,4-c]furan-3-yl]-2-methoxyphenoxy]-6-(hydroxymethyl)oxane-3,4,5-triol |
| 162971728 | Benzyl 2-hydroxy-5-(4-hydroxy-2,5-dimethoxy-3-phenylmethoxycarbonylphenyl)-3,6-dimethoxybenzoate |
| 162973005 | [(5R,7R,8S,9S,10S,13S,17R)-17-(furan-3-yl)-4,4,8,10,13-pentamethyl-3-oxo-5,6,7,9,11,12,16,17-octahydrocyclopenta[a]phenanthren-7-yl] acetate |
| 162973509 | methyl 2-[(1S,2R,3S,4R,8R,9S,10R,13R,15R)-13-(furan-3-yl)-2,4-dihydroxy-4,8,10,12-tetramethyl-7-oxo-16-oxatetracyclo[8.6.0.03,8.011,15]hexadeca-5,11-dien-9-yl]acetate |
| 162973510 | methyl 2-[(1S,2R,3R,4R,8R,9S,10R,13R,15S)-13-(furan-3-yl)-2,4-dihydroxy-4,8,10,12-tetramethyl-7-oxo-16-oxatetracyclo[8.6.0.03,8.011,15]hexadeca-5,11-dien-9-yl]acetate |
| 162974828 | methyl (1S,6R,7S,8S,9R,12S,13R,15S,16S)-13-acetyloxy-8-hydroxy-7-[(1S,2S,6R,8S,9R,11S)-2-hydroxy-11-methyl-5,7,10-trioxatetracyclo[6.3.1.02,6.09,11]dodec-3-en-9-yl]-7-methyl-15-[(E)-2-methylbut-2-enoyl]oxy-4,5-dioxo-3,10-dioxatetracyclo[7.6.1.01,6.012,16]hexadecane-12-carboxylate |
| 162975776 | [(1R,2R,5R,6R,11R,12S,13R,16R,17R,19S,20R)-6-(furan-3-yl)-12,17-dihydroxy-1,5,11,16-tetramethyl-8-oxo-7,14-dioxapentacyclo[11.6.1.02,11.05,10.016,20]icos-9-en-19-yl] 2,3-dimethylbut-2-enoate |
| 162975883 | methyl 2-[(1R,2S,4S,6R,9R,10S,11R,15R,18R)-6-(furan-3-yl)-7,9,11,15-tetramethyl-12-oxo-3,17-dioxapentacyclo[9.6.1.02,9.04,8.015,18]octadeca-7,13-dien-10-yl]acetate |
| 162976009 | [(1R,2S,4S,6R,9R,10R,11R,12S,14R,15R,18R)-14-acetyloxy-6-(furan-3-yl)-10-(2-methoxy-2-oxoethyl)-7,9,11,15-tetramethyl-3,17-dioxapentacyclo[9.6.1.02,9.04,8.015,18]octadec-7-en-12-yl] 3-methylbutanoate |
| 162981932 | 1-[4-(3-hydroxy-4,4,10,13,14-pentamethyl-2,3,5,6,12,15,16,17-octahydro-1H-cyclopenta[a]phenanthren-17-yl)-5-methoxyoxolan-2-yl]-2-methylpropane-1,2-diol |
| 162983832 | Gal(b1-3)[GlcA(b1-6)]Gal(b1-3)b-Gal |
| 162984585 | [(5R,7R,8R,9R,10R,13S,17S)-17-[(3S,5R,6S)-5,6-dihydroxy-6-(2-hydroxypropan-2-yl)oxan-3-yl]-4,4,8,10,13-pentamethyl-3-oxo-5,6,7,9,11,12,16,17-octahydrocyclopenta[a]phenanthren-7-yl] acetate |
| 162993729 | methyl (1S,4R,5R,6S,7S,8S,11R,12R,14S,15S)-12-acetyloxy-4,7-dihydroxy-6-[(1S,2R,6S,8S,9R,11S)-2-hydroxy-11-methyl-5,7,10-trioxatetracyclo[6.3.1.02,6.09,11]dodec-3-en-9-yl]-6,11-dimethyl-14-[(E)-2-methylbut-2-enoyl]oxy-3,9-dioxatetracyclo[6.6.1.01,5.011,15]pentadecane-4-carboxylate |
| 162995221 | [(5S,7R,8R,9R,10R,13S,17S)-17-[(3S,5R)-5-[(1S)-1,2-dihydroxy-2-methylpropyl]oxolan-3-yl]-4,4,8,10,13-pentamethyl-3-oxo-5,6,7,9,11,12,16,17-octahydrocyclopenta[a]phenanthren-7-yl] acetate |
| 162995965 | [(5S,7R,8R,9R,10R,13R,17R)-17-[(2R)-2-hydroxy-5-oxo-2H-furan-4-yl]-4,4,8,10,13-pentamethyl-3-oxo-5,6,7,9,11,12,16,17-octahydrocyclopenta[a]phenanthren-7-yl] acetate |
| 162999362 | [(1R,2S,4R,9R,10R,11R,12S,14R,15R,18R)-14-acetyloxy-10-(2-methoxy-2-oxoethyl)-7,9,11,15-tetramethyl-6-oxo-3,17-dioxapentacyclo[9.6.1.02,9.04,8.015,18]octadec-7-en-12-yl] 2-methylbut-2-enoate |
| 163003104 | [(E,3R)-2-hydroxy-2,6-dimethyl-8-(2-oxochromen-7-yl)oxyoct-6-en-3-yl] octadeca-9,12-dienoate |
| 163003554 | [(5R,7R,8R,9R,10R,13S,17S)-17-[(3R,5S)-5-hydroxyoxolan-3-yl]-4,4,8,10,13-pentamethyl-3-oxo-5,6,7,9,11,12,16,17-octahydrocyclopenta[a]phenanthren-7-yl] acetate |
| 163003555 | [(5S,7R,8R,9R,10R,13S,17S)-17-[(3R,5R)-5-hydroxyoxolan-3-yl]-4,4,8,10,13-pentamethyl-3-oxo-5,6,7,9,11,12,16,17-octahydrocyclopenta[a]phenanthren-7-yl] acetate |
| 163004540 | (5R,9R,10R,13S,14S,17R)-17-[(2R)-2-[(1R,2R)-1,3-dihydroxy-2-methylpropyl]-2,3-dihydrofuran-4-yl]-4,4,10,13,14-pentamethyl-1,2,5,6,9,11,12,15,16,17-decahydrocyclopenta[a]phenanthren-3-one |
| 163004763 | [(2R)-2,3-dihydroxypropyl] tetracosanoate |
| 163005368 | (3R,4R)-4-[4-(2-cyanoethoxy)phenyl]-3,4-dihydroxybutanenitrile |
| 163010578 | [(5S,7R,8R,9R,10R,13R,17R)-4,4,8,10,13-pentamethyl-3,16-dioxo-17-(5-oxo-2H-furan-4-yl)-6,7,9,11,12,17-hexahydro-5H-cyclopenta[a]phenanthren-7-yl] acetate |
| 163011590 | (2S)-5,7-dihydroxy-2-[4-methoxy-3-(3-methylbut-3-enyl)phenyl]-8-(3-methylbut-2-enyl)-2,3-dihydrochromen-4-one |
| 163012984 | (2S,4aS,10aS)-2,6-dihydroxy-1,1,4a-trimethyl-7-propan-2-yl-3,4,10,10a-tetrahydro-2H-phenanthren-9-one |
| 163014941 | (2S,3S,4R,5R,6S)-2-[(2R,3R)-4-hydroxy-2,3-bis[(4-hydroxy-3-methoxyphenyl)methyl]butoxy]-6-(hydroxymethyl)oxane-3,4,5-triol |
| 163015292 | GlcA(b1-3)Gal(b1-3)[GlcA(b1-6)]b-Gal |
| 163016882 | (2S,4aR,6aR,7R,10aR,10bS)-2-(furan-3-yl)-4a-hydroxy-6a,10b-dimethyl-7-[(2R,3R,4S,5S,6R)-3,4,5-trihydroxy-6-(hydroxymethyl)oxan-2-yl]oxy-2,5,6,7,10,10a-hexahydro-1H-benzo[f]isochromen-4-one |
| 163017755 | (2S)-5,7,8-trihydroxy-2-[4-hydroxy-3-(3-methylbut-3-enyl)phenyl]-2,3-dihydrochromen-4-one |
| 163018012 | 2-[(1R,2S,4R,6S,9R,10R,11R,12S,14R,15R,18R)-6-(furan-3-yl)-12,14-dihydroxy-7,9,11,15-tetramethyl-3,17-dioxapentacyclo[9.6.1.02,9.04,8.015,18]octadec-7-en-10-yl]acetic acid |
| 163018356 | (1R,2R,4S,7R,9R,12R,16R)-4-(furan-3-yl)-12-hydroxy-2,16-dimethyl-5,10-dioxatetracyclo[7.6.1.02,7.012,16]hexadec-13-ene-6,11-dione |
| 163021866 | methyl (2S,4aS,6S,10aS,10bS)-2-(furan-3-yl)-10b-methyl-4-oxo-6-[(2R,3R,4S,5S,6R)-3,4,5-trihydroxy-6-(hydroxymethyl)oxan-2-yl]oxy-2,4a,5,6,8,9,10,10a-octahydro-1H-benzo[f]isochromene-7-carboxylate |
| 163025008 | Rha(a1-2)Rha(a1-5)b-Araf |
| 163026334 | methyl (2S,4aS,6S,6aR,9S,10aS,10bS)-2-(furan-3-yl)-9-hydroxy-6a,10b-dimethyl-4-oxo-6-[(2R,3R,4S,5S,6R)-3,4,5-trihydroxy-6-(hydroxymethyl)oxan-2-yl]oxy-1,2,4a,5,6,9,10,10a-octahydrobenzo[f]isochromene-7-carboxylate |
| 163026908 | [(5R,7R,8S,9S,10R,11S,12R,13S,17R)-7-acetyloxy-12-hydroxy-17-[(2R)-2-hydroxy-5-oxo-2H-furan-3-yl]-4,4,8,10,13-pentamethyl-3-oxo-5,6,7,9,11,12,16,17-octahydrocyclopenta[a]phenanthren-11-yl] 2-hydroxy-2-methylpropanoate |
| 163028748 | [(2S,3R,5R,9R,10R,13R,14S,17S)-2,14-diacetyloxy-10,13-dimethyl-6-oxo-17-[(2R,3R)-2,3,6-triacetyloxy-6-methylheptan-2-yl]-2,3,4,5,9,11,12,15,16,17-decahydro-1H-cyclopenta[a]phenanthren-3-yl] acetate |
| 163029829 | [(1R,2S,5S,6S,10R,11S,12R,15R,16R,18S,19R)-16-acetyloxy-11-hydroxy-1,5,10,15-tetramethyl-6-[(3S)-5-oxooxolan-3-yl]-13-oxapentacyclo[10.6.1.02,10.05,9.015,19]nonadec-8-en-18-yl] 3-methylbut-2-enoate |
| 163030723 | [(5S,6R,7S,8R,9R,10R,13S,17R)-17-(furan-3-yl)-7-hydroxy-4,4,8,10,13-pentamethyl-3-oxo-5,6,7,9,11,12,16,17-octahydrocyclopenta[a]phenanthren-6-yl] acetate |
| 163030830 | [(1S,2S,3S,4R,5R,7R,8R,9S,10S,13R,15S)-2-acetyloxy-4-formyl-13-(furan-3-yl)-5-hydroxy-9-(2-methoxy-2-oxoethyl)-4,8,10,12-tetramethyl-16-oxatetracyclo[8.6.0.03,8.011,15]hexadec-11-en-7-yl] (E)-2-methylbut-2-enoate |
| 163033851 | methyl 2-[(1R,4R,5R,7S,8S,9R,10S,11S,12S)-5,7-diacetyloxy-11-hydroxy-10-[(1S,2S,6S,8S,9R,11S)-2-hydroxy-11-methyl-5,7,10-trioxatetracyclo[6.3.1.02,6.09,11]dodec-3-en-9-yl]-4,8,10-trimethyl-2-oxatricyclo[6.3.1.04,12]dodecan-9-yl]-2-oxoacetate |
| 163036215 | [(5R,6R,7S,8R,9R,10R,13S,17R)-17-(furan-2-yl)-7-hydroxy-4,4,8,10,13-pentamethyl-3-oxo-5,6,7,9,11,12,16,17-octahydrocyclopenta[a]phenanthren-6-yl] acetate |
| 163040884 | dimethyl (1S,4S,5S,6R,7S,8R,11S,12R,14S,15R)-12-acetyloxy-4,7-dihydroxy-6-[(1R,2R,6S,8S)-2-hydroxy-10-methyl-5,7-dioxatricyclo[6.2.1.02,6]undeca-3,9-dien-9-yl]-6-methyl-14-[(E)-2-methylbut-2-enoyl]oxy-3,9-dioxatetracyclo[6.6.1.01,5.011,15]pentadecane-4,11-dicarboxylate |
| 163042119 | [(5R,7R,8S,9S,10S,13R,17S)-17-acetyloxy-17-[(2R)-2-hydroxy-5-oxo-2H-furan-3-yl]-4,4,8,10,13-pentamethyl-3,16-dioxo-5,6,7,9,11,12-hexahydrocyclopenta[a]phenanthren-7-yl] acetate |
| 163042754 | (2R,3R,4S,5S,6R)-2-[[(3S,8R,9R,10R,13R,14R,17R)-17-[(2R,5R)-5-ethyl-6-methylheptan-2-yl]-10,13-dimethyl-2,3,4,7,8,9,11,12,14,15,16,17-dodecahydro-1H-cyclopenta[a]phenanthren-3-yl]oxy]-6-(hydroxymethyl)oxane-3,4,5-triol |
| 163045194 | (2S)-5,7-dihydroxy-2-[4-hydroxy-3-(3-methylbut-3-enyl)phenyl]-2,3-dihydrochromen-4-one |
| 163046290 | [(1R,2S,4R,6R,9R,10R,11R,12S,14R,15R,18R)-6-(furan-3-yl)-14-hydroxy-10-(2-methoxy-2-oxoethyl)-7,9,11,15-tetramethyl-3,17-dioxapentacyclo[9.6.1.02,9.04,8.015,18]octadec-7-en-12-yl] 2,3-dimethylbut-2-enoate |
| 163046350 | (4bR,8aS)-4b,8,8-trimethyl-2-propan-2-yl-5,6,7,8a,9,10-hexahydrophenanthrene-1,4-diol |
| 163047849 | [(5S,6R,7S,8R,9S,10R,11S,12R,13S,17R)-17-[(2R,5S)-2,5-dihydroxy-2,5-dihydrofuran-3-yl]-11,12-dihydroxy-6-methoxy-4,4,8,10,13-pentamethyl-1,16-dioxo-6,7,9,11,12,17-hexahydro-5H-cyclopenta[a]phenanthren-7-yl] 3-methylbut-2-enoate |
| 163049962 | [(1S,2R,7R,8S,9R,10S,11R,15R,16S)-15-(furan-3-yl)-7,9-dihydroxy-2,7,11,16-tetramethyl-5-oxo-10-tetracyclo[9.7.0.02,8.012,16]octadeca-3,12-dienyl] acetate |
| 163057991 | (Z)-5-[(5S,6S,7S,8R,9R,10R,13S,17S)-6-acetyloxy-7-hydroxy-4,4,8,10,13-pentamethyl-3-oxo-5,6,7,9,11,12,16,17-octahydrocyclopenta[a]phenanthren-17-yl]hex-4-enoic acid |
| 163059685 | 7-[(6R)-6-hydroxy-3,7-dimethylocta-2,7-dienoxy]chromen-2-one |
| 163063327 | [(5R,7R,8R,9R,10R,13S,17R)-17-[(2S)-2-hydroxy-5-oxo-2H-furan-3-yl]-4,4,8,10,13-pentamethyl-3-oxo-5,6,7,9,11,12,16,17-octahydrocyclopenta[a]phenanthren-7-yl] acetate |
| 163063328 | [(5S,7R,8R,9R,10R,13R,17R)-17-[(2S)-2-hydroxy-5-oxo-2H-furan-3-yl]-4,4,8,10,13-pentamethyl-3-oxo-5,6,7,9,11,12,16,17-octahydrocyclopenta[a]phenanthren-7-yl] acetate |
| 163067369 | methyl (2S,4aR,6S,10aS,10bS)-2-(furan-3-yl)-10b-methyl-4-oxo-6-[(2R,3R,4S,5R,6R)-3,4,5-triacetyloxy-6-(acetyloxymethyl)oxan-2-yl]oxy-2,4a,5,6,8,9,10,10a-octahydro-1H-benzo[f]isochromene-7-carboxylate |
| 163069224 | methyl (2S,4aS,6S,6aR,10aR,10bS)-2-(furan-3-yl)-4a,8-dihydroxy-6a,10b-dimethyl-4-oxo-6-[(2R,3R,4S,5S,6R)-3,4,5-trihydroxy-6-(hydroxymethyl)oxan-2-yl]oxy-2,5,6,9,10,10a-hexahydro-1H-benzo[f]isochromene-7-carboxylate |
| 163070745 | [(1R,2R,7S,8R,9R,10S,11R,15R,16S)-15-(furan-3-yl)-7,9-dihydroxy-2,6,6,11,16-pentamethyl-5,14-dioxo-10-tetracyclo[9.7.0.02,8.012,16]octadeca-3,12-dienyl] acetate |
| 163072561 | (5S)-5-(4-methoxyphenyl)-2-phenyl-4,5-dihydro-1,3-oxazole |
| 163075353 | (1R,3R,4S,8R,8aS)-3,4,8,8a-tetramethyl-4-[(3S)-3-methylpentyl]-1,2,3,6,7,8-hexahydronaphthalen-1-ol |
| 163075826 | [(1R,2R,5R,6R,11R,12S,13R,16R,17R,19S,20R)-6-(furan-2-yl)-12,17-dihydroxy-1,5,11,16-tetramethyl-8-oxo-7,14-dioxapentacyclo[11.6.1.02,11.05,10.016,20]icos-9-en-19-yl] 2,3-dimethylbut-2-enoate |
| 163075861 | methyl 2-[(1S,2R,3R,4R,8R,9S,10R,13R,15R)-4-formyl-13-(furan-3-yl)-2-hydroxy-4,8,10,12-tetramethyl-7-oxo-16-oxatetracyclo[8.6.0.03,8.011,15]hexadeca-5,11-dien-9-yl]acetate |
| 163078747 | [(1S,2R,3R,5R,7R,10S,11R,14R,15S)-15-[(2R,3S,5R)-5-[(2S)-3,3-dimethyloxiran-2-yl]-2-hydroxyoxolan-3-yl]-3-hydroxy-2,6,6,10-tetramethyl-7-pentacyclo[12.3.1.01,14.02,11.05,10]octadecanyl] 3-methylbutanoate |
| 163080755 | [(5R,6R,7S,8R,9R,10R,13S,17R)-6-hydroxy-17-[(2S)-2-methoxy-5-oxo-2H-furan-4-yl]-4,4,8,10,13-pentamethyl-3-oxo-5,6,7,9,11,12,16,17-octahydrocyclopenta[a]phenanthren-7-yl] acetate |
| 163082705 | [(5R,6R,7S,8R,9R,10R,13S,17S)-6-methoxy-4,4,8,10,13-pentamethyl-3,16-dioxo-17-[(2S)-1-oxobutan-2-yl]-6,7,9,11,12,17-hexahydro-5H-cyclopenta[a]phenanthren-7-yl] acetate |
| 163082706 | [(5S,6R,7S,8R,9R,10S,13S,17S)-6-methoxy-4,4,8,10,13-pentamethyl-3,16-dioxo-17-[(2S)-1-oxobutan-2-yl]-6,7,9,11,12,17-hexahydro-5H-cyclopenta[a]phenanthren-7-yl] acetate |
| 163084105 | [(5R,7R,8R,9S,10S,11S,12R,13S,17R)-11-acetyloxy-12-hydroxy-17-[(2R)-2-hydroxy-5-oxo-2H-furan-3-yl]-4,4,8,10,13-pentamethyl-3-oxo-5,6,7,9,11,12,16,17-octahydrocyclopenta[a]phenanthren-7-yl] acetate |
| 163085934 | [(5R,7R,8R,9R,10R,13S,17S)-17-[(2S,3R)-2-ethoxyoxolan-3-yl]-4,4,8,10,13-pentamethyl-3-oxo-5,6,7,9,11,12,16,17-octahydrocyclopenta[a]phenanthren-7-yl] acetate |
| 163086875 | methyl 2-[(1S,2R,3R,4R,8R,9S,10R,13R,15R)-2-acetyloxy-13-(furan-3-yl)-4-(hydroxymethyl)-4,8,10,12-tetramethyl-7-oxo-16-oxatetracyclo[8.6.0.03,8.011,15]hexadeca-5,11-dien-9-yl]acetate |
| 163105183 | (1R,2R,6S,7S,8S)-1,5-dimethyl-8-[2-[(2S,3R,4S,5S,6R)-3,4,5-trihydroxy-6-(hydroxymethyl)oxan-2-yl]oxypropan-2-yl]tricyclo[4.4.0.02,7]dec-4-en-3-one |
| 163105607 | 4-(8,10-Dimethyl-2-methylideneundeca-3,5,7-trienyl)-2-methylbenzene-1,3-dicarboxylic acid |
| 163115075 | methyl 2-acetyloxy-13-(2-hydroxy-5-oxo-2H-furan-4-yl)-9-(2-methoxy-2-oxoethyl)-4,8,10,12-tetramethyl-7-oxo-16-oxatetracyclo[8.6.0.03,8.011,15]hexadeca-5,11-diene-4-carboxylate |
| 163185371 | [(1R,2S,4R,6R,9R,10R,11R,12S,14S,15R,18R)-6-(furan-3-yl)-14-hydroxy-10-(2-methoxy-2-oxoethyl)-7,9,11,15-tetramethyl-3,17-dioxapentacyclo[9.6.1.02,9.04,8.015,18]octadec-7-en-12-yl] (E)-2-methylbut-2-enoate |
| 163185419 | (1R)-1-[(2R,4R,5R)-5-hydroxy-4-[(3S,5R,9S,10R,13S,14S,17S)-3-hydroxy-4,4,10,13,14-pentamethyl-2,3,5,6,9,11,12,15,16,17-decahydro-1H-cyclopenta[a]phenanthren-17-yl]oxolan-2-yl]-2-methylpropane-1,2-diol |
| 163185622 | (E)-N-[(2S)-2-methoxy-2-[4-(3-methylbut-2-enoxy)phenyl]ethyl]-3-phenylprop-2-enamide |
| 163185892 | (1S,2S,4R,4aS,5R,8aS)-1-[(2S)-2-(furan-3-yl)-2-hydroxyethyl]-2,5-dihydroxy-5-methoxycarbonyl-1,4a-dimethyl-4-[(2R,3R,4R,5R,6S)-3,4,5-trihydroxy-6-(hydroxymethyl)oxan-2-yl]oxy-3,4,6,7,8,8a-hexahydronaphthalene-2-carboxylic acid |
| 163186393 | [(1S,4R,5R,6R)-5-[2-(furan-3-yl)ethyl]-4-(hydroxymethyl)-5-methyl-6-pentylcyclohex-2-en-1-yl]methanol |
| 163186826 | (1S,2R,4S,8aR)-1-[(2S)-2-(furan-3-yl)-2-hydroxyethyl]-2-hydroxy-5-methoxycarbonyl-1-methyl-4-[(2S,3S,4R,5S,6R)-3,4,5-trihydroxy-6-(hydroxymethyl)oxan-2-yl]oxy-3,4,6,7,8,8a-hexahydronaphthalene-2-carboxylic acid |
| 163186897 | (E)-N-[(2S)-2-ethoxy-2-(4-methoxyphenyl)ethyl]-3-phenylprop-2-enamide |
| 163187092 | (2S)-5,7-dihydroxy-2-[3-(3-hydroxy-3-methylbutyl)-4-methoxyphenyl]-8-[(1E)-3-methylbuta-1,3-dienyl]-2,3-dihydrochromen-4-one |
| 163187632 | (E)-N-[(2R)-2-hydroxy-2-[4-(3-methylbut-2-enoxy)phenyl]ethyl]-3-phenylprop-2-enamide |
| 163188089 | [(1R,2R,3S,5S,8R,10R,11S,12R,14S,16S,17S)-16-(furan-3-yl)-2,7,7,11,17-pentamethyl-6,15-dioxo-4,13-dioxahexacyclo[9.8.0.02,8.03,5.012,14.012,17]nonadecan-10-yl] acetate |
| 163188272 | (E)-N-[(2R)-2-hydroxy-2-[4-[(2S)-2-hydroxy-3-methylbut-3-enoxy]phenyl]ethyl]-3-phenylprop-2-enamide |
| 163188791 | [(1R,2S,4R,6R,9R,10R,11R,12S,14S,15R,18R)-14-acetyloxy-6-(furan-3-yl)-10-(2-methoxy-2-oxoethyl)-7,9,11,15-tetramethyl-3,17-dioxapentacyclo[9.6.1.02,9.04,8.015,18]octadec-7-en-12-yl] (E)-2-methylbut-2-enoate |
| 163189088 | [(1R)-1-[4-(3-methylbut-2-enoxy)phenyl]-2-[[(E)-3-phenylprop-2-enoyl]amino]ethyl] acetate |
| 163190772 | GlcA(b1-3)Gal(b1-3)b-Gal |
| 163192272 | 5-Methyl-5-phenylmethoxycyclohexa-1,3-dien-1-ol |
| 163193473 | methyl (2S,4aS,6S,6aR,10aR,10bS)-2-(furan-3-yl)-4a,8-dihydroxy-6a,10b-dimethyl-4-oxo-6-[(2S,3S,4R,5S,6R)-3,4,5-trihydroxy-6-(hydroxymethyl)oxan-2-yl]oxy-2,5,6,9,10,10a-hexahydro-1H-benzo[f]isochromene-7-carboxylate |
| 163193561 | methyl 2-[(1S,2R,3R,4R,8R,9R,10R,13R,15S)-2-acetyloxy-13-(furan-3-yl)-4-hydroxy-4,8,10,12-tetramethyl-7-oxo-16-oxatetracyclo[8.6.0.03,8.011,15]hexadeca-5,11-dien-9-yl]acetate |
| 163193927 | [(1S,2R,4R,5S,6S,10R,11R,13R,14R,15S,18S,20R)-4,15-diacetyloxy-6-(furan-3-yl)-11,18-dihydroxy-5,10,14-trimethyl-3-oxo-16-oxapentacyclo[12.3.3.01,13.02,10.05,9]icos-8-en-20-yl] acetate |
| 163194738 | [(5R,7R,8R,9R,10R,12S,13S,14R,17S)-12-hydroxy-17-[(2S,3S,5R)-2-hydroxy-5-(hydroxymethyl)oxolan-3-yl]-4,4,8,10,13-pentamethyl-3-oxo-5,6,7,9,11,12,14,15,16,17-decahydrocyclopenta[a]phenanthren-7-yl] (E)-3-(4-hydroxy-3-methoxyphenyl)prop-2-enoate |
| 163195533 | [(1R,2R,4R,5S,6S,10R,11S,12R,15R,16R,18S,19R)-16,18-diacetyloxy-6-(furan-3-yl)-4-hydroxy-1,5,10,15-tetramethyl-13-oxapentacyclo[10.6.1.02,10.05,9.015,19]nonadec-8-en-11-yl] (E)-2-methylbut-2-enoate |
