# Supplementary material for: Antidiabetic potential of a novel hydroxyphenyl-bi-benzopyran-hexol compound from Cassia fistula as an α-amylase inhibitor: Integrated in silico screening and in vitro validation using a nonlinear regression model
Source: J Ayurveda Integr Med. 2026 Jul 24;17(4):101366. doi: 10.1016/j.jaim.2026.101366 (PMC13420717; doi:10.1016/j.jaim.2026.101366)
Supplement: Multimedia component 2 [file mmc2.docx]

**Table S2** ADME Properties of the natural compounds

| **Compound ID** | **P1** | **P2** | **P3** | **P4** | **P5** | **P6** | **P7** | **P8** | **P9** | **P10** | **P11** | **P12** | **P13** | **P14** | **P15** | **P16** | **P17** | **P18** |
| --- | --- | --- | --- | --- | --- | --- | --- | --- | --- | --- | --- | --- | --- | --- | --- | --- | --- | --- |
| 305 | Low | No | No | No | No | No | No | No | -7.22 | 0 | 2 | 0 | 0 | 1 | 0.55 | 0 | 1 | 1 |
| 335 | High | Yes | No | Yes | No | No | No | No | -5.58 | 0 | 3 | 0 | 0 | 2 | 0.55 | 0 | 0 | 1 |
| 342 | High | Yes | No | Yes | No | No | No | No | -5.57 | 0 | 3 | 0 | 0 | 2 | 0.55 | 0 | 0 | 1 |
| 370 | High | No | No | No | No | No | No | Yes | -6.84 | 0 | 2 | 0 | 0 | 1 | 0.56 | 1 | 1 | 1.22 |
| 460 | High | Yes | No | No | No | No | No | No | -6.12 | 0 | 3 | 0 | 0 | 1 | 0.55 | 0 | 0 | 1 |
| 753 | High | No | No | No | No | No | No | No | -8.11 | 0 | 4 | 0 | 0 | 2 | 0.55 | 0 | 0 | 1.31 |
| 860 | High | Yes | No | Yes | No | Yes | No | No | -3.41 | 1 | 1 | 1 | 0 | 1 | 0.85 | 0 | 1 | 3.03 |
| 965 | High | No | No | Yes | No | Yes | No | No | -2.6 | 1 | 1 | 1 | 1 | 1 | 0.85 | 0 | 1 | 3.07 |
| 1183 | High | Yes | No | No | No | No | No | No | -6.37 | 0 | 2 | 0 | 0 | 1 | 0.55 | 0 | 1 | 1.15 |
| 1203 | High | No | Yes | No | No | No | No | No | -7.82 | 0 | 0 | 0 | 0 | 0 | 0.55 | 1 | 1 | 3.5 |
| 2263 | Low | No | Yes | No | No | No | No | No | -9.92 | 2 | 3 | 1 | 1 | 4 | 0.17 | 0 | 3 | 8.11 |
| 2353 | High | Yes | Yes | Yes | No | No | Yes | Yes | -5.78 | 0 | 0 | 0 | 0 | 0 | 0.55 | 0 | 1 | 3.14 |
| 2355 | High | Yes | No | Yes | No | No | No | No | -6.25 | 0 | 0 | 0 | 0 | 0 | 0.55 | 0 | 1 | 2.9 |
| 2879 | High | Yes | No | Yes | No | No | No | No | -5.58 | 0 | 3 | 0 | 0 | 2 | 0.55 | 0 | 0 | 1 |
| 3220 | High | No | No | Yes | No | No | No | Yes | -6.02 | 0 | 0 | 0 | 0 | 0 | 0.55 | 1 | 0 | 2.57 |
| 3248 | High | Yes | No | No | No | No | No | No | -6.43 | 0 | 0 | 0 | 0 | 1 | 0.55 | 0 | 2 | 2.2 |
| 3893 | High | Yes | No | No | No | No | No | No | -4.54 | 0 | 0 | 0 | 0 | 0 | 0.85 | 0 | 0 | 1.87 |
| 3931 | High | Yes | No | Yes | No | Yes | No | No | -3.05 | 1 | 1 | 1 | 1 | 1 | 0.85 | 0 | 1 | 3.1 |
| 4114 | High | Yes | No | Yes | No | No | No | No | -6.2 | 0 | 0 | 0 | 0 | 0 | 0.55 | 0 | 1 | 2.97 |
| 5793 | Low | No | Yes | No | No | No | No | No | -9.7 | 0 | 2 | 0 | 0 | 2 | 0.55 | 0 | 0 | 4.08 |
| 5984 | Low | No | No | No | No | No | No | No | -9.7 | 0 | 2 | 0 | 0 | 2 | 0.55 | 0 | 0 | 3.13 |
| 5988 | Low | No | Yes | No | No | No | No | No | -11 | 2 | 1 | 1 | 1 | 4 | 0.17 | 0 | 0 | 5.16 |
| 5997 | Low | No | No | No | No | Yes | No | No | -2.47 | 1 | 2 | 0 | 1 | 2 | 0.55 | 0 | 1 | 5.98 |
| 6054 | High | Yes | No | Yes | No | No | No | No | -6.08 | 0 | 3 | 0 | 0 | 2 | 0.55 | 0 | 0 | 1 |
| 6214 | Low | Yes | No | No | No | Yes | No | No | -4.8 | 0 | 1 | 0 | 0 | 2 | 0.55 | 0 | 1 | 2.5 |
| 6466 | High | No | Yes | No | No | No | No | No | -8.24 | 0 | 0 | 0 | 0 | 0 | 0.56 | 0 | 1 | 6.69 |
| 6549 | High | Yes | No | No | No | No | No | No | -5.13 | 0 | 1 | 0 | 0 | 2 | 0.55 | 0 | 1 | 2.74 |
| 6654 | Low | Yes | No | No | No | Yes | No | No | -3.95 | 1 | 1 | 0 | 0 | 2 | 0.55 | 0 | 1 | 4.44 |
| 6656 | High | Yes | No | No | No | No | No | No | -6.21 | 0 | 3 | 0 | 0 | 1 | 0.85 | 0 | 1 | 1.42 |
| 6760 | High | Yes | No | Yes | Yes | Yes | Yes | Yes | -5.87 | 0 | 0 | 0 | 0 | 0 | 0.55 | 0 | 0 | 2.93 |
| 6923 | High | Yes | No | Yes | No | No | No | No | -4.87 | 0 | 1 | 0 | 0 | 2 | 0.55 | 0 | 0 | 1 |
| 6987 | High | Yes | No | No | No | No | No | No | -5.21 | 0 | 1 | 0 | 0 | 2 | 0.55 | 0 | 0 | 3.2 |
| 7213 | High | Yes | No | No | No | No | No | Yes | -6.76 | 0 | 3 | 0 | 0 | 1 | 0.55 | 0 | 1 | 1 |
| 7460 | Low | Yes | No | No | No | No | No | No | -4.85 | 0 | 1 | 0 | 0 | 2 | 0.55 | 0 | 0 | 4.15 |
| 7461 | Low | Yes | No | No | No | No | No | No | -3.94 | 0 | 1 | 0 | 0 | 2 | 0.55 | 0 | 1 | 3.11 |
| 7463 | Low | Yes | No | No | No | No | Yes | No | -4.21 | 1 | 1 | 0 | 0 | 2 | 0.55 | 0 | 0 | 1 |
| 7478 | High | Yes | No | No | No | No | No | No | -5.84 | 0 | 3 | 0 | 0 | 1 | 0.85 | 0 | 0 | 1 |
| 7800 | High | Yes | No | Yes | No | No | No | No | -3.64 | 0 | 0 | 1 | 0 | 1 | 0.55 | 0 | 0 | 2.35 |
| 8163 | High | Yes | No | Yes | No | No | No | No | -4.43 | 0 | 0 | 0 | 0 | 2 | 0.55 | 0 | 0 | 1.72 |
| 8181 | High | Yes | No | Yes | No | No | No | No | -2.71 | 1 | 1 | 1 | 0 | 1 | 0.55 | 0 | 0 | 2.53 |
| 8193 | High | Yes | No | Yes | No | No | No | No | -3.79 | 0 | 0 | 0 | 0 | 3 | 0.55 | 0 | 0 | 1.85 |
| 8201 | High | No | No | Yes | No | No | No | No | -2.19 | 1 | 1 | 1 | 1 | 2 | 0.55 | 0 | 0 | 2.76 |
| 8203 | High | No | No | Yes | No | Yes | No | No | -3.25 | 1 | 1 | 1 | 1 | 1 | 0.55 | 0 | 1 | 3.18 |
| 8222 | Low | No | No | Yes | No | No | No | No | -0.6 | 1 | 1 | 1 | 1 | 3 | 0.55 | 0 | 0 | 2.72 |
| 8417 | High | Yes | No | Yes | No | No | No | No | -6.34 | 0 | 0 | 0 | 0 | 0 | 0.55 | 0 | 1 | 2.77 |
| 8468 | High | No | No | No | No | No | No | No | -6.31 | 0 | 0 | 0 | 0 | 1 | 0.85 | 0 | 0 | 1.42 |
| 9064 | High | No | Yes | No | No | No | No | No | -7.82 | 0 | 0 | 0 | 0 | 0 | 0.55 | 1 | 1 | 3.5 |
| 10168 | High | No | No | No | No | No | No | No | -6.45 | 0 | 0 | 0 | 0 | 0 | 0.56 | 1 | 0 | 2.58 |
| 10205 | High | Yes | No | Yes | No | No | No | No | -5.82 | 0 | 0 | 0 | 0 | 1 | 0.55 | 2 | 0 | 2.41 |
| 10207 | High | No | No | Yes | No | No | No | Yes | -6.66 | 0 | 0 | 0 | 0 | 0 | 0.55 | 1 | 0 | 2.6 |
| 10208 | High | Yes | No | Yes | No | No | No | Yes | -5.34 | 0 | 0 | 0 | 0 | 0 | 0.55 | 1 | 0 | 2.47 |
| 10212 | High | Yes | No | Yes | Yes | Yes | No | No | -5.46 | 0 | 0 | 0 | 0 | 0 | 0.55 | 0 | 2 | 3.22 |
| 10393 | High | Yes | No | No | No | No | No | No | -6.84 | 0 | 2 | 0 | 0 | 1 | 0.55 | 0 | 0 | 1 |
| 10416 | High | Yes | No | Yes | No | No | No | No | -3.61 | 1 | 1 | 1 | 0 | 1 | 0.85 | 0 | 1 | 3.2 |
| 10469 | Low | No | No | No | No | No | No | No | 0.18 | 1 | 2 | 1 | 1 | 2 | 0.85 | 0 | 0 | 3.49 |
| 10582 | High | Yes | No | No | No | No | No | No | -4.94 | 0 | 1 | 0 | 0 | 2 | 0.55 | 0 | 1 | 4.22 |
| 10639 | High | No | No | Yes | No | Yes | No | Yes | -5.88 | 0 | 0 | 0 | 0 | 0 | 0.55 | 1 | 0 | 2.69 |
| 10964 | High | No | No | No | No | No | No | No | -7.19 | 0 | 3 | 0 | 0 | 2 | 0.55 | 0 | 2 | 1 |
| 11005 | High | Yes | No | Yes | No | No | No | No | -3.35 | 0 | 0 | 1 | 0 | 1 | 0.85 | 0 | 0 | 2.09 |
| 11197 | Low | No | No | Yes | No | No | No | No | -0.41 | 1 | 2 | 1 | 1 | 2 | 0.85 | 0 | 0 | 3.24 |
| 11230 | High | Yes | No | No | No | No | No | No | -4.93 | 0 | 1 | 0 | 0 | 2 | 0.55 | 0 | 1 | 3.28 |
| 11463 | Low | Yes | No | No | No | Yes | No | No | -3.96 | 0 | 1 | 0 | 0 | 2 | 0.55 | 0 | 1 | 2.98 |
| 11622 | High | Yes | No | Yes | No | No | No | No | -3.84 | 0 | 0 | 0 | 0 | 3 | 0.55 | 0 | 0 | 1.93 |
| 11636 | Low | No | Yes | No | No | No | No | No | 1.49 | 1 | 3 | 1 | 1 | 3 | 0.55 | 0 | 0 | 3.56 |
| 11850 | Low | No | No | No | No | No | No | No | -9.61 | 1 | 2 | 0 | 0 | 3 | 0.55 | 0 | 0 | 3.3 |
| 12177 | High | Yes | No | No | No | No | No | No | -5.94 | 0 | 3 | 0 | 0 | 2 | 0.55 | 0 | 2 | 1.92 |
| 12366 | High | No | No | Yes | No | No | No | No | -2.44 | 1 | 1 | 1 | 1 | 2 | 0.55 | 0 | 0 | 2.8 |
| 12377 | High | Yes | No | No | No | No | No | No | -5.3 | 0 | 1 | 0 | 0 | 1 | 0.55 | 0 | 1 | 2.79 |
| 12405 | Low | No | No | Yes | No | No | No | No | -0.01 | 1 | 1 | 1 | 1 | 3 | 0.55 | 0 | 0 | 2.96 |
| 12409 | Low | No | Yes | No | No | No | No | No | 2.08 | 1 | 3 | 1 | 1 | 3 | 0.55 | 0 | 0 | 3.81 |
| 12410 | Low | No | Yes | No | No | No | No | No | 2.69 | 1 | 3 | 1 | 1 | 3 | 0.55 | 0 | 0 | 4.06 |
| 12575 | High | No | No | No | No | No | No | No | -6.31 | 0 | 0 | 0 | 0 | 1 | 0.85 | 0 | 0 | 1.24 |
| 12921 | High | No | No | Yes | No | No | No | No | -3.46 | 1 | 1 | 1 | 1 | 1 | 0.85 | 0 | 1 | 3.32 |
| 13187 | High | Yes | No | No | No | No | No | No | -4.94 | 0 | 1 | 0 | 0 | 2 | 0.55 | 0 | 0 | 1.52 |
| 14896 | Low | Yes | No | No | No | Yes | No | No | -4.18 | 1 | 1 | 0 | 0 | 2 | 0.55 | 0 | 1 | 3.73 |
| 16592 | High | Yes | No | No | No | No | No | No | -5.77 | 0 | 3 | 0 | 0 | 2 | 0.55 | 0 | 1 | 2.33 |
| 18818 | Low | Yes | No | No | No | No | No | No | -4.94 | 1 | 1 | 0 | 0 | 2 | 0.55 | 0 | 1 | 2.87 |
| 19009 | High | Yes | Yes | No | No | No | Yes | Yes | -5.79 | 0 | 0 | 0 | 0 | 0 | 0.55 | 0 | 1 | 3.18 |
| 22311 | Low | Yes | No | No | No | Yes | No | No | -3.89 | 0 | 1 | 0 | 0 | 2 | 0.55 | 0 | 1 | 3.46 |
| 22383 | High | Yes | No | No | No | No | No | No | -5.18 | 0 | 0 | 0 | 0 | 1 | 0.55 | 0 | 1 | 3.76 |
| 26305 | High | Yes | No | Yes | No | No | No | No | -6.45 | 0 | 0 | 0 | 0 | 0 | 0.55 | 0 | 1 | 3.35 |
| 26519 | Low | No | Yes | No | No | No | No | No | 3.58 | 1 | 3 | 1 | 1 | 3 | 0.55 | 0 | 0 | 4.45 |
| 31238 | Low | No | No | Yes | No | No | No | No | -1.24 | 1 | 1 | 1 | 1 | 2 | 0.55 | 0 | 0 | 1.99 |
| 31245 | High | Yes | No | No | No | No | No | No | -5.73 | 0 | 3 | 0 | 0 | 2 | 0.55 | 0 | 1 | 1.16 |
| 31253 | Low | Yes | No | No | No | No | No | No | -4.17 | 0 | 1 | 0 | 0 | 2 | 0.55 | 0 | 2 | 2.85 |
| 31289 | High | Yes | No | No | No | No | No | No | -4.85 | 0 | 1 | 0 | 0 | 2 | 0.55 | 0 | 1 | 1.52 |
| 31291 | High | Yes | No | Yes | No | No | No | No | -3.36 | 0 | 0 | 1 | 0 | 2 | 0.55 | 0 | 1 | 2.04 |
| 35349 | High | Yes | No | No | No | No | No | No | -5.59 | 0 | 2 | 0 | 0 | 1 | 0.55 | 0 | 1 | 2.7 |
| 60961 | Low | No | No | No | No | No | No | No | -8.68 | 0 | 1 | 0 | 1 | 0 | 0.55 | 0 | 0 | 3.86 |
| 64971 | Low | No | No | No | No | Yes | No | No | -3.26 | 1 | 3 | 0 | 1 | 1 | 0.85 | 0 | 1 | 5.63 |
| 65084 | High | No | No | No | No | No | No | No | -8.17 | 1 | 0 | 0 | 0 | 1 | 0.55 | 1 | 1 | 3.53 |
| 68066 | High | No | No | No | No | No | No | No | -5.51 | 0 | 0 | 0 | 0 | 0 | 0.55 | 0 | 2 | 2.93 |
| 68171 | Low | No | No | No | No | No | No | No | 0.26 | 1 | 2 | 1 | 1 | 3 | 0.55 | 0 | 0 | 3.47 |
| 68406 | Low | No | Yes | No | No | No | No | No | 0.86 | 1 | 3 | 1 | 1 | 3 | 0.55 | 0 | 0 | 3.72 |
| 69502 | High | Yes | No | Yes | Yes | Yes | No | No | -5.19 | 0 | 0 | 0 | 0 | 0 | 0.55 | 0 | 2 | 3.2 |
| 69894 | High | Yes | No | Yes | No | No | No | No | -6.49 | 0 | 0 | 0 | 0 | 1 | 0.55 | 0 | 1 | 2.65 |
| 70954 | Low | Yes | No | No | No | No | No | No | -4.72 | 1 | 1 | 0 | 0 | 2 | 0.55 | 0 | 1 | 3.36 |
| 72276 | High | No | Yes | No | No | No | No | No | -7.82 | 0 | 0 | 0 | 0 | 0 | 0.55 | 1 | 1 | 3.5 |
| 72277 | High | No | No | No | No | No | No | No | -8.17 | 1 | 0 | 0 | 0 | 1 | 0.55 | 1 | 1 | 3.53 |
| 73111 | Low | No | Yes | No | No | No | No | No | -10.7 | 3 | 4 | 1 | 1 | 5 | 0.11 | 0 | 0 | 7.51 |
| 73145 | Low | No | No | No | No | No | No | No | -2.41 | 1 | 3 | 0 | 1 | 2 | 0.55 | 0 | 1 | 6.04 |
| 73170 | Low | No | No | No | No | No | No | No | -2.51 | 1 | 3 | 0 | 1 | 2 | 0.55 | 0 | 1 | 6.17 |
| 73337 | High | Yes | Yes | Yes | No | No | No | Yes | -6.44 | 0 | 0 | 0 | 0 | 0 | 0.55 | 0 | 1 | 3.78 |
| 74138 | Low | No | Yes | Yes | No | No | No | No | 0.48 | 1 | 1 | 1 | 1 | 3 | 0.55 | 0 | 1 | 3.28 |
| 75997 | Low | No | Yes | No | No | No | No | No | 0.72 | 1 | 3 | 1 | 1 | 3 | 0.55 | 0 | 0 | 3.81 |
| 76015 | High | Yes | No | No | No | No | No | No | -4.62 | 0 | 0 | 0 | 0 | 2 | 0.55 | 0 | 1 | 2.96 |
| 76295 | High | No | No | Yes | No | No | No | Yes | -5.48 | 0 | 0 | 0 | 0 | 0 | 0.55 | 1 | 1 | 2.38 |
| 77409 | High | Yes | No | Yes | No | No | No | No | -6.29 | 0 | 0 | 0 | 0 | 0 | 0.55 | 0 | 1 | 2.77 |
| 79089 | High | Yes | No | Yes | No | No | No | No | -5.33 | 0 | 3 | 0 | 0 | 2 | 0.55 | 0 | 0 | 1.73 |
| 80048 | Low | No | No | No | No | Yes | No | No | -4 | 1 | 0 | 0 | 0 | 1 | 0.55 | 0 | 1 | 4.26 |
| 81696 | Low | No | Yes | No | No | No | No | No | -9.7 | 0 | 2 | 0 | 0 | 2 | 0.55 | 0 | 0 | 4.08 |
| 83412 | High | Yes | No | Yes | No | No | No | No | -5.86 | 0 | 0 | 0 | 0 | 0 | 0.55 | 0 | 0 | 2.82 |
| 91440 | Low | No | Yes | No | No | No | No | No | -10.7 | 3 | 4 | 1 | 1 | 5 | 0.11 | 0 | 0 | 7.51 |
| 92139 | Low | No | No | No | No | No | Yes | No | -3.71 | 1 | 0 | 0 | 0 | 2 | 0.55 | 0 | 1 | 2.31 |
| 92221 | Low | Yes | No | No | No | Yes | No | No | -4.13 | 1 | 1 | 0 | 0 | 2 | 0.55 | 0 | 1 | 3.5 |
| 94162 | High | Yes | No | No | Yes | Yes | No | No | -4.14 | 0 | 0 | 0 | 0 | 1 | 0.55 | 0 | 0 | 3.45 |
| 94221 | High | Yes | No | No | No | No | No | No | -5.01 | 0 | 1 | 0 | 0 | 2 | 0.55 | 0 | 1 | 3.61 |
| 94249 | Low | Yes | No | No | No | Yes | No | No | -3.77 | 1 | 1 | 0 | 0 | 2 | 0.55 | 0 | 0 | 2.77 |
| 94403 | High | Yes | No | Yes | No | Yes | No | No | -3.72 | 0 | 0 | 0 | 0 | 1 | 0.55 | 0 | 1 | 3.27 |
| 98570 | High | Yes | Yes | Yes | No | Yes | Yes | Yes | -6.48 | 0 | 0 | 0 | 0 | 0 | 0.55 | 0 | 0 | 3.63 |
| 100017 | High | No | Yes | No | No | No | No | No | -7.61 | 0 | 0 | 0 | 0 | 0 | 0.55 | 0 | 2 | 6.07 |
| 101977 | High | Yes | No | No | No | No | No | No | -4.48 | 0 | 1 | 0 | 0 | 2 | 0.55 | 0 | 1 | 2.61 |
| 104285 | High | No | No | No | No | Yes | No | No | -5.07 | 0 | 0 | 0 | 0 | 0 | 0.55 | 0 | 2 | 3.59 |
| 104884 | Low | No | Yes | No | No | No | No | No | -7.15 | 1 | 3 | 0 | 0 | 0 | 0.55 | 0 | 3 | 7.04 |
| 106648 | High | Yes | No | Yes | No | No | No | No | -6.44 | 0 | 0 | 0 | 0 | 0 | 0.55 | 0 | 1 | 2.62 |
| 107526 | Low | No | No | No | No | No | No | No | -9.49 | 0 | 2 | 0 | 0 | 2 | 0.55 | 0 | 1 | 3.31 |
| 107936 | High | Yes | No | Yes | Yes | No | Yes | Yes | -5.66 | 0 | 0 | 0 | 0 | 0 | 0.55 | 0 | 0 | 2.77 |
| 108058 | High | No | No | No | No | No | No | No | -7.98 | 1 | 3 | 0 | 0 | 0 | 0.55 | 0 | 2 | 6.54 |
| 122738 | Low | No | No | No | No | No | No | Yes | -8.15 | 3 | 2 | 1 | 1 | 3 | 0.17 | 1 | 1 | 5.32 |
| 125213 | High | No | No | No | No | No | Yes | Yes | -6.72 | 0 | 0 | 0 | 0 | 0 | 0.55 | 0 | 1 | 2.55 |
| 125468 | High | Yes | No | No | No | No | No | No | -6.21 | 0 | 3 | 0 | 0 | 1 | 0.85 | 0 | 1 | 1.42 |
| 126566 | High | No | Yes | No | No | No | No | Yes | -6.89 | 1 | 3 | 0 | 0 | 0 | 0.55 | 0 | 1 | 6.39 |
| 129754 | High | No | Yes | No | No | No | No | No | -5.77 | 1 | 3 | 0 | 0 | 1 | 0.55 | 0 | 1 | 6.97 |
| 138824 | High | Yes | Yes | No | No | No | No | No | -3.38 | 1 | 0 | 0 | 0 | 2 | 0.55 | 0 | 0 | 2.89 |
| 157277 | High | No | Yes | No | No | No | No | No | -8.47 | 0 | 0 | 0 | 0 | 0 | 0.55 | 0 | 1 | 6.07 |
| 159931 | Low | No | No | No | No | No | No | No | -2.95 | 1 | 3 | 0 | 1 | 1 | 0.55 | 0 | 0 | 5.46 |
| 167718 | High | Yes | Yes | No | No | No | Yes | Yes | -6.25 | 0 | 0 | 0 | 0 | 0 | 0.55 | 0 | 1 | 3.21 |
| 173183 | Low | No | No | No | No | No | No | No | -2.5 | 1 | 2 | 0 | 1 | 2 | 0.55 | 0 | 1 | 6.17 |
| 176920 | High | No | Yes | No | No | No | No | No | -8.02 | 0 | 0 | 0 | 0 | 0 | 0.55 | 0 | 0 | 3.65 |
| 176996 | High | No | No | No | No | No | No | Yes | -8.13 | 0 | 1 | 0 | 0 | 0 | 0.55 | 0 | 2 | 6.32 |
| 177090 | High | Yes | No | No | Yes | Yes | Yes | Yes | -4.16 | 0 | 0 | 0 | 0 | 1 | 0.55 | 0 | 0 | 3.42 |
| 178770 | High | No | Yes | No | No | No | No | No | -5.7 | 0 | 0 | 0 | 0 | 0 | 0.55 | 0 | 1 | 6.04 |
| 180429 | High | Yes | Yes | No | No | No | Yes | No | -5.26 | 0 | 0 | 0 | 0 | 0 | 0.55 | 0 | 0 | 3.68 |
| 180932 | Low | No | Yes | No | No | No | No | No | -9.76 | 2 | 2 | 1 | 1 | 2 | 0.17 | 0 | 2 | 6.48 |
| 184937 | High | No | Yes | No | No | No | No | Yes | -6.51 | 0 | 1 | 0 | 0 | 0 | 0.55 | 0 | 1 | 6.09 |
| 188289 | High | No | Yes | No | No | No | No | No | -6.95 | 0 | 0 | 0 | 0 | 0 | 0.55 | 0 | 2 | 5.37 |
| 189403 | High | Yes | Yes | No | Yes | No | No | Yes | -5.91 | 0 | 0 | 0 | 0 | 0 | 0.55 | 0 | 0 | 3.15 |
| 189404 | High | Yes | Yes | No | Yes | No | No | Yes | -5.91 | 0 | 0 | 0 | 0 | 0 | 0.55 | 0 | 0 | 3.16 |
| 189660 | High | Yes | Yes | No | No | No | Yes | Yes | -6.14 | 0 | 0 | 0 | 0 | 0 | 0.55 | 0 | 0 | 3.43 |
| 189704 | High | Yes | Yes | No | No | No | Yes | No | -6.07 | 0 | 0 | 0 | 0 | 0 | 0.55 | 0 | 0 | 3.57 |
| 189706 | High | Yes | Yes | No | No | No | No | Yes | -6.28 | 0 | 0 | 0 | 0 | 0 | 0.55 | 0 | 0 | 3.32 |
| 189726 | High | Yes | Yes | No | Yes | No | No | Yes | -6.17 | 0 | 0 | 0 | 0 | 0 | 0.85 | 0 | 0 | 3.34 |
| 189727 | High | Yes | Yes | No | Yes | No | No | Yes | -6.17 | 0 | 0 | 0 | 0 | 0 | 0.85 | 0 | 0 | 3.31 |
| 189728 | High | Yes | Yes | No | Yes | Yes | No | Yes | -4.77 | 0 | 0 | 0 | 0 | 0 | 0.85 | 0 | 0 | 3.35 |
| 193405 | High | Yes | No | No | No | No | Yes | No | -5.18 | 0 | 0 | 1 | 0 | 0 | 0.55 | 0 | 0 | 3.48 |
| 216283 | Low | No | No | No | No | No | No | No | -8.56 | 0 | 0 | 0 | 1 | 0 | 0.55 | 0 | 1 | 5.23 |
| 222284 | Low | No | No | No | No | No | No | No | -2.2 | 1 | 3 | 0 | 1 | 2 | 0.55 | 0 | 1 | 6.3 |
| 237332 | High | No | No | No | No | No | No | No | -7.48 | 0 | 3 | 0 | 0 | 1 | 0.55 | 0 | 1 | 2.25 |
| 259846 | Low | No | No | No | No | No | No | No | -1.9 | 1 | 3 | 0 | 1 | 2 | 0.55 | 0 | 1 | 5.49 |
| 267137 | High | Yes | No | No | No | No | No | No | -5.1 | 0 | 0 | 0 | 0 | 0 | 0.85 | 0 | 1 | 1.64 |
| 283510 | Low | No | Yes | Yes | No | No | No | No | 0.81 | 1 | 2 | 1 | 1 | 3 | 0.55 | 0 | 0 | 3.2 |
| 304040 | High | Yes | No | Yes | No | No | Yes | No | -4.52 | 0 | 0 | 0 | 0 | 2 | 0.55 | 0 | 0 | 2.06 |
| 330573 | High | Yes | No | No | No | No | No | No | -5.01 | 0 | 1 | 0 | 0 | 2 | 0.55 | 0 | 1 | 3.61 |
| 334704 | High | Yes | No | Yes | No | No | No | No | -6.45 | 0 | 0 | 0 | 0 | 0 | 0.55 | 0 | 1 | 3.35 |
| 361512 | High | No | No | No | No | No | No | Yes | -7 | 0 | 0 | 0 | 0 | 0 | 0.55 | 1 | 0 | 2.68 |
| 400073 | High | Yes | No | Yes | Yes | Yes | No | No | -4.5 | 0 | 0 | 0 | 0 | 1 | 0.55 | 0 | 2 | 3.43 |
| 439242 | Low | No | Yes | No | No | No | No | No | -13.5 | 3 | 2 | 1 | 1 | 4 | 0.17 | 0 | 0 | 6.28 |
| 439357 | Low | No | Yes | No | No | No | No | No | -9.7 | 0 | 2 | 0 | 0 | 2 | 0.55 | 0 | 0 | 4.08 |
| 439503 | Low | No | No | Yes | No | No | No | No | -8.91 | 0 | 1 | 0 | 0 | 0 | 0.55 | 0 | 0 | 4.23 |
| 439531 | Low | No | Yes | No | No | No | No | No | -16 | 3 | 4 | 2 | 1 | 5 | 0.17 | 0 | 0 | 7.32 |
| 440917 | Low | Yes | No | No | No | Yes | No | No | -3.89 | 0 | 1 | 0 | 0 | 2 | 0.55 | 0 | 1 | 3.46 |
| 440967 | Low | Yes | No | No | No | Yes | No | No | -4.18 | 1 | 1 | 0 | 0 | 2 | 0.55 | 0 | 1 | 3.73 |
| 440968 | Low | Yes | No | No | No | Yes | No | No | -3.95 | 1 | 1 | 0 | 0 | 2 | 0.55 | 0 | 1 | 4.44 |
| 441005 | Low | No | No | No | Yes | Yes | No | No | -4.85 | 1 | 0 | 0 | 0 | 1 | 0.55 | 0 | 1 | 4.14 |
| 442015 | High | No | Yes | No | No | No | No | No | -6.95 | 0 | 0 | 0 | 0 | 0 | 0.55 | 0 | 2 | 5.37 |
| 442068 | High | No | Yes | No | No | No | Yes | No | -7.64 | 0 | 0 | 0 | 0 | 0 | 0.55 | 0 | 2 | 5.27 |
| 442127 | High | Yes | No | Yes | No | No | No | No | -6.45 | 0 | 0 | 0 | 0 | 0 | 0.55 | 0 | 1 | 3.36 |
| 442153 | High | No | No | No | No | Yes | No | Yes | -4.27 | 0 | 0 | 0 | 0 | 1 | 0.55 | 0 | 1 | 4.16 |
| 442154 | High | No | Yes | No | No | No | No | No | -7.46 | 0 | 0 | 0 | 0 | 0 | 0.55 | 0 | 0 | 3.39 |
| 442359 | Low | Yes | No | Yes | Yes | Yes | No | No | -4.37 | 1 | 0 | 0 | 0 | 1 | 0.55 | 0 | 1 | 5.31 |
| 442731 | Low | No | Yes | No | No | No | No | No | -7.94 | 0 | 0 | 1 | 1 | 1 | 0.55 | 1 | 0 | 4.96 |
| 443158 | High | Yes | No | No | No | No | No | No | -5.13 | 0 | 1 | 0 | 0 | 2 | 0.55 | 0 | 1 | 2.74 |
| 443639 | High | No | Yes | No | No | No | No | No | -7.46 | 0 | 0 | 0 | 0 | 0 | 0.55 | 0 | 0 | 3.39 |
| 444539 | High | Yes | No | No | No | No | No | No | -5.69 | 0 | 2 | 0 | 0 | 1 | 0.85 | 0 | 1 | 1.67 |
| 444899 | High | No | No | Yes | No | Yes | No | No | -3.2 | 1 | 1 | 1 | 1 | 1 | 0.85 | 0 | 1 | 3.26 |
| 445638 | High | Yes | No | Yes | No | Yes | No | No | -3.18 | 0 | 0 | 1 | 0 | 1 | 0.85 | 0 | 1 | 2.84 |
| 445639 | High | No | No | Yes | No | Yes | No | No | -2.6 | 1 | 1 | 1 | 1 | 1 | 0.85 | 0 | 1 | 3.07 |
| 480764 | High | No | No | Yes | No | Yes | Yes | Yes | -5.22 | 0 | 0 | 0 | 0 | 0 | 0.55 | 0 | 1 | 3.67 |
| 484588 | High | No | No | Yes | No | Yes | Yes | Yes | -5.22 | 0 | 0 | 0 | 0 | 0 | 0.55 | 0 | 1 | 3.67 |
| 500060 | High | No | Yes | No | No | No | No | No | -5.63 | 0 | 0 | 0 | 0 | 0 | 0.55 | 0 | 0 | 5.89 |
| 518975 | Low | Yes | No | No | No | No | Yes | No | -3.96 | 1 | 0 | 0 | 0 | 2 | 0.55 | 0 | 0 | 2.4 |
| 520895 | High | Yes | No | No | No | Yes | No | No | -4.95 | 0 | 0 | 0 | 0 | 1 | 0.55 | 0 | 1 | 4.21 |
| 521941 | High | Yes | No | No | No | No | No | No | -5.6 | 0 | 2 | 0 | 0 | 1 | 0.55 | 0 | 1 | 2.37 |
| 522458 | High | Yes | No | No | No | No | No | No | -5.14 | 0 | 0 | 0 | 0 | 1 | 0.55 | 0 | 1 | 2.78 |
| 525330 | High | Yes | No | No | No | No | No | No | -5.35 | 0 | 0 | 0 | 0 | 1 | 0.55 | 0 | 2 | 3.54 |
| 529904 | High | Yes | No | No | No | No | No | No | -5.41 | 0 | 1 | 0 | 0 | 1 | 0.55 | 0 | 1 | 3.51 |
| 575174 | High | Yes | No | No | No | No | Yes | No | -5.18 | 0 | 0 | 1 | 0 | 0 | 0.55 | 0 | 0 | 3.48 |
| 584354 | High | Yes | No | Yes | No | No | No | No | -6.05 | 0 | 0 | 0 | 0 | 1 | 0.55 | 0 | 0 | 1.94 |
| 595524 | High | Yes | No | Yes | No | No | No | No | -5.56 | 0 | 0 | 0 | 0 | 1 | 0.55 | 0 | 0 | 2.64 |
| 600671 | High | Yes | No | Yes | No | No | No | No | -6.45 | 0 | 0 | 0 | 0 | 0 | 0.55 | 0 | 1 | 3.36 |
| 611513 | Low | No | No | No | No | No | No | No | -8.56 | 0 | 0 | 0 | 1 | 0 | 0.55 | 0 | 1 | 5.23 |
| 636663 | High | Yes | No | Yes | No | No | No | No | -6.26 | 0 | 0 | 0 | 0 | 0 | 0.55 | 0 | 1 | 2.61 |
| 636837 | High | No | Yes | No | No | Yes | No | No | -6.15 | 0 | 1 | 0 | 0 | 0 | 0.55 | 0 | 1 | 6.2 |
| 638072 | Low | No | No | No | No | No | No | No | -0.58 | 1 | 3 | 1 | 1 | 2 | 0.55 | 0 | 1 | 4.73 |
| 643654 | High | No | Yes | No | No | No | Yes | Yes | -5.98 | 0 | 3 | 1 | 0 | 0 | 0.55 | 0 | 2 | 5.33 |
| 1550607 | High | Yes | No | Yes | Yes | Yes | No | No | -4.5 | 0 | 0 | 0 | 0 | 1 | 0.55 | 0 | 2 | 3.43 |
| 1713001 | High | Yes | No | No | No | No | No | No | -4.86 | 0 | 0 | 0 | 0 | 2 | 0.55 | 0 | 1 | 3 |
| 1742210 | High | Yes | No | No | Yes | Yes | No | No | -5.12 | 0 | 0 | 0 | 0 | 1 | 0.55 | 0 | 2 | 4.35 |
| 1795390 | High | Yes | No | Yes | Yes | Yes | No | No | -4.5 | 0 | 0 | 0 | 0 | 1 | 0.55 | 0 | 2 | 3.43 |
| 1810796 | High | No | Yes | No | No | Yes | No | No | -3 | 1 | 1 | 1 | 1 | 2 | 0.55 | 0 | 0 | 3.2 |
| 2723872 | Low | No | No | No | No | No | No | No | -9.42 | 0 | 2 | 0 | 0 | 2 | 0.55 | 0 | 0 | 3.96 |
| 3034112 | High | No | Yes | No | No | No | No | No | -6.4 | 0 | 0 | 0 | 0 | 0 | 0.55 | 0 | 1 | 6.29 |
| 3084213 | High | No | No | No | No | No | No | No | -6.33 | 0 | 0 | 0 | 0 | 0 | 0.55 | 0 | 2 | 4.02 |
| 4482272 | Low | No | Yes | No | No | No | No | No | -7.15 | 1 | 3 | 0 | 0 | 0 | 0.55 | 0 | 3 | 7.04 |
| 5260170 | High | No | Yes | No | No | No | No | No | -7.3 | 1 | 3 | 0 | 0 | 0 | 0.55 | 0 | 3 | 6.86 |
| 5280343 | High | No | No | Yes | No | No | Yes | Yes | -7.05 | 0 | 0 | 0 | 0 | 0 | 0.55 | 1 | 1 | 3.23 |
| 5280435 | Low | No | Yes | No | No | Yes | No | No | -2.29 | 1 | 1 | 1 | 1 | 2 | 0.55 | 0 | 1 | 4.3 |
| 5280443 | High | No | No | Yes | No | No | Yes | Yes | -5.8 | 0 | 0 | 0 | 0 | 0 | 0.55 | 0 | 0 | 2.96 |
| 5280450 | High | Yes | No | Yes | No | Yes | No | No | -3.05 | 1 | 1 | 1 | 1 | 1 | 0.85 | 0 | 1 | 3.1 |
| 5280459 | Low | No | No | No | No | No | No | No | -8.42 | 2 | 0 | 1 | 1 | 3 | 0.17 | 1 | 1 | 5.28 |
| 5280460 | High | Yes | No | Yes | No | No | No | No | -6.39 | 0 | 0 | 0 | 0 | 1 | 0.55 | 0 | 1 | 2.62 |
| 5280489 | Low | No | Yes | No | No | No | No | No | 0.04 | 2 | 4 | 0 | 1 | 2 | 0.17 | 0 | 1 | 6.19 |
| 5280537 | High | No | No | No | No | No | Yes | Yes | -6.72 | 0 | 0 | 0 | 0 | 0 | 0.55 | 0 | 1 | 2.55 |
| 5280704 | Low | No | Yes | No | No | No | No | No | -7.65 | 1 | 0 | 1 | 1 | 2 | 0.55 | 0 | 0 | 5.12 |
| 5280794 | Low | No | No | No | No | Yes | No | No | -2.74 | 1 | 3 | 0 | 1 | 2 | 0.55 | 0 | 1 | 6.21 |
| 5280804 | Low | No | No | No | No | No | No | No | -8.88 | 2 | 1 | 1 | 1 | 3 | 0.17 | 1 | 1 | 5.32 |
| 5280805 | Low | No | Yes | No | No | No | No | No | -10.3 | 3 | 4 | 1 | 1 | 4 | 0.17 | 1 | 1 | 6.52 |
| 5280862 | High | No | No | Yes | No | No | Yes | Yes | -6.56 | 0 | 0 | 0 | 0 | 0 | 0.55 | 0 | 0 | 3.2 |
| 5280863 | High | No | No | Yes | No | No | Yes | Yes | -6.7 | 0 | 0 | 0 | 0 | 0 | 0.55 | 0 | 0 | 3.14 |
| 5280934 | High | Yes | No | Yes | No | Yes | No | No | -3.41 | 1 | 1 | 1 | 0 | 1 | 0.85 | 0 | 1 | 3.03 |
| 5281119 | High | Yes | No | Yes | No | Yes | No | No | -3.78 | 0 | 0 | 1 | 0 | 1 | 0.85 | 0 | 1 | 2.63 |
| 5281303 | Low | No | Yes | No | No | No | No | No | -9.92 | 2 | 3 | 1 | 1 | 4 | 0.17 | 0 | 3 | 8.11 |
| 5281310 | Low | No | Yes | No | No | No | No | No | -7.15 | 1 | 3 | 0 | 0 | 0 | 0.55 | 0 | 3 | 7.04 |
| 5281426 | High | Yes | No | Yes | No | No | No | No | -6.17 | 0 | 1 | 0 | 0 | 1 | 0.55 | 0 | 1 | 2.56 |
| 5281515 | Low | No | No | No | Yes | Yes | No | No | -4.44 | 1 | 0 | 0 | 0 | 1 | 0.55 | 0 | 1 | 4.51 |
| 5281520 | Low | No | No | No | No | Yes | No | No | -4.32 | 1 | 0 | 0 | 0 | 1 | 0.55 | 0 | 1 | 3.66 |
| 5281522 | Low | No | No | No | Yes | Yes | No | No | -4.44 | 1 | 0 | 0 | 0 | 1 | 0.55 | 0 | 1 | 4.51 |
| 5281553 | Low | Yes | No | No | No | No | No | No | -4.11 | 0 | 1 | 0 | 0 | 2 | 0.55 | 0 | 2 | 3.63 |
| 5281672 | Low | No | No | Yes | No | No | No | Yes | -7.4 | 1 | 0 | 1 | 1 | 2 | 0.55 | 1 | 1 | 3.27 |
| 5281876 | Low | No | Yes | No | No | No | No | No | -9.92 | 2 | 3 | 1 | 1 | 4 | 0.17 | 0 | 2 | 8.11 |
| 5282102 | Low | No | No | No | No | No | No | No | -8.52 | 2 | 0 | 1 | 1 | 3 | 0.17 | 0 | 0 | 5.29 |
| 5282822 | High | Yes | No | Yes | No | Yes | No | No | -3.41 | 1 | 1 | 1 | 0 | 1 | 0.85 | 0 | 1 | 3.03 |
| 5283640 | Low | No | No | No | No | No | No | No | -2.57 | 1 | 3 | 0 | 1 | 2 | 0.55 | 0 | 1 | 6.09 |
| 5284421 | High | No | No | Yes | No | Yes | No | No | -3.25 | 1 | 1 | 1 | 1 | 1 | 0.55 | 0 | 1 | 3.18 |
| 5288340 | Low | No | Yes | No | No | No | No | No | -11.6 | 2 | 1 | 1 | 1 | 4 | 0.17 | 0 | 0 | 5.3 |
| 5288377 | Low | No | Yes | No | No | No | No | No | -11.3 | 2 | 1 | 1 | 1 | 4 | 0.11 | 0 | 0 | 5.15 |
| 5315396 | High | No | No | No | No | Yes | No | No | -4.85 | 0 | 0 | 0 | 0 | 0 | 0.55 | 0 | 1 | 3.48 |
| 5315851 | Low | No | No | No | No | No | No | No | -8.33 | 0 | 0 | 1 | 1 | 1 | 0.55 | 1 | 0 | 4.97 |
| 5316525 | High | Yes | No | Yes | No | No | No | No | -5.23 | 0 | 0 | 0 | 0 | 0 | 0.55 | 0 | 2 | 2.84 |
| 5316800 | High | Yes | No | Yes | No | Yes | No | Yes | -4.95 | 0 | 0 | 0 | 0 | 0 | 0.55 | 1 | 0 | 2.46 |
| 5316860 | Low | No | No | No | No | No | No | No | -9.5 | 0 | 1 | 0 | 1 | 0 | 0.55 | 0 | 0 | 4.74 |
| 5316891 | High | Yes | No | Yes | No | No | No | No | -6.09 | 0 | 0 | 0 | 0 | 1 | 0.55 | 0 | 0 | 2.64 |
| 5318565 | High | Yes | No | Yes | No | No | No | No | -6.59 | 0 | 0 | 0 | 0 | 0 | 0.55 | 0 | 1 | 3.02 |
| 5318767 | Low | No | Yes | No | No | No | No | No | -9.91 | 3 | 4 | 1 | 1 | 3 | 0.17 | 0 | 0 | 6.48 |
| 5319336 | High | No | Yes | No | No | No | No | No | -6.14 | 0 | 0 | 0 | 0 | 0 | 0.55 | 0 | 1 | 5.85 |
| 5319406 | High | Yes | No | Yes | Yes | Yes | No | Yes | -4.6 | 0 | 0 | 0 | 0 | 1 | 0.55 | 0 | 2 | 3.67 |
| 5319500 | High | Yes | No | Yes | No | No | No | No | -6.88 | 0 | 0 | 0 | 0 | 0 | 0.55 | 0 | 0 | 2.84 |
| 5319706 | High | Yes | Yes | No | No | No | No | No | -3.62 | 1 | 1 | 1 | 0 | 1 | 0.55 | 0 | 1 | 3.1 |
| 5319765 | High | Yes | No | No | No | No | No | No | -5.65 | 0 | 2 | 0 | 0 | 2 | 0.55 | 0 | 1 | 3.43 |
| 5320171 | Low | No | Yes | No | No | No | No | No | -7.06 | 1 | 4 | 0 | 0 | 1 | 0.55 | 0 | 3 | 7.47 |
| 5320250 | Low | Yes | No | No | No | No | No | No | -4.11 | 0 | 1 | 0 | 0 | 2 | 0.55 | 0 | 2 | 3.63 |
| 5352019 | Low | No | Yes | No | No | No | No | No | -8.91 | 2 | 3 | 2 | 1 | 3 | 0.17 | 0 | 2 | 8.34 |
| 5353609 | High | Yes | No | No | No | No | No | No | -6.43 | 0 | 0 | 0 | 0 | 1 | 0.55 | 0 | 2 | 2.2 |
| 5363269 | Low | No | No | Yes | No | No | No | No | -2.49 | 1 | 1 | 1 | 1 | 2 | 0.55 | 0 | 1 | 3.34 |
| 5367462 | High | Yes | No | Yes | No | Yes | No | No | -3.62 | 1 | 1 | 1 | 0 | 1 | 0.55 | 0 | 1 | 3.1 |
| 5459840 | High | No | Yes | No | No | No | No | No | -8.91 | 1 | 2 | 0 | 1 | 1 | 0.55 | 0 | 0 | 6.36 |
| 5742590 | Low | No | No | No | No | No | No | No | -4.32 | 1 | 4 | 0 | 0 | 1 | 0.55 | 0 | 1 | 8.02 |
| 6324923 | Low | No | Yes | No | No | No | No | No | -7.94 | 0 | 0 | 1 | 1 | 1 | 0.55 | 1 | 0 | 4.96 |
| 6434062 | Low | Yes | No | No | No | No | No | No | -4.21 | 0 | 1 | 0 | 0 | 2 | 0.55 | 0 | 1 | 4 |
| 6437066 | Low | No | Yes | No | No | No | No | No | -7.15 | 1 | 3 | 0 | 0 | 0 | 0.55 | 0 | 3 | 7.04 |
| 6442484 | Low | No | Yes | No | No | No | No | No | -6.46 | 1 | 4 | 0 | 1 | 2 | 0.55 | 0 | 3 | 7.75 |
| 6442906 | High | No | Yes | No | No | No | No | No | -7.75 | 1 | 2 | 0 | 0 | 0 | 0.55 | 0 | 3 | 6.67 |
| 6450230 | High | No | No | No | No | No | No | No | -6.33 | 0 | 0 | 0 | 0 | 0 | 0.55 | 0 | 2 | 4.02 |
| 6451151 | Low | No | Yes | No | No | No | No | No | -10.4 | 0 | 1 | 1 | 1 | 2 | 0.55 | 0 | 3 | 5.06 |
| 6451598 | High | Yes | No | No | Yes | Yes | Yes | No | -4.37 | 0 | 0 | 0 | 0 | 1 | 0.55 | 0 | 0 | 3.3 |
| 6453932 | Low | No | Yes | No | No | No | No | No | -7.78 | 3 | 3 | 1 | 1 | 4 | 0.11 | 0 | 2 | 8.72 |
| 6482976 | High | Yes | No | No | No | No | Yes | No | -6.53 | 0 | 0 | 0 | 0 | 0 | 0.55 | 0 | 0 | 2.11 |
| 6918743 | Low | No | Yes | No | No | No | No | No | -12.3 | 2 | 1 | 1 | 1 | 4 | 0.17 | 0 | 3 | 5.72 |
| 9548705 | Low | No | No | No | No | Yes | No | No | -3.38 | 1 | 0 | 0 | 0 | 2 | 0.55 | 0 | 1 | 4.1 |
| 9796891 | High | Yes | No | Yes | Yes | Yes | Yes | Yes | -5.52 | 0 | 0 | 0 | 0 | 0 | 0.55 | 0 | 3 | 3.92 |
| 10031185 | Low | No | Yes | No | No | No | No | No | -9.57 | 3 | 3 | 1 | 1 | 3 | 0.17 | 0 | 0 | 6.19 |
| 10065647 | High | Yes | No | Yes | Yes | Yes | No | Yes | -7.34 | 0 | 0 | 0 | 0 | 0 | 0.55 | 0 | 0 | 3.73 |
| 10098738 | Low | No | Yes | No | No | No | No | No | -10.2 | 2 | 3 | 1 | 1 | 2 | 0.17 | 0 | 1 | 6.44 |
| 10108651 | High | Yes | No | Yes | No | No | Yes | Yes | -6.38 | 0 | 0 | 0 | 0 | 0 | 0.55 | 0 | 1 | 2.85 |
| 10181133 | High | No | No | No | No | No | No | No | -4.96 | 1 | 2 | 0 | 0 | 1 | 0.55 | 0 | 1 | 6.53 |
| 10212035 | Low | No | Yes | No | No | No | No | No | 0.76 | 2 | 4 | 1 | 1 | 3 | 0.17 | 0 | 0 | 7.17 |
| 10239837 | Low | No | No | No | No | No | No | Yes | -7.8 | 3 | 2 | 1 | 1 | 3 | 0.17 | 1 | 1 | 5.28 |
| 10263440 | High | Yes | No | No | No | No | No | No | -6.07 | 0 | 0 | 0 | 0 | 0 | 0.55 | 0 | 1 | 4.04 |
| 10348278 | Low | No | Yes | No | No | No | No | No | -9.57 | 3 | 3 | 1 | 1 | 3 | 0.17 | 0 | 0 | 6.19 |
| 10392456 | Low | No | Yes | No | No | No | No | No | -10.2 | 2 | 3 | 1 | 1 | 2 | 0.17 | 0 | 1 | 6.44 |
| 10438246 | Low | No | Yes | No | No | No | No | No | -9.57 | 3 | 3 | 1 | 1 | 3 | 0.17 | 0 | 0 | 6.19 |
| 10442609 | High | Yes | No | No | No | No | No | No | -6.01 | 0 | 0 | 0 | 0 | 1 | 0.55 | 0 | 1 | 2.84 |
| 10483388 | Low | No | Yes | No | No | No | No | No | -9.57 | 3 | 3 | 1 | 1 | 3 | 0.17 | 0 | 0 | 6.19 |
| 10505484 | High | No | No | No | No | No | No | Yes | -8.13 | 0 | 1 | 0 | 0 | 0 | 0.55 | 0 | 2 | 6.32 |
| 10601920 | High | No | Yes | No | No | No | No | No | -4.48 | 2 | 4 | 0 | 1 | 1 | 0.17 | 0 | 1 | 5.84 |
| 10628287 | Low | No | Yes | No | No | No | No | No | -8.97 | 2 | 3 | 1 | 1 | 4 | 0.17 | 0 | 3 | 7.96 |
| 10767085 | High | No | Yes | No | No | No | No | No | -8.91 | 1 | 2 | 0 | 1 | 1 | 0.55 | 0 | 0 | 6.36 |
| 10884852 | Low | No | No | No | No | No | No | No | -8.85 | 0 | 0 | 1 | 1 | 0 | 0.55 | 0 | 0 | 5.15 |
| 10906239 | High | No | No | No | No | Yes | No | No | -4.9 | 1 | 1 | 0 | 1 | 1 | 0.55 | 0 | 1 | 5.87 |
| 10977864 | High | No | No | No | No | No | No | No | -7.6 | 0 | 0 | 0 | 0 | 0 | 0.55 | 0 | 0 | 3.45 |
| 11077057 | High | Yes | No | Yes | Yes | Yes | Yes | No | -5.64 | 0 | 0 | 0 | 0 | 0 | 0.55 | 0 | 0 | 2.46 |
| 11087935 | High | Yes | No | No | No | No | Yes | No | -7.73 | 0 | 0 | 0 | 0 | 0 | 0.55 | 0 | 1 | 4.45 |
| 11088324 | High | Yes | No | Yes | Yes | Yes | Yes | Yes | -5.52 | 0 | 0 | 0 | 0 | 0 | 0.55 | 0 | 3 | 3.92 |
| 11095397 | High | No | No | Yes | No | No | No | No | -6.51 | 0 | 0 | 0 | 0 | 1 | 0.55 | 0 | 3 | 1.89 |
| 11119228 | High | Yes | No | No | Yes | Yes | Yes | No | -4.51 | 0 | 0 | 0 | 0 | 0 | 0.55 | 0 | 0 | 3.16 |
| 11192900 | Low | No | Yes | No | No | No | No | Yes | -6.92 | 1 | 3 | 1 | 1 | 2 | 0.55 | 0 | 2 | 7.4 |
| 11209134 | High | Yes | No | Yes | Yes | Yes | Yes | Yes | -6.08 | 0 | 0 | 0 | 0 | 0 | 0.55 | 0 | 1 | 3.02 |
| 11243273 | High | Yes | No | No | Yes | No | No | No | -6.1 | 0 | 0 | 0 | 0 | 0 | 0.55 | 0 | 0 | 3.14 |
| 11334829 | High | Yes | Yes | No | No | No | Yes | No | -5.03 | 0 | 0 | 0 | 0 | 0 | 0.55 | 1 | 1 | 3.13 |
| 11403749 | High | Yes | No | Yes | No | No | Yes | Yes | -6.38 | 0 | 0 | 0 | 0 | 0 | 0.55 | 0 | 1 | 2.85 |
| 11482406 | High | No | No | No | No | No | No | No | -6.94 | 0 | 0 | 0 | 0 | 0 | 0.55 | 0 | 1 | 3.68 |
| 11767849 | Low | No | Yes | No | No | No | No | No | -3.1 | 2 | 4 | 2 | 2 | 5 | 0.17 | 0 | 1 | 8.76 |
| 11770062 | High | Yes | No | No | Yes | Yes | No | No | -5.03 | 0 | 0 | 0 | 0 | 1 | 0.55 | 0 | 1 | 4.34 |
| 11798426 | High | No | Yes | No | No | Yes | No | No | -6.26 | 0 | 0 | 0 | 0 | 0 | 0.55 | 0 | 1 | 6.11 |
| 11813223 | Low | No | Yes | No | No | No | No | No | -9.24 | 3 | 1 | 1 | 1 | 3 | 0.17 | 0 | 2 | 6.1 |
| 11818411 | High | Yes | No | No | No | No | Yes | No | -6.53 | 0 | 0 | 0 | 0 | 0 | 0.55 | 0 | 1 | 2.76 |
| 11870456 | Low | No | No | No | No | No | No | No | -2.2 | 1 | 3 | 0 | 1 | 2 | 0.55 | 0 | 1 | 6.3 |
| 12004512 | High | No | Yes | No | No | No | No | No | -6.25 | 0 | 1 | 0 | 0 | 0 | 0.55 | 0 | 2 | 6.48 |
| 12011153 | High | No | Yes | No | No | No | No | Yes | -6.66 | 0 | 0 | 0 | 0 | 0 | 0.55 | 0 | 0 | 5.88 |
| 12046149 | High | Yes | No | No | Yes | Yes | No | No | -5.03 | 0 | 0 | 0 | 0 | 1 | 0.55 | 0 | 1 | 4.34 |
| 12069125 | High | No | Yes | No | No | No | No | No | -6.55 | 0 | 0 | 0 | 0 | 0 | 0.55 | 0 | 0 | 5.8 |
| 12072821 | High | No | Yes | No | No | Yes | No | No | -5.73 | 0 | 3 | 0 | 0 | 0 | 0.55 | 0 | 2 | 6.41 |
| 12272224 | High | Yes | No | Yes | No | No | No | No | -5.95 | 0 | 0 | 0 | 0 | 0 | 0.55 | 0 | 0 | 2.7 |
| 12302222 | High | Yes | No | No | Yes | No | No | No | -5.29 | 0 | 0 | 0 | 0 | 1 | 0.55 | 0 | 1 | 4.29 |
| 12302226 | High | Yes | No | No | Yes | No | No | No | -5.29 | 0 | 0 | 0 | 0 | 1 | 0.55 | 0 | 1 | 4.29 |
| 12302227 | High | Yes | No | No | Yes | No | No | No | -5.29 | 0 | 0 | 0 | 0 | 1 | 0.55 | 0 | 1 | 4.29 |
| 12302228 | High | Yes | No | No | Yes | No | No | No | -5.29 | 0 | 0 | 0 | 0 | 1 | 0.55 | 0 | 1 | 4.29 |
| 12302243 | Low | No | No | No | No | No | Yes | No | -4.39 | 1 | 0 | 0 | 0 | 1 | 0.55 | 0 | 0 | 3.22 |
| 12303902 | Low | Yes | No | Yes | Yes | Yes | No | No | -4.37 | 1 | 0 | 0 | 0 | 1 | 0.55 | 0 | 1 | 4.62 |
| 12305246 | Low | Yes | No | Yes | Yes | Yes | No | No | -4.2 | 1 | 0 | 0 | 0 | 1 | 0.55 | 0 | 1 | 3.7 |
| 12305247 | Low | Yes | No | Yes | Yes | Yes | No | No | -4.2 | 1 | 0 | 0 | 0 | 1 | 0.55 | 0 | 1 | 3.7 |
| 12306053 | Low | No | No | No | Yes | Yes | No | No | -4.65 | 1 | 0 | 0 | 0 | 1 | 0.55 | 0 | 1 | 4.35 |
| 12308714 | High | No | Yes | No | No | No | No | No | -5.63 | 0 | 0 | 0 | 0 | 0 | 0.55 | 0 | 0 | 5.89 |
| 12308716 | High | No | Yes | No | No | No | No | No | -5.63 | 0 | 0 | 0 | 0 | 0 | 0.55 | 0 | 0 | 5.89 |
| 12309449 | Low | No | No | No | Yes | Yes | No | No | -3.8 | 1 | 0 | 0 | 0 | 2 | 0.55 | 0 | 1 | 4.25 |
| 12309491 | High | No | Yes | No | No | No | No | No | -7.46 | 0 | 0 | 0 | 0 | 0 | 0.55 | 0 | 0 | 3.39 |
| 12310089 | Low | No | No | No | No | No | No | No | -8.33 | 1 | 0 | 1 | 1 | 2 | 0.55 | 0 | 0 | 5.12 |
| 12312690 | High | No | Yes | No | No | No | No | No | -8.68 | 1 | 3 | 0 | 1 | 1 | 0.55 | 0 | 0 | 6.54 |
| 12313023 | Low | No | No | No | Yes | Yes | No | No | -4.49 | 1 | 0 | 0 | 0 | 1 | 0.55 | 0 | 1 | 4.35 |
| 12313376 | High | No | Yes | No | No | No | No | No | -7.61 | 0 | 0 | 0 | 0 | 0 | 0.55 | 0 | 2 | 6.07 |
| 12376292 | High | Yes | No | No | No | No | No | No | -5.94 | 0 | 3 | 0 | 0 | 2 | 0.55 | 0 | 2 | 1.92 |
| 12443210 | Low | No | Yes | No | No | No | No | No | -8.73 | 2 | 0 | 1 | 1 | 3 | 0.17 | 0 | 0 | 5.44 |
| 12677834 | High | Yes | No | No | No | Yes | No | No | -5.59 | 0 | 0 | 0 | 0 | 1 | 0.55 | 0 | 1 | 3.82 |
| 13370049 | High | Yes | Yes | No | No | Yes | No | No | -5.08 | 0 | 0 | 0 | 0 | 0 | 0.85 | 0 | 0 | 3.53 |
| 13458955 | Low | No | No | No | No | No | No | No | -1.86 | 1 | 3 | 0 | 1 | 2 | 0.55 | 0 | 0 | 5.42 |
| 13821181 | High | Yes | No | Yes | Yes | Yes | Yes | Yes | -5.46 | 0 | 0 | 0 | 0 | 0 | 0.55 | 0 | 1 | 2.87 |
| 13834020 | High | No | Yes | No | No | No | No | No | -5.7 | 0 | 0 | 0 | 0 | 0 | 0.55 | 0 | 1 | 6.04 |
| 13856086 | High | No | Yes | No | No | No | No | No | -7.69 | 1 | 3 | 0 | 1 | 0 | 0.55 | 0 | 1 | 6.53 |
| 13856092 | High | No | Yes | No | No | No | No | No | -6.44 | 0 | 0 | 0 | 0 | 0 | 0.56 | 0 | 0 | 5.76 |
| 13858079 | Low | No | Yes | No | No | No | No | No | -9.08 | 2 | 1 | 1 | 1 | 2 | 0.17 | 0 | 2 | 6.63 |
| 13875741 | High | Yes | Yes | No | No | No | No | No | -5.77 | 0 | 0 | 0 | 0 | 0 | 0.55 | 0 | 0 | 5.71 |
| 13875755 | High | No | Yes | No | No | No | No | No | -6.82 | 0 | 2 | 0 | 0 | 0 | 0.55 | 0 | 2 | 6.37 |
| 13875766 | Low | No | No | No | No | No | No | No | -8.36 | 1 | 3 | 1 | 1 | 1 | 0.55 | 0 | 2 | 7 |
| 13875774 | Low | No | Yes | No | No | No | No | No | -8.03 | 1 | 3 | 1 | 1 | 1 | 0.55 | 0 | 2 | 7.01 |
| 13875775 | High | No | Yes | No | No | No | No | No | -8.46 | 0 | 1 | 0 | 0 | 0 | 0.55 | 0 | 2 | 6.37 |
| 13965525 | High | Yes | No | Yes | Yes | Yes | No | Yes | -5.3 | 0 | 0 | 0 | 0 | 0 | 0.55 | 0 | 2 | 3.88 |
| 14015932 | Low | No | Yes | No | No | No | No | No | -7.05 | 3 | 4 | 1 | 1 | 6 | 0.17 | 0 | 0 | 6.61 |
| 14015943 | Low | No | No | No | No | No | No | Yes | -7.45 | 2 | 2 | 1 | 1 | 2 | 0.17 | 0 | 0 | 5.19 |
| 14015944 | Low | No | No | No | No | No | No | Yes | -7.45 | 2 | 2 | 1 | 1 | 2 | 0.17 | 0 | 0 | 5.19 |
| 14015948 | Low | No | No | No | No | No | No | Yes | -7.8 | 3 | 2 | 1 | 1 | 3 | 0.17 | 1 | 1 | 5.28 |
| 14015949 | Low | No | No | No | No | No | No | Yes | -7.8 | 3 | 2 | 1 | 1 | 3 | 0.17 | 1 | 1 | 5.28 |
| 14015956 | Low | No | No | No | No | No | No | No | -8.83 | 1 | 1 | 1 | 1 | 2 | 0.55 | 0 | 0 | 5.27 |
| 14015959 | Low | No | No | No | No | No | No | Yes | -7.8 | 3 | 2 | 1 | 1 | 3 | 0.17 | 1 | 1 | 5.28 |
| 14015964 | Low | No | No | No | No | No | No | Yes | -6.31 | 2 | 2 | 0 | 1 | 1 | 0.17 | 0 | 0 | 4.99 |
| 14015965 | Low | No | No | No | No | No | No | Yes | -6.31 | 2 | 2 | 0 | 1 | 1 | 0.17 | 0 | 0 | 4.99 |
| 14015967 | Low | No | No | No | No | No | No | Yes | -6.31 | 2 | 2 | 0 | 1 | 1 | 0.17 | 0 | 0 | 4.96 |
| 14015968 | Low | No | No | No | No | No | No | Yes | -6.31 | 2 | 2 | 0 | 1 | 1 | 0.17 | 0 | 0 | 4.96 |
| 14136864 | Low | No | No | No | No | No | No | No | -9.43 | 2 | 3 | 1 | 1 | 2 | 0.17 | 0 | 2 | 6.81 |
| 14194023 | High | No | No | No | No | No | No | No | -8.07 | 1 | 3 | 0 | 0 | 0 | 0.55 | 0 | 3 | 6.33 |
| 14194109 | Low | No | Yes | No | No | No | No | No | -9.13 | 0 | 1 | 1 | 1 | 1 | 0.55 | 0 | 0 | 6.32 |
| 14213968 | High | Yes | No | Yes | No | No | No | No | -6.33 | 0 | 0 | 0 | 0 | 1 | 0.55 | 0 | 2 | 2.29 |
| 14218028 | High | No | No | Yes | No | Yes | Yes | Yes | -5.22 | 0 | 0 | 0 | 0 | 0 | 0.55 | 0 | 1 | 3.67 |
| 14287157 | High | Yes | No | No | Yes | Yes | Yes | No | -4.14 | 0 | 0 | 0 | 0 | 1 | 0.55 | 0 | 0 | 3.48 |
| 14287159 | High | Yes | No | No | Yes | No | Yes | Yes | -4.68 | 0 | 0 | 0 | 0 | 0 | 0.55 | 0 | 0 | 3.32 |
| 14309784 | High | No | Yes | No | No | No | No | No | -6.9 | 0 | 0 | 0 | 0 | 0 | 0.55 | 0 | 2 | 4.33 |
| 14458886 | High | No | Yes | No | No | No | No | No | -7.3 | 1 | 3 | 0 | 0 | 0 | 0.55 | 0 | 3 | 6.86 |
| 14467538 | High | Yes | No | No | No | No | Yes | No | -7.34 | 0 | 0 | 0 | 0 | 0 | 0.55 | 0 | 1 | 6.05 |
| 14492795 | High | No | No | No | No | Yes | No | Yes | -4.21 | 0 | 0 | 0 | 0 | 1 | 0.55 | 0 | 1 | 4.32 |
| 14563366 | Low | No | Yes | No | No | Yes | No | No | -6.54 | 1 | 4 | 0 | 1 | 2 | 0.55 | 0 | 3 | 7.63 |
| 14752824 | High | No | No | No | Yes | No | Yes | No | -7.08 | 0 | 0 | 1 | 0 | 0 | 0.55 | 0 | 2 | 3.01 |
| 14807789 | High | Yes | No | Yes | No | No | No | No | -5.48 | 0 | 0 | 0 | 0 | 1 | 0.55 | 0 | 0 | 2.78 |
| 14845542 | Low | No | No | No | No | No | No | No | -2.24 | 1 | 3 | 0 | 1 | 2 | 0.55 | 0 | 1 | 6.21 |
| 14845550 | High | No | Yes | No | No | No | No | Yes | -5.37 | 2 | 4 | 0 | 1 | 1 | 0.17 | 0 | 1 | 6.51 |
| 15008366 | High | Yes | No | No | No | Yes | Yes | Yes | -6.04 | 0 | 0 | 0 | 0 | 0 | 0.55 | 0 | 2 | 4.13 |
| 15108321 | High | No | Yes | No | No | No | No | No | -9.2 | 0 | 1 | 0 | 0 | 0 | 0.55 | 0 | 0 | 4.88 |
| 15215479 | High | Yes | Yes | No | No | No | No | No | -7.01 | 0 | 0 | 0 | 0 | 0 | 0.55 | 0 | 0 | 4.91 |
| 15485379 | High | No | Yes | No | No | No | No | No | -6.37 | 0 | 0 | 0 | 0 | 0 | 0.55 | 0 | 1 | 6.52 |
| 15560114 | High | No | No | No | No | No | No | No | -6.33 | 0 | 0 | 0 | 0 | 0 | 0.55 | 0 | 2 | 4.02 |
| 15560276 | Low | No | No | No | Yes | Yes | No | No | -4.64 | 1 | 0 | 0 | 0 | 1 | 0.55 | 0 | 1 | 4.57 |
| 15560423 | Low | No | No | No | No | Yes | No | No | -4.17 | 1 | 3 | 0 | 1 | 1 | 0.55 | 0 | 1 | 5.86 |
| 15768008 | Low | No | No | No | No | No | Yes | No | -9.96 | 2 | 3 | 2 | 1 | 3 | 0.17 | 0 | 1 | 7.27 |
| 15768009 | Low | No | No | No | No | No | Yes | No | -9.96 | 2 | 3 | 2 | 1 | 3 | 0.17 | 0 | 1 | 7.27 |
| 15840160 | High | No | Yes | No | No | No | No | Yes | -5.08 | 1 | 4 | 0 | 0 | 1 | 0.55 | 0 | 1 | 5.97 |
| 15885442 | High | No | Yes | No | No | Yes | No | No | -4.95 | 2 | 4 | 0 | 1 | 1 | 0.17 | 0 | 2 | 6.55 |
| 15885443 | High | No | Yes | No | No | Yes | No | Yes | -5.1 | 1 | 1 | 0 | 0 | 1 | 0.56 | 0 | 2 | 5.92 |
| 15934443 | High | Yes | No | No | Yes | Yes | No | Yes | -4.77 | 0 | 0 | 0 | 0 | 0 | 0.55 | 0 | 0 | 3.38 |
| 16126804 | Low | No | Yes | No | No | No | No | No | -9.92 | 2 | 3 | 1 | 1 | 4 | 0.17 | 0 | 3 | 8.11 |
| 16396350 | Low | No | No | No | Yes | Yes | No | No | -3.94 | 1 | 0 | 0 | 0 | 2 | 0.55 | 0 | 1 | 3.6 |
| 16722121 | Low | No | Yes | No | No | No | No | No | -9.36 | 2 | 3 | 1 | 1 | 4 | 0.17 | 0 | 3 | 7.87 |
| 16722130 | Low | No | Yes | No | No | No | No | No | -8.41 | 2 | 3 | 1 | 1 | 4 | 0.17 | 0 | 3 | 7.74 |
| 20056138 | High | No | Yes | No | No | No | No | No | -7.8 | 0 | 3 | 0 | 0 | 0 | 0.55 | 0 | 1 | 6.28 |
| 20488062 | High | Yes | No | Yes | No | No | No | No | -5.84 | 0 | 3 | 0 | 0 | 2 | 0.85 | 0 | 0 | 1 |
| 20976991 | Low | No | Yes | No | No | No | No | No | 3.03 | 2 | 4 | 1 | 1 | 2 | 0.17 | 0 | 0 | 5.16 |
| 21581301 | Low | No | Yes | No | No | No | No | No | -9.37 | 2 | 3 | 1 | 1 | 4 | 0.17 | 0 | 3 | 7.79 |
| 21581584 | Low | No | Yes | No | No | No | No | No | -7.29 | 1 | 3 | 0 | 0 | 1 | 0.55 | 0 | 3 | 7.14 |
| 21592304 | High | No | Yes | No | No | No | No | No | -6.55 | 0 | 0 | 0 | 0 | 0 | 0.55 | 0 | 0 | 5.62 |
| 21594203 | High | No | Yes | No | No | No | No | No | -4.82 | 1 | 3 | 0 | 1 | 1 | 0.56 | 0 | 1 | 6.35 |
| 21597549 | High | No | No | No | No | No | No | No | -8.7 | 0 | 0 | 0 | 0 | 0 | 0.55 | 0 | 1 | 6.05 |
| 21600035 | Low | No | Yes | No | No | No | No | Yes | -5.75 | 2 | 4 | 0 | 1 | 2 | 0.17 | 0 | 2 | 7.49 |
| 21603566 | High | No | Yes | No | No | No | No | No | -4.82 | 1 | 3 | 0 | 1 | 1 | 0.56 | 0 | 1 | 6.35 |
| 21625636 | Low | No | Yes | No | No | No | No | No | -8.73 | 2 | 2 | 1 | 1 | 2 | 0.17 | 0 | 1 | 6.58 |
| 21626436 | Low | No | Yes | No | No | No | No | No | -8.18 | 3 | 3 | 1 | 1 | 5 | 0.17 | 0 | 0 | 6.77 |
| 21632833 | High | Yes | Yes | No | Yes | No | No | Yes | -5.19 | 0 | 0 | 0 | 0 | 0 | 0.55 | 0 | 0 | 3.33 |
| 21632843 | High | Yes | No | No | No | No | Yes | No | -4.37 | 0 | 0 | 0 | 0 | 1 | 0.55 | 0 | 0 | 3.57 |
| 21725519 | Low | No | Yes | No | No | No | No | No | -10.2 | 2 | 3 | 2 | 1 | 4 | 0.17 | 0 | 3 | 8.15 |
| 21725521 | Low | No | Yes | No | No | No | No | No | -9.43 | 2 | 3 | 1 | 1 | 4 | 0.17 | 0 | 3 | 7.86 |
| 21725522 | Low | No | Yes | No | No | No | No | No | -9.62 | 2 | 3 | 1 | 1 | 4 | 0.17 | 0 | 2 | 8.24 |
| 23256847 | Low | No | Yes | No | No | No | No | No | -9.8 | 2 | 4 | 1 | 1 | 3 | 0.17 | 0 | 2 | 7.4 |
| 24796982 | Low | No | Yes | No | No | No | No | No | -10.2 | 2 | 3 | 2 | 1 | 4 | 0.17 | 0 | 3 | 8.15 |
| 24867638 | High | Yes | No | Yes | No | No | No | No | -6.44 | 0 | 0 | 0 | 0 | 0 | 0.55 | 1 | 0 | 3.84 |
| 24879663 | High | No | No | No | No | No | No | No | -7.9 | 0 | 1 | 0 | 0 | 0 | 0.55 | 0 | 2 | 4.66 |
| 25769005 | High | No | No | No | No | No | No | No | -6.26 | 0 | 0 | 0 | 0 | 0 | 0.56 | 0 | 0 | 2.87 |
| 40469553 | High | No | Yes | No | No | No | No | No | -6.4 | 0 | 0 | 0 | 0 | 0 | 0.55 | 0 | 1 | 6.29 |
| 40469561 | High | No | Yes | No | No | No | No | No | -6.25 | 0 | 1 | 0 | 0 | 0 | 0.55 | 0 | 2 | 6.48 |
| 42433469 | High | Yes | No | No | Yes | No | No | No | -5 | 0 | 0 | 0 | 0 | 1 | 0.55 | 0 | 0 | 3.58 |
| 42607958 | High | No | No | No | No | No | No | Yes | -5.3 | 0 | 0 | 0 | 0 | 1 | 0.55 | 0 | 1 | 4.45 |
| 42608071 | High | Yes | No | Yes | Yes | Yes | No | Yes | -5.17 | 0 | 0 | 0 | 0 | 0 | 0.55 | 0 | 1 | 3.78 |
| 42608075 | High | Yes | No | No | Yes | Yes | No | Yes | -5.02 | 0 | 0 | 0 | 0 | 0 | 0.55 | 0 | 1 | 3.89 |
| 42608116 | High | No | No | Yes | No | Yes | Yes | Yes | -5.66 | 0 | 0 | 0 | 0 | 0 | 0.55 | 1 | 3 | 3.75 |
| 42626428 | High | Yes | No | No | No | No | No | No | -5.96 | 0 | 1 | 0 | 0 | 2 | 0.55 | 0 | 1 | 3.02 |
| 44146779 | High | No | No | No | No | No | No | No | -6.79 | 0 | 0 | 0 | 0 | 0 | 0.55 | 0 | 1 | 3.49 |
| 44259428 | Low | No | Yes | No | No | No | No | No | -10.6 | 3 | 4 | 1 | 1 | 4 | 0.17 | 1 | 1 | 6.56 |
| 44566526 | High | No | Yes | No | No | No | No | No | -6.41 | 1 | 3 | 0 | 0 | 0 | 0.55 | 0 | 2 | 6.51 |
| 44567124 | Low | No | No | No | No | No | No | No | -3.63 | 1 | 3 | 0 | 1 | 1 | 0.55 | 0 | 1 | 6.05 |
| 44567142 | High | No | Yes | No | No | No | No | Yes | -6.69 | 1 | 3 | 0 | 0 | 0 | 0.55 | 0 | 1 | 6.72 |
| 44575502 | Low | No | Yes | No | No | No | No | No | -12.7 | 2 | 1 | 1 | 1 | 4 | 0.17 | 0 | 0 | 5.88 |
| 44575793 | High | No | No | No | No | No | No | No | -5.13 | 1 | 3 | 0 | 1 | 1 | 0.55 | 0 | 2 | 6.58 |
| 44579695 | High | Yes | No | Yes | Yes | Yes | No | Yes | -4.73 | 0 | 0 | 0 | 0 | 1 | 0.55 | 0 | 2 | 3.27 |
| 44579696 | High | Yes | No | Yes | Yes | Yes | Yes | Yes | -4.76 | 0 | 0 | 0 | 0 | 1 | 0.55 | 0 | 2 | 2.82 |
| 44579743 | High | No | Yes | No | No | No | No | No | -8.5 | 0 | 0 | 0 | 1 | 0 | 0.55 | 0 | 1 | 5.12 |
| 44579744 | High | No | Yes | No | No | No | No | No | -8.5 | 0 | 0 | 0 | 1 | 0 | 0.55 | 0 | 1 | 5.12 |
| 44583637 | High | No | No | No | No | Yes | No | Yes | -6.3 | 0 | 0 | 0 | 0 | 0 | 0.55 | 1 | 0 | 2.74 |
| 44584063 | Low | No | Yes | No | No | No | No | No | -9.92 | 2 | 3 | 1 | 1 | 4 | 0.17 | 0 | 3 | 8.11 |
| 44614139 | High | No | Yes | No | No | No | No | No | -6.13 | 1 | 3 | 0 | 0 | 0 | 0.55 | 0 | 1 | 6.79 |
| 44631202 | High | No | Yes | No | No | No | No | No | -7.61 | 0 | 0 | 0 | 0 | 0 | 0.55 | 0 | 2 | 6.07 |
| 44715635 | High | No | No | No | No | No | Yes | Yes | -7.8 | 0 | 1 | 0 | 0 | 0 | 0.55 | 0 | 2 | 6.21 |
| 45103626 | High | Yes | No | Yes | Yes | Yes | No | Yes | -4.54 | 0 | 0 | 0 | 0 | 1 | 0.55 | 1 | 0 | 3.3 |
| 45268397 | High | No | No | No | No | Yes | No | Yes | -4.21 | 0 | 0 | 0 | 0 | 1 | 0.55 | 0 | 1 | 4.32 |
| 45272307 | High | Yes | No | No | No | No | No | No | -6.61 | 0 | 0 | 0 | 0 | 0 | 0.55 | 0 | 0 | 3.31 |
| 46173826 | High | No | Yes | No | No | No | No | No | -5.74 | 0 | 3 | 0 | 0 | 1 | 0.55 | 0 | 1 | 6.96 |
| 46201020 | High | Yes | No | Yes | Yes | Yes | No | Yes | -4.83 | 0 | 0 | 0 | 0 | 0 | 0.55 | 1 | 1 | 2.95 |
| 46211187 | High | No | No | No | No | No | Yes | Yes | -6 | 0 | 0 | 0 | 0 | 0 | 0.55 | 0 | 0 | 4.4 |
| 46224590 | Low | No | Yes | No | No | No | No | No | -10.5 | 1 | 1 | 1 | 1 | 2 | 0.55 | 0 | 0 | 5.34 |
| 46919586 | High | No | Yes | No | No | No | No | No | -5.78 | 1 | 3 | 0 | 0 | 1 | 0.55 | 0 | 2 | 6.93 |
| 49863985 | High | No | Yes | No | No | No | No | No | -6.14 | 0 | 0 | 0 | 0 | 0 | 0.55 | 0 | 1 | 6.34 |
| 49864004 | High | No | Yes | No | No | No | No | No | -6.46 | 0 | 1 | 0 | 0 | 0 | 0.55 | 0 | 0 | 6.26 |
| 49864005 | High | No | Yes | No | No | No | No | Yes | -6.38 | 1 | 3 | 0 | 0 | 0 | 0.55 | 0 | 1 | 6.97 |
| 49864006 | High | No | Yes | No | No | No | No | No | -6.4 | 1 | 3 | 0 | 0 | 0 | 0.55 | 0 | 2 | 6.56 |
| 51402807 | Low | No | No | No | No | No | No | No | -8.88 | 2 | 1 | 1 | 1 | 3 | 0.17 | 1 | 1 | 5.32 |
| 51694242 | High | Yes | Yes | No | No | No | Yes | No | -6.07 | 0 | 0 | 0 | 0 | 0 | 0.55 | 0 | 0 | 3.57 |
| 52951756 | High | No | Yes | No | No | No | No | Yes | -5.82 | 0 | 0 | 0 | 0 | 0 | 0.55 | 0 | 0 | 5.94 |
| 52951892 | High | No | Yes | No | No | No | No | Yes | -6.39 | 0 | 0 | 0 | 0 | 0 | 0.55 | 0 | 0 | 5.98 |
| 52951893 | High | No | Yes | No | No | Yes | No | No | -5.34 | 1 | 4 | 0 | 1 | 1 | 0.55 | 0 | 1 | 6.52 |
| 52951894 | High | No | Yes | No | No | No | No | Yes | -6.39 | 0 | 0 | 0 | 0 | 0 | 0.55 | 0 | 0 | 5.98 |
| 52951895 | High | No | Yes | No | No | No | No | Yes | -5.81 | 0 | 0 | 0 | 0 | 0 | 0.55 | 0 | 0 | 6.14 |
| 52952011 | High | No | Yes | No | No | Yes | No | No | -5.51 | 1 | 1 | 0 | 0 | 1 | 0.55 | 0 | 2 | 5.96 |
| 52952012 | High | Yes | No | No | No | Yes | No | No | -5.76 | 0 | 0 | 0 | 0 | 0 | 0.55 | 0 | 0 | 5.41 |
| 52952013 | High | No | Yes | No | No | No | No | No | -6.41 | 1 | 3 | 0 | 0 | 0 | 0.55 | 0 | 2 | 6.51 |
| 52952112 | High | No | Yes | No | No | No | No | No | -5.45 | 1 | 3 | 0 | 0 | 1 | 0.55 | 0 | 2 | 6.69 |
| 52952113 | High | No | Yes | No | No | No | No | Yes | -6.66 | 0 | 0 | 0 | 0 | 0 | 0.55 | 0 | 0 | 5.88 |
| 52952216 | High | No | No | No | No | No | No | Yes | -8.32 | 0 | 1 | 0 | 0 | 0 | 0.55 | 0 | 2 | 6.28 |
| 52952322 | Low | No | Yes | No | No | Yes | No | No | -6.81 | 1 | 4 | 0 | 1 | 1 | 0.55 | 0 | 3 | 7.2 |
| 52952323 | Low | No | Yes | No | No | No | No | Yes | -7.01 | 1 | 3 | 0 | 0 | 0 | 0.55 | 0 | 2 | 7.17 |
| 52952435 | High | No | Yes | No | No | No | No | No | -7.72 | 1 | 3 | 0 | 0 | 0 | 0.55 | 0 | 2 | 6.66 |
| 52952436 | High | Yes | Yes | No | No | No | No | No | -6.34 | 0 | 0 | 0 | 0 | 0 | 0.55 | 0 | 1 | 5.43 |
| 52952437 | High | Yes | Yes | No | No | No | No | No | -6.34 | 0 | 0 | 0 | 0 | 0 | 0.55 | 0 | 1 | 5.43 |
| 53438729 | High | No | No | Yes | No | Yes | No | Yes | -6.29 | 0 | 0 | 0 | 0 | 0 | 0.56 | 1 | 1 | 3 |
| 54580354 | Low | No | No | No | No | Yes | No | Yes | -4.83 | 2 | 4 | 0 | 1 | 1 | 0.17 | 0 | 0 | 6.01 |
| 54580355 | High | No | Yes | No | No | No | No | Yes | -5.83 | 1 | 3 | 0 | 0 | 1 | 0.55 | 0 | 1 | 6.64 |
| 54581351 | Low | No | Yes | No | No | No | No | No | -10.4 | 2 | 3 | 2 | 1 | 4 | 0.17 | 0 | 2 | 8.07 |
| 54581352 | High | No | Yes | No | No | No | No | No | -7.72 | 1 | 3 | 0 | 0 | 0 | 0.55 | 0 | 2 | 6.66 |
| 54583360 | Low | No | Yes | No | No | No | No | No | -10.4 | 2 | 3 | 2 | 1 | 4 | 0.17 | 0 | 2 | 8.07 |
| 54583361 | High | No | Yes | No | No | No | No | No | -7.3 | 1 | 3 | 0 | 0 | 0 | 0.55 | 0 | 3 | 6.86 |
| 54585273 | High | No | No | No | No | No | No | Yes | -8.4 | 0 | 1 | 0 | 0 | 0 | 0.55 | 0 | 2 | 6.18 |
| 54586223 | Low | No | Yes | No | No | No | No | No | -10.8 | 2 | 3 | 2 | 1 | 4 | 0.17 | 0 | 1 | 8.21 |
| 54587249 | Low | No | Yes | No | No | No | No | No | -7.15 | 1 | 3 | 0 | 0 | 0 | 0.55 | 0 | 3 | 7.04 |
| 54670067 | High | No | No | No | No | No | No | No | -8.54 | 0 | 2 | 0 | 0 | 1 | 0.56 | 0 | 0 | 3.47 |
| 54758525 | Low | No | No | No | No | No | No | No | -4.77 | 2 | 4 | 0 | 1 | 1 | 0.17 | 0 | 1 | 7.87 |
| 54758526 | High | No | Yes | No | No | No | No | No | -4.63 | 2 | 4 | 0 | 1 | 1 | 0.17 | 0 | 2 | 7.96 |
| 56841069 | High | No | No | No | No | Yes | No | No | -4.85 | 1 | 1 | 0 | 1 | 1 | 0.55 | 0 | 1 | 5.64 |
| 56958440 | High | No | No | No | No | No | No | No | -7.35 | 0 | 0 | 0 | 0 | 0 | 0.55 | 0 | 1 | 3.85 |
| 56958777 | High | No | No | No | No | No | No | No | -7.29 | 0 | 0 | 0 | 0 | 0 | 0.55 | 0 | 1 | 3.82 |
| 70689030 | High | No | Yes | No | No | No | No | No | -8.68 | 1 | 3 | 0 | 1 | 0 | 0.55 | 0 | 0 | 6.92 |
| 70697879 | High | No | Yes | No | No | No | No | No | -7.25 | 1 | 3 | 0 | 0 | 0 | 0.55 | 0 | 2 | 7 |
| 70697889 | High | No | Yes | No | No | Yes | No | No | -6.26 | 0 | 0 | 0 | 0 | 0 | 0.55 | 0 | 1 | 6.11 |
| 71338636 | High | Yes | No | No | No | No | No | No | -5.25 | 0 | 0 | 0 | 0 | 1 | 0.55 | 0 | 1 | 3.42 |
| 71413104 | High | No | No | Yes | No | No | No | Yes | -6.48 | 0 | 0 | 0 | 0 | 0 | 0.55 | 1 | 0 | 2.7 |
| 71584574 | High | No | Yes | Yes | No | Yes | Yes | Yes | -4.47 | 0 | 3 | 1 | 1 | 1 | 0.55 | 0 | 2 | 4.85 |
| 71584688 | Low | No | Yes | Yes | No | No | No | Yes | -3.57 | 1 | 4 | 1 | 1 | 2 | 0.55 | 0 | 2 | 5.22 |
| 71584689 | Low | No | Yes | No | No | No | No | Yes | -2.08 | 2 | 4 | 1 | 1 | 2 | 0.17 | 0 | 2 | 5.84 |
| 71584690 | Low | No | Yes | No | No | No | No | Yes | -2.61 | 2 | 4 | 1 | 1 | 2 | 0.17 | 0 | 2 | 5.97 |
| 71584691 | Low | No | Yes | No | No | No | No | Yes | -2.13 | 2 | 4 | 1 | 1 | 2 | 0.17 | 0 | 2 | 6.03 |
| 71717738 | High | Yes | No | Yes | Yes | Yes | No | Yes | -5.54 | 0 | 0 | 0 | 0 | 0 | 0.55 | 0 | 3 | 4.03 |
| 71720036 | High | Yes | No | No | No | No | No | No | -6.22 | 0 | 0 | 0 | 0 | 0 | 0.55 | 0 | 2 | 2.87 |
| 72738894 | High | No | No | No | Yes | No | Yes | No | -7.08 | 0 | 0 | 1 | 0 | 0 | 0.55 | 0 | 2 | 3.01 |
| 73076982 | High | No | Yes | No | No | No | No | No | -7 | 0 | 1 | 0 | 0 | 0 | 0.55 | 0 | 1 | 6.12 |
| 73187989 | High | Yes | No | Yes | Yes | Yes | Yes | Yes | -5.46 | 0 | 0 | 0 | 0 | 0 | 0.55 | 0 | 1 | 2.87 |
| 73356511 | High | No | Yes | No | No | No | No | No | -5.7 | 0 | 0 | 0 | 0 | 0 | 0.55 | 0 | 1 | 6.04 |
| 73797339 | Low | No | Yes | No | No | No | No | Yes | -5.75 | 2 | 4 | 0 | 1 | 2 | 0.17 | 0 | 2 | 7.49 |
| 73804953 | Low | No | Yes | No | No | No | No | No | -9.37 | 2 | 3 | 1 | 1 | 4 | 0.17 | 0 | 3 | 7.79 |
| 73813111 | High | No | Yes | No | No | Yes | No | No | -5.74 | 1 | 3 | 0 | 0 | 1 | 0.55 | 0 | 3 | 6.85 |
| 73824950 | Low | No | Yes | No | No | No | No | No | -10.2 | 2 | 3 | 2 | 1 | 4 | 0.17 | 0 | 3 | 8.15 |
| 73824951 | Low | No | Yes | No | No | No | No | No | -8.98 | 2 | 3 | 2 | 1 | 4 | 0.17 | 0 | 3 | 8.5 |
| 73824953 | Low | No | Yes | No | No | No | No | No | -9.62 | 2 | 3 | 1 | 1 | 4 | 0.17 | 0 | 2 | 8.24 |
| 74073445 | High | No | No | No | No | No | No | No | -7.13 | 0 | 0 | 0 | 0 | 1 | 0.55 | 0 | 0 | 4.44 |
| 74075981 | High | No | Yes | No | No | No | No | No | -5.44 | 1 | 3 | 0 | 0 | 1 | 0.55 | 0 | 1 | 7.16 |
| 74336648 | High | No | No | No | No | No | No | No | -7.84 | 0 | 0 | 0 | 0 | 0 | 0.55 | 0 | 1 | 3.96 |
| 74978378 | Low | No | Yes | No | No | No | No | No | -10.6 | 3 | 4 | 1 | 1 | 4 | 0.17 | 1 | 1 | 6.69 |
| 75050399 | High | Yes | No | Yes | Yes | Yes | No | Yes | -4.73 | 0 | 0 | 0 | 0 | 1 | 0.55 | 0 | 2 | 3.27 |
| 75050400 | High | Yes | No | Yes | Yes | Yes | Yes | Yes | -4.76 | 0 | 0 | 0 | 0 | 1 | 0.55 | 0 | 2 | 2.82 |
| 75111036 | Low | No | No | No | No | No | No | No | -10.6 | 3 | 2 | 1 | 1 | 3 | 0.17 | 0 | 0 | 5.66 |
| 75971805 | High | No | Yes | No | No | Yes | No | No | -5.34 | 1 | 4 | 0 | 1 | 1 | 0.55 | 0 | 1 | 6.52 |
| 75971891 | High | No | Yes | No | No | No | No | No | -5.45 | 1 | 3 | 0 | 0 | 1 | 0.55 | 0 | 2 | 6.69 |
| 75972015 | High | No | Yes | No | No | No | No | No | -7.72 | 1 | 3 | 0 | 0 | 0 | 0.55 | 0 | 2 | 6.66 |
| 76152128 | High | No | Yes | No | No | No | No | Yes | -5.83 | 1 | 3 | 0 | 0 | 1 | 0.55 | 0 | 1 | 6.64 |
| 76153908 | High | No | No | No | No | No | No | Yes | -8.4 | 0 | 1 | 0 | 0 | 0 | 0.55 | 0 | 2 | 6.18 |
| 76311433 | High | No | Yes | No | No | No | No | No | -5.31 | 1 | 3 | 0 | 0 | 1 | 0.55 | 0 | 1 | 6.35 |
| 76316558 | High | No | Yes | No | No | No | No | No | -5.84 | 0 | 1 | 0 | 0 | 0 | 0.55 | 0 | 1 | 6.02 |
| 76316561 | Low | No | Yes | No | No | No | No | No | -8.59 | 2 | 3 | 1 | 1 | 2 | 0.17 | 0 | 3 | 7.28 |
| 76317961 | High | No | Yes | No | No | No | No | No | -5.84 | 0 | 1 | 0 | 0 | 0 | 0.55 | 0 | 1 | 6.02 |
| 76327056 | Low | No | Yes | No | No | No | No | No | -9.36 | 2 | 3 | 1 | 1 | 4 | 0.17 | 0 | 3 | 7.87 |
| 76685092 | High | No | Yes | No | No | Yes | No | No | -6.03 | 0 | 0 | 0 | 0 | 0 | 0.55 | 0 | 2 | 6.21 |
| 77916018 | High | No | Yes | No | No | No | No | No | -7.25 | 1 | 3 | 0 | 0 | 0 | 0.55 | 0 | 2 | 7 |
| 78148404 | High | No | Yes | No | Yes | No | No | Yes | -7.11 | 1 | 3 | 0 | 0 | 0 | 0.55 | 0 | 2 | 6.71 |
| 78157935 | High | Yes | No | No | No | No | No | No | -6.22 | 0 | 0 | 0 | 0 | 0 | 0.55 | 0 | 2 | 2.87 |
| 78167002 | High | No | Yes | No | No | No | No | No | -5.53 | 0 | 3 | 0 | 0 | 1 | 0.55 | 0 | 1 | 6.36 |
| 85302768 | Low | No | No | No | No | No | No | No | -8.97 | 1 | 3 | 1 | 1 | 1 | 0.55 | 0 | 2 | 7.22 |
| 85596052 | Low | No | Yes | No | No | No | No | No | 6.03 | 2 | 4 | 1 | 1 | 3 | 0.17 | 0 | 0 | 6.5 |
| 85776164 | Low | No | No | Yes | No | Yes | No | No | -2.13 | 1 | 1 | 1 | 1 | 2 | 0.55 | 0 | 2 | 4.28 |
| 90470576 | High | No | Yes | No | No | No | No | No | -7.25 | 0 | 0 | 0 | 0 | 0 | 0.55 | 0 | 0 | 3.62 |
| 91884898 | Low | No | Yes | No | No | No | No | No | -9.71 | 2 | 3 | 1 | 1 | 2 | 0.17 | 0 | 1 | 6.74 |
| 91886694 | High | No | Yes | No | No | No | No | No | -7.12 | 1 | 3 | 0 | 0 | 0 | 0.55 | 0 | 3 | 6.86 |
| 100926540 | High | Yes | No | No | No | No | No | No | -6.85 | 0 | 0 | 0 | 0 | 0 | 0.55 | 0 | 1 | 4.56 |
| 100926541 | High | No | Yes | No | No | No | No | No | -8.97 | 0 | 0 | 0 | 0 | 0 | 0.55 | 0 | 1 | 5.95 |
| 100996181 | High | No | No | No | No | No | No | No | -3.58 | 1 | 3 | 0 | 1 | 1 | 0.55 | 0 | 1 | 5.18 |
| 101026859 | High | No | Yes | No | No | No | No | No | -6.65 | 1 | 3 | 0 | 0 | 0 | 0.55 | 0 | 1 | 6.81 |
| 101034965 | High | Yes | Yes | No | Yes | No | No | Yes | -5.54 | 0 | 0 | 0 | 0 | 0 | 0.55 | 0 | 0 | 3.44 |
| 101153492 | Low | No | Yes | No | No | No | No | Yes | -6.54 | 1 | 3 | 1 | 1 | 2 | 0.55 | 0 | 2 | 7.26 |
| 101153494 | High | No | No | No | No | No | No | No | -5.15 | 1 | 4 | 0 | 0 | 1 | 0.55 | 0 | 1 | 6.55 |
| 101280240 | High | No | No | No | No | No | No | No | -5.57 | 0 | 0 | 0 | 0 | 0 | 0.55 | 0 | 1 | 6.32 |
| 101289833 | High | No | Yes | No | No | No | No | No | -5.84 | 0 | 1 | 0 | 0 | 0 | 0.55 | 0 | 1 | 5.97 |
| 101355584 | Low | No | Yes | No | No | No | No | No | -8.06 | 1 | 3 | 1 | 1 | 0 | 0.55 | 0 | 3 | 6.76 |
| 101529198 | High | Yes | Yes | No | No | No | Yes | No | -5.33 | 0 | 0 | 0 | 0 | 0 | 0.55 | 0 | 0 | 3.7 |
| 101570727 | High | Yes | No | Yes | No | No | No | No | -6.07 | 0 | 0 | 0 | 0 | 0 | 0.55 | 0 | 2 | 3.1 |
| 101602319 | Low | No | No | No | No | No | No | No | -2.63 | 1 | 3 | 0 | 1 | 2 | 0.55 | 0 | 1 | 6.11 |
| 101602320 | High | No | Yes | No | No | No | No | No | -5.32 | 1 | 2 | 0 | 0 | 1 | 0.55 | 0 | 1 | 6.42 |
| 101602321 | High | No | Yes | No | No | No | No | No | -5.32 | 1 | 2 | 0 | 0 | 1 | 0.55 | 0 | 1 | 6.42 |
| 101634707 | High | No | Yes | No | No | No | No | No | -8.47 | 0 | 0 | 0 | 0 | 0 | 0.55 | 0 | 1 | 6.07 |
| 101676207 | Low | No | Yes | No | No | No | No | No | -9.54 | 2 | 2 | 1 | 1 | 2 | 0.17 | 0 | 1 | 6.41 |
| 101676208 | Low | No | Yes | No | No | No | No | No | -9.27 | 2 | 2 | 1 | 1 | 2 | 0.17 | 0 | 1 | 6.46 |
| 101676711 | Low | No | Yes | No | No | No | No | No | -9.54 | 2 | 2 | 1 | 1 | 2 | 0.17 | 0 | 1 | 6.41 |
| 101915817 | Low | No | Yes | No | No | No | No | No | -9.92 | 2 | 3 | 1 | 1 | 2 | 0.17 | 0 | 1 | 6.49 |
| 101916313 | High | No | Yes | No | No | No | No | No | -8.29 | 0 | 0 | 0 | 1 | 0 | 0.55 | 0 | 1 | 6.34 |
| 101919043 | Low | No | Yes | No | No | No | No | No | -9.11 | 2 | 3 | 1 | 1 | 4 | 0.17 | 0 | 3 | 7.86 |
| 101936072 | High | Yes | No | No | No | No | Yes | No | -7.34 | 0 | 0 | 0 | 0 | 0 | 0.55 | 0 | 1 | 6.05 |
| 101999884 | Low | No | Yes | No | No | No | No | No | -10.1 | 2 | 4 | 1 | 1 | 4 | 0.17 | 0 | 3 | 7.9 |
| 102034873 | High | Yes | No | No | Yes | Yes | Yes | No | -5.6 | 0 | 0 | 0 | 0 | 0 | 0.55 | 0 | 0 | 3.63 |
| 102063005 | Low | No | Yes | No | No | No | No | No | -8.97 | 2 | 3 | 1 | 1 | 4 | 0.17 | 0 | 3 | 7.96 |
| 102149247 | High | No | Yes | No | No | No | No | No | -8.19 | 1 | 3 | 0 | 0 | 1 | 0.55 | 0 | 3 | 6.99 |
| 102285347 | High | No | Yes | No | No | No | Yes | Yes | -7.95 | 0 | 0 | 0 | 0 | 0 | 0.55 | 0 | 1 | 5.99 |
| 102316534 | High | No | Yes | No | No | Yes | No | Yes | -5.73 | 0 | 3 | 0 | 0 | 1 | 0.56 | 0 | 1 | 6.32 |
| 102316535 | High | No | Yes | No | No | No | No | Yes | -5.45 | 0 | 3 | 0 | 0 | 1 | 0.55 | 0 | 0 | 6.39 |
| 118855989 | Low | No | Yes | No | No | No | No | No | -9.76 | 2 | 2 | 1 | 1 | 2 | 0.17 | 0 | 2 | 6.48 |
| 123981968 | High | Yes | No | Yes | No | No | No | No | -6.07 | 0 | 0 | 0 | 0 | 0 | 0.55 | 0 | 2 | 3.1 |
| 124305339 | High | Yes | No | No | Yes | No | No | No | -5 | 0 | 0 | 0 | 0 | 1 | 0.55 | 0 | 0 | 3.58 |
| 124629574 | High | No | Yes | No | No | No | No | No | -7.61 | 0 | 0 | 0 | 0 | 0 | 0.55 | 0 | 2 | 6.07 |
| 129010007 | Low | No | Yes | No | No | No | No | No | -10.1 | 3 | 4 | 1 | 1 | 4 | 0.17 | 0 | 0 | 6.34 |
| 129712290 | High | Yes | No | No | No | Yes | No | No | -4.5 | 1 | 0 | 0 | 0 | 0 | 0.55 | 0 | 2 | 4.32 |
| 131676058 | Low | No | Yes | No | No | No | No | No | -7.15 | 1 | 3 | 0 | 0 | 0 | 0.55 | 0 | 3 | 7.04 |
| 131698851 | Low | No | Yes | No | No | No | No | No | -9.92 | 2 | 3 | 1 | 1 | 4 | 0.17 | 0 | 3 | 8.11 |
| 131705161 | High | Yes | No | No | Yes | No | No | No | -5 | 0 | 0 | 0 | 0 | 1 | 0.55 | 0 | 0 | 3.58 |
| 131875206 | High | No | No | No | No | No | No | No | -5.6 | 0 | 0 | 0 | 0 | 0 | 0.55 | 0 | 0 | 5.82 |
| 132990894 | Low | No | Yes | No | No | No | No | No | -16 | 3 | 4 | 2 | 1 | 5 | 0.17 | 0 | 0 | 7.32 |
| 135369651 | High | Yes | No | Yes | No | No | No | No | -2.77 | 1 | 0 | 1 | 0 | 1 | 0.85 | 0 | 0 | 2.31 |
| 136360357 | High | Yes | No | No | No | No | No | No | -6.27 | 0 | 3 | 0 | 0 | 1 | 0.55 | 0 | 1 | 2.24 |
| 138115244 | High | No | Yes | No | No | No | No | No | -7.61 | 0 | 0 | 0 | 0 | 0 | 0.55 | 0 | 2 | 6.07 |
| 139057051 | High | Yes | No | Yes | No | No | No | No | -2.19 | 1 | 0 | 1 | 0 | 2 | 0.85 | 0 | 0 | 2.54 |
| 139057501 | High | No | Yes | No | No | No | No | No | -5.7 | 0 | 0 | 0 | 0 | 0 | 0.55 | 0 | 1 | 6.04 |
| 152743364 | Low | No | Yes | No | No | No | No | No | 0.04 | 2 | 4 | 0 | 1 | 2 | 0.17 | 0 | 1 | 6.19 |
| 154496877 | Low | No | No | No | Yes | Yes | No | No | -4.69 | 1 | 0 | 0 | 0 | 1 | 0.55 | 0 | 1 | 4.45 |
| 154497094 | High | No | Yes | No | No | No | No | No | -7.61 | 0 | 0 | 0 | 0 | 0 | 0.55 | 0 | 2 | 6.07 |
| 154497120 | High | No | Yes | No | No | No | No | No | -5.31 | 1 | 3 | 0 | 0 | 1 | 0.55 | 0 | 1 | 6.35 |
| 154497153 | High | Yes | No | No | Yes | Yes | No | No | -4.88 | 0 | 0 | 0 | 0 | 1 | 0.55 | 0 | 0 | 3.58 |
| 154497731 | Low | No | No | No | Yes | Yes | No | No | -4.16 | 1 | 0 | 0 | 0 | 1 | 0.55 | 0 | 1 | 5.53 |
| 162819794 | Low | No | Yes | No | No | No | No | Yes | -2.13 | 2 | 4 | 1 | 1 | 2 | 0.17 | 0 | 2 | 6.03 |
| 162842140 | High | Yes | Yes | No | No | No | No | Yes | -6.51 | 0 | 0 | 0 | 0 | 0 | 0.55 | 0 | 0 | 3.75 |
| 162845059 | High | No | No | Yes | Yes | Yes | No | No | -3.29 | 1 | 1 | 1 | 1 | 1 | 0.85 | 0 | 1 | 4 |
| 162845288 | High | Yes | No | Yes | Yes | Yes | No | No | -6.2 | 0 | 0 | 0 | 0 | 0 | 0.55 | 0 | 2 | 3.17 |
| 162847066 | High | Yes | No | Yes | Yes | Yes | No | Yes | -5.54 | 0 | 0 | 0 | 0 | 0 | 0.55 | 0 | 3 | 4.03 |
| 162847297 | Low | No | No | No | No | No | No | No | -10.6 | 3 | 3 | 1 | 1 | 3 | 0.11 | 0 | 0 | 6.6 |
| 162847298 | Low | No | No | No | No | No | No | No | -10.6 | 3 | 3 | 1 | 1 | 3 | 0.11 | 0 | 0 | 6.6 |
| 162853040 | High | No | Yes | No | No | No | No | No | -7.3 | 1 | 3 | 0 | 0 | 0 | 0.55 | 0 | 3 | 6.86 |
| 162853682 | Low | No | Yes | No | No | No | No | No | -8.27 | 2 | 3 | 1 | 1 | 2 | 0.17 | 0 | 3 | 7.32 |
| 162853683 | Low | No | Yes | No | No | No | No | No | -8.27 | 2 | 3 | 1 | 1 | 2 | 0.17 | 0 | 3 | 7.32 |
| 162857811 | High | No | Yes | Yes | No | Yes | Yes | Yes | -6.37 | 0 | 0 | 0 | 0 | 0 | 0.55 | 0 | 2 | 3.76 |
| 162859638 | High | No | Yes | No | No | No | No | No | -6.49 | 0 | 3 | 0 | 0 | 0 | 0.55 | 0 | 1 | 6.29 |
| 162867112 | High | Yes | No | Yes | Yes | Yes | No | No | -6.28 | 0 | 0 | 0 | 0 | 0 | 0.55 | 0 | 2 | 3.74 |
| 162867118 | High | No | No | No | No | No | No | No | -7.06 | 0 | 0 | 0 | 0 | 0 | 0.55 | 0 | 2 | 3.19 |
| 162867119 | High | Yes | No | Yes | Yes | Yes | Yes | No | -5.55 | 0 | 0 | 0 | 0 | 0 | 0.55 | 0 | 2 | 3.18 |
| 162867134 | Low | No | Yes | No | No | No | No | No | -6.05 | 0 | 0 | 0 | 0 | 0 | 0.56 | 0 | 2 | 5.1 |
| 162867348 | High | No | Yes | No | No | No | No | No | -8.5 | 0 | 0 | 0 | 1 | 0 | 0.55 | 0 | 1 | 5.12 |
| 162874247 | High | No | Yes | No | No | No | No | No | -5.96 | 1 | 3 | 0 | 0 | 1 | 0.55 | 0 | 2 | 6.91 |
| 162874358 | High | No | Yes | No | No | No | No | No | -5.88 | 1 | 3 | 0 | 0 | 1 | 0.55 | 0 | 3 | 6.98 |
| 162876410 | Low | No | Yes | No | No | No | No | No | -9.36 | 2 | 3 | 1 | 1 | 4 | 0.17 | 0 | 3 | 7.87 |
| 162876411 | Low | No | Yes | No | No | No | No | No | -9.36 | 2 | 3 | 1 | 1 | 4 | 0.17 | 0 | 3 | 7.87 |
| 162878377 | High | Yes | No | No | No | No | No | No | -5.83 | 0 | 1 | 0 | 0 | 2 | 0.55 | 0 | 0 | 3.98 |
| 162878378 | High | Yes | No | No | No | No | No | No | -5.83 | 0 | 1 | 0 | 0 | 2 | 0.55 | 0 | 0 | 3.98 |
| 162880289 | Low | No | No | No | No | No | No | No | -4.1 | 1 | 4 | 0 | 1 | 1 | 0.55 | 0 | 1 | 5.99 |
| 162884805 | High | No | Yes | No | No | Yes | No | No | -5.73 | 0 | 3 | 0 | 0 | 0 | 0.55 | 0 | 2 | 6.41 |
| 162885142 | High | Yes | No | No | No | Yes | Yes | No | -3.09 | 0 | 0 | 1 | 0 | 1 | 0.85 | 0 | 0 | 3.78 |
| 162885807 | Low | No | Yes | No | Yes | No | No | No | -7.6 | 2 | 4 | 2 | 2 | 4 | 0.17 | 0 | 1 | 7.97 |
| 162888038 | High | No | Yes | No | No | No | No | No | -5.31 | 1 | 3 | 0 | 0 | 1 | 0.55 | 0 | 1 | 6.35 |
| 162890111 | Low | No | Yes | No | No | No | No | No | -9.37 | 2 | 3 | 1 | 1 | 4 | 0.17 | 0 | 3 | 7.79 |
| 162893251 | High | No | Yes | No | No | No | Yes | Yes | -7.95 | 0 | 0 | 0 | 0 | 0 | 0.55 | 0 | 1 | 5.99 |
| 162895380 | Low | No | No | No | No | No | No | No | -2.63 | 1 | 3 | 0 | 1 | 2 | 0.55 | 0 | 1 | 6.11 |
| 162895538 | High | No | No | No | No | No | No | No | -7.9 | 0 | 1 | 0 | 0 | 0 | 0.55 | 0 | 2 | 4.66 |
| 162897369 | High | No | Yes | No | No | No | No | No | -6.37 | 0 | 0 | 0 | 0 | 0 | 0.55 | 0 | 1 | 6.52 |
| 162898546 | High | No | Yes | No | No | No | No | No | -6.37 | 0 | 0 | 0 | 0 | 0 | 0.55 | 0 | 1 | 6.52 |
| 162899427 | Low | No | Yes | No | No | No | No | No | -10.8 | 3 | 4 | 1 | 1 | 4 | 0.17 | 0 | 0 | 6.51 |
| 162900804 | High | No | Yes | No | No | No | No | No | -6.77 | 0 | 3 | 0 | 0 | 0 | 0.55 | 0 | 1 | 6.56 |
| 162902728 | High | No | No | No | No | No | No | Yes | -8.32 | 0 | 1 | 0 | 0 | 0 | 0.55 | 0 | 2 | 6.28 |
| 162903500 | High | Yes | No | No | No | No | Yes | No | -3.6 | 0 | 0 | 0 | 0 | 1 | 0.55 | 0 | 0 | 4.79 |
| 162905858 | High | No | Yes | No | No | No | No | No | -6.82 | 0 | 2 | 0 | 0 | 0 | 0.55 | 0 | 2 | 6.37 |
| 162910735 | Low | No | Yes | No | No | No | No | No | -9.75 | 2 | 4 | 1 | 1 | 2 | 0.17 | 0 | 1 | 6.66 |
| 162910955 | High | No | Yes | No | No | Yes | No | No | -5.74 | 1 | 3 | 0 | 0 | 1 | 0.55 | 0 | 3 | 6.85 |
| 162912527 | High | Yes | No | No | No | No | No | No | -6.85 | 0 | 0 | 0 | 0 | 0 | 0.55 | 0 | 1 | 4.56 |
| 162913077 | Low | No | Yes | No | No | No | No | No | -12.9 | 3 | 2 | 1 | 1 | 4 | 0.11 | 0 | 0 | 6.19 |
| 162917693 | High | No | Yes | No | No | Yes | No | No | -6.54 | 1 | 3 | 0 | 0 | 0 | 0.55 | 0 | 2 | 6.54 |
| 162918748 | High | No | Yes | No | No | No | No | No | -6.14 | 0 | 0 | 0 | 0 | 0 | 0.55 | 0 | 1 | 6.34 |
| 162920427 | Low | No | Yes | No | No | No | No | No | -11.2 | 2 | 4 | 1 | 1 | 4 | 0.17 | 0 | 2 | 7.79 |
| 162920428 | Low | No | Yes | No | No | No | No | No | -11.2 | 2 | 4 | 1 | 1 | 4 | 0.17 | 0 | 2 | 7.79 |
| 162920595 | High | No | Yes | No | No | No | No | No | -8.77 | 0 | 0 | 0 | 0 | 0 | 0.55 | 0 | 0 | 5.38 |
| 162921038 | High | No | Yes | No | No | No | No | No | -7.14 | 0 | 2 | 0 | 0 | 0 | 0.55 | 0 | 2 | 6.35 |
| 162921039 | High | No | Yes | No | No | No | No | No | -7.14 | 0 | 2 | 0 | 0 | 0 | 0.55 | 0 | 2 | 6.35 |
| 162921836 | Low | No | No | No | No | No | No | No | -10.7 | 3 | 4 | 1 | 1 | 3 | 0.11 | 0 | 0 | 6.76 |
| 162921838 | Low | No | No | No | No | No | No | No | -10.7 | 3 | 4 | 1 | 1 | 3 | 0.11 | 0 | 0 | 6.76 |
| 162922533 | High | No | Yes | No | No | No | No | No | -8.46 | 0 | 1 | 0 | 0 | 0 | 0.55 | 0 | 2 | 6.37 |
| 162922614 | Low | No | Yes | No | No | No | No | No | -10.6 | 2 | 1 | 1 | 1 | 4 | 0.17 | 0 | 0 | 7.13 |
| 162925476 | Low | No | Yes | No | No | No | No | No | -12.9 | 3 | 2 | 1 | 1 | 4 | 0.11 | 0 | 0 | 6.19 |
| 162925999 | High | No | Yes | No | No | No | No | No | -6.55 | 0 | 0 | 0 | 0 | 0 | 0.55 | 0 | 0 | 5.62 |
| 162934403 | High | No | No | No | No | No | No | No | -4.52 | 2 | 4 | 0 | 1 | 1 | 0.17 | 0 | 1 | 6.41 |
| 162935328 | Low | No | No | No | No | No | Yes | No | -9.32 | 2 | 3 | 2 | 1 | 3 | 0.17 | 0 | 1 | 7.24 |
| 162935329 | Low | No | No | No | No | No | Yes | No | -9.32 | 2 | 3 | 2 | 1 | 3 | 0.17 | 0 | 1 | 7.24 |
| 162938666 | High | Yes | No | No | Yes | Yes | No | Yes | -5.32 | 0 | 0 | 0 | 0 | 0 | 0.55 | 0 | 2 | 3.77 |
| 162940876 | High | No | No | No | No | No | No | No | -7.74 | 0 | 0 | 0 | 0 | 0 | 0.55 | 0 | 3 | 4.88 |
| 162944734 | Low | No | Yes | No | No | No | No | No | -10.7 | 3 | 4 | 1 | 1 | 4 | 0.17 | 0 | 1 | 6.4 |
| 162946727 | Low | No | Yes | No | No | No | No | No | -9.79 | 2 | 3 | 1 | 1 | 2 | 0.17 | 0 | 2 | 6.49 |
| 162949507 | High | No | Yes | No | No | No | No | No | -8.51 | 1 | 3 | 0 | 0 | 1 | 0.55 | 0 | 3 | 6.95 |
| 162951523 | Low | No | Yes | No | No | No | No | No | -8.57 | 1 | 3 | 0 | 1 | 0 | 0.55 | 0 | 3 | 7.1 |
| 162952996 | High | No | Yes | No | No | Yes | No | No | -6.15 | 0 | 1 | 0 | 0 | 0 | 0.55 | 0 | 1 | 6.2 |
| 162953557 | High | No | Yes | No | No | No | No | No | -6.44 | 0 | 0 | 0 | 0 | 0 | 0.56 | 0 | 0 | 5.76 |
| 162955224 | High | No | Yes | No | No | No | No | No | -7.35 | 1 | 3 | 0 | 0 | 1 | 0.55 | 0 | 3 | 7.34 |
| 162956849 | High | No | Yes | No | No | No | No | No | -6.85 | 0 | 3 | 0 | 0 | 0 | 0.55 | 0 | 2 | 6.47 |
| 162957338 | High | No | No | No | No | No | No | No | -8.31 | 0 | 0 | 0 | 0 | 0 | 0.55 | 0 | 1 | 2.6 |
| 162963098 | High | No | Yes | No | No | No | Yes | No | -6.79 | 0 | 0 | 0 | 0 | 0 | 0.55 | 0 | 0 | 3.81 |
| 162963358 | High | No | Yes | No | No | Yes | No | No | -6.03 | 0 | 0 | 0 | 0 | 0 | 0.56 | 0 | 1 | 5.29 |
| 162963628 | High | No | Yes | No | No | No | No | No | -7.61 | 0 | 0 | 0 | 0 | 0 | 0.55 | 0 | 2 | 6.07 |
| 162964435 | High | No | Yes | No | No | No | No | No | -8.79 | 0 | 1 | 0 | 0 | 0 | 0.55 | 0 | 2 | 6.3 |
| 162965363 | Low | No | No | No | No | No | No | No | -2.2 | 1 | 3 | 0 | 1 | 2 | 0.55 | 0 | 1 | 6.3 |
| 162968547 | High | No | No | No | No | No | No | No | -7.74 | 0 | 0 | 0 | 0 | 0 | 0.55 | 0 | 3 | 4.85 |
| 162970859 | High | No | No | No | No | No | Yes | Yes | -7.8 | 0 | 1 | 0 | 0 | 0 | 0.55 | 0 | 2 | 6.21 |
| 162971118 | Low | No | No | No | No | No | No | No | -4.26 | 2 | 4 | 0 | 1 | 1 | 0.17 | 0 | 1 | 7.92 |
| 162971309 | Low | No | Yes | No | No | No | No | No | -11.4 | 3 | 4 | 1 | 1 | 4 | 0.17 | 0 | 0 | 6.84 |
| 162971728 | Low | No | Yes | No | Yes | Yes | Yes | No | -5.18 | 1 | 3 | 1 | 0 | 1 | 0.55 | 0 | 1 | 4.38 |
| 162973005 | High | No | No | No | No | Yes | No | No | -4.9 | 1 | 1 | 0 | 1 | 1 | 0.55 | 0 | 1 | 5.87 |
| 162973509 | High | No | Yes | No | No | No | No | No | -8.47 | 0 | 0 | 0 | 0 | 0 | 0.55 | 0 | 1 | 6.07 |
| 162973510 | High | No | Yes | No | No | No | No | No | -8.47 | 0 | 0 | 0 | 0 | 0 | 0.55 | 0 | 1 | 6.07 |
| 162974828 | Low | No | Yes | No | No | No | No | No | -9.41 | 2 | 3 | 1 | 1 | 4 | 0.17 | 0 | 4 | 7.81 |
| 162975776 | High | No | Yes | No | No | No | No | No | -6.57 | 1 | 3 | 0 | 0 | 0 | 0.55 | 0 | 2 | 6.86 |
| 162975883 | High | Yes | No | No | No | No | Yes | No | -7.34 | 0 | 0 | 0 | 0 | 0 | 0.55 | 0 | 1 | 6.05 |
| 162976009 | Low | No | Yes | No | No | No | No | Yes | -7.11 | 1 | 3 | 0 | 0 | 0 | 0.55 | 0 | 2 | 7.12 |
| 162981932 | High | No | Yes | No | No | No | No | No | -5.85 | 1 | 3 | 0 | 0 | 0 | 0.55 | 0 | 0 | 7.17 |
| 162983832 | Low | No | Yes | No | No | No | No | No | -15.9 | 3 | 4 | 1 | 1 | 5 | 0.11 | 0 | 0 | 7.26 |
| 162984585 | High | No | Yes | No | No | No | No | Yes | -6.38 | 1 | 3 | 0 | 0 | 0 | 0.55 | 0 | 1 | 6.97 |
| 162993729 | Low | No | Yes | No | No | No | No | No | -8.97 | 2 | 3 | 1 | 1 | 4 | 0.17 | 0 | 3 | 7.96 |
| 162995221 | High | No | Yes | No | No | No | No | No | -6.13 | 1 | 3 | 0 | 0 | 0 | 0.55 | 0 | 1 | 6.79 |
| 162995965 | High | No | Yes | No | No | Yes | No | No | -6.03 | 0 | 0 | 0 | 0 | 0 | 0.55 | 0 | 2 | 6.21 |
| 162999362 | High | No | Yes | No | No | No | No | No | -7.72 | 1 | 3 | 0 | 0 | 0 | 0.55 | 0 | 2 | 6.66 |
| 163003104 | Low | No | Yes | No | No | No | No | Yes | -2.61 | 2 | 4 | 1 | 1 | 2 | 0.17 | 0 | 2 | 5.97 |
| 163003554 | High | No | Yes | No | No | Yes | No | No | -5.62 | 0 | 1 | 0 | 0 | 0 | 0.55 | 0 | 1 | 6.21 |
| 163003555 | High | No | Yes | No | No | Yes | No | No | -5.62 | 0 | 1 | 0 | 0 | 0 | 0.55 | 0 | 1 | 6.21 |
| 163004540 | High | No | No | No | No | No | No | No | -5.57 | 0 | 3 | 0 | 0 | 1 | 0.55 | 0 | 1 | 6.67 |
| 163004763 | Low | No | No | No | No | No | No | No | -1.43 | 1 | 3 | 1 | 1 | 2 | 0.55 | 0 | 0 | 4.84 |
| 163005368 | High | No | No | No | No | No | No | Yes | -7.94 | 0 | 0 | 0 | 0 | 0 | 0.55 | 0 | 0 | 2.56 |
| 163010578 | High | No | Yes | No | No | Yes | No | No | -6.26 | 0 | 0 | 0 | 0 | 0 | 0.55 | 0 | 1 | 6.11 |
| 163011590 | High | No | No | No | No | Yes | No | Yes | -4.17 | 0 | 0 | 0 | 0 | 1 | 0.55 | 0 | 1 | 4.33 |
| 163012984 | High | Yes | Yes | No | No | No | Yes | No | -5.33 | 0 | 0 | 0 | 0 | 0 | 0.55 | 0 | 0 | 3.7 |
| 163014941 | Low | No | Yes | No | No | No | No | No | -8.85 | 3 | 3 | 2 | 1 | 3 | 0.17 | 0 | 0 | 5.53 |
| 163015292 | Low | No | Yes | No | No | No | No | No | -15.8 | 3 | 4 | 1 | 1 | 5 | 0.11 | 0 | 0 | 7.22 |
| 163016882 | Low | No | Yes | No | No | No | No | No | -8.93 | 0 | 1 | 1 | 1 | 1 | 0.55 | 0 | 1 | 6.37 |
| 163017755 | High | No | No | Yes | No | Yes | Yes | Yes | -5.62 | 0 | 0 | 0 | 0 | 0 | 0.55 | 1 | 3 | 3.65 |
| 163018012 | High | No | Yes | No | No | No | Yes | No | -8.2 | 0 | 0 | 0 | 0 | 0 | 0.56 | 0 | 1 | 6.26 |
| 163018356 | High | No | Yes | No | No | No | No | No | -6.86 | 0 | 0 | 0 | 0 | 0 | 0.55 | 0 | 2 | 5.23 |
| 163021866 | Low | No | Yes | No | No | No | No | No | -9.27 | 2 | 2 | 1 | 1 | 2 | 0.17 | 0 | 1 | 6.46 |
| 163025008 | Low | No | Yes | No | No | No | No | No | -12.3 | 2 | 1 | 1 | 1 | 4 | 0.17 | 0 | 0 | 6.17 |
| 163026334 | Low | No | Yes | No | No | No | No | No | -9.71 | 2 | 3 | 1 | 1 | 2 | 0.17 | 0 | 1 | 6.74 |
| 163026908 | Low | No | No | No | No | No | No | No | -8.36 | 1 | 3 | 1 | 1 | 1 | 0.55 | 0 | 2 | 7 |
| 163028748 | Low | No | Yes | No | Yes | No | No | No | -8 | 2 | 3 | 2 | 1 | 4 | 0.17 | 0 | 1 | 7.65 |
| 163029829 | High | No | Yes | No | No | Yes | No | No | -6.24 | 1 | 3 | 0 | 0 | 0 | 0.55 | 0 | 3 | 6.89 |
| 163030723 | High | No | Yes | No | No | No | No | No | -5.7 | 0 | 0 | 0 | 0 | 0 | 0.55 | 0 | 1 | 6 |
| 163030830 | Low | No | Yes | No | No | No | No | Yes | -7.66 | 1 | 3 | 0 | 1 | 1 | 0.55 | 0 | 4 | 7.11 |
| 163033851 | Low | No | Yes | No | No | No | No | No | -9.83 | 2 | 3 | 1 | 1 | 3 | 0.17 | 0 | 3 | 7.11 |
| 163036215 | High | No | Yes | No | No | No | No | No | -5.67 | 0 | 0 | 0 | 0 | 0 | 0.55 | 0 | 1 | 5.87 |
| 163040884 | Low | No | Yes | No | No | No | Yes | No | -11 | 2 | 3 | 1 | 1 | 3 | 0.17 | 0 | 3 | 8.74 |
| 163042119 | High | No | Yes | No | No | No | No | No | -7.69 | 1 | 3 | 0 | 1 | 0 | 0.55 | 0 | 1 | 6.53 |
| 163042754 | Low | No | No | No | No | No | No | No | -4.32 | 1 | 4 | 0 | 0 | 1 | 0.55 | 0 | 1 | 8.02 |
| 163045194 | High | No | No | Yes | No | Yes | Yes | Yes | -5.17 | 0 | 0 | 0 | 0 | 0 | 0.55 | 0 | 1 | 3.56 |
| 163046290 | High | No | Yes | No | No | No | No | No | -6.94 | 1 | 3 | 0 | 0 | 0 | 0.55 | 0 | 3 | 6.98 |
| 163046350 | High | Yes | No | No | No | Yes | Yes | No | -3.63 | 1 | 0 | 0 | 0 | 1 | 0.55 | 0 | 1 | 3.48 |
| 163047849 | Low | No | No | No | No | No | No | No | -8.97 | 1 | 3 | 1 | 1 | 1 | 0.55 | 0 | 2 | 7.22 |
| 163049962 | High | No | Yes | No | No | No | No | Yes | -6.76 | 0 | 0 | 0 | 0 | 0 | 0.55 | 0 | 1 | 6.2 |
| 163057991 | High | No | Yes | No | No | Yes | No | Yes | -5.73 | 0 | 3 | 0 | 0 | 1 | 0.56 | 0 | 1 | 6.32 |
| 163059685 | Low | No | Yes | No | No | No | No | No | -12.3 | 2 | 1 | 1 | 1 | 4 | 0.17 | 0 | 0 | 6.17 |
| 163063327 | High | No | Yes | No | No | Yes | No | No | -6.35 | 0 | 0 | 0 | 0 | 0 | 0.55 | 0 | 2 | 6.19 |
| 163063328 | High | No | Yes | No | No | Yes | No | No | -6.35 | 0 | 0 | 0 | 0 | 0 | 0.55 | 0 | 2 | 6.19 |
| 163067369 | Low | No | No | No | No | No | Yes | No | -9.06 | 2 | 3 | 2 | 1 | 3 | 0.17 | 0 | 1 | 7.27 |
| 163069224 | Low | No | Yes | No | No | No | No | No | -9.62 | 3 | 3 | 1 | 1 | 3 | 0.11 | 0 | 1 | 6.7 |
| 163070745 | High | No | Yes | No | No | No | No | Yes | -7.04 | 0 | 3 | 0 | 0 | 0 | 0.55 | 0 | 0 | 6.34 |
| 163072561 | High | Yes | No | No | Yes | Yes | Yes | No | -5.74 | 0 | 0 | 0 | 0 | 0 | 0.55 | 0 | 0 | 3.35 |
| 163075353 | High | Yes | No | No | No | Yes | Yes | No | -3.5 | 1 | 0 | 0 | 0 | 2 | 0.55 | 0 | 1 | 5.12 |
| 163075826 | High | No | Yes | No | No | No | No | No | -6.54 | 1 | 3 | 0 | 0 | 0 | 0.55 | 0 | 2 | 6.74 |
| 163075861 | High | No | No | No | No | No | No | Yes | -8.22 | 0 | 0 | 0 | 0 | 0 | 0.55 | 0 | 2 | 6.11 |
| 163078747 | Low | No | No | No | No | No | No | No | -4.77 | 2 | 4 | 0 | 1 | 1 | 0.17 | 0 | 1 | 7.87 |
| 163080755 | High | No | Yes | No | No | No | No | No | -6.52 | 0 | 3 | 0 | 0 | 0 | 0.55 | 0 | 2 | 6.56 |
| 163082705 | High | No | Yes | No | No | No | No | No | -6.4 | 0 | 3 | 0 | 0 | 0 | 0.55 | 0 | 1 | 6.34 |
| 163082706 | High | No | Yes | No | No | No | No | No | -6.4 | 0 | 3 | 0 | 0 | 0 | 0.55 | 0 | 1 | 6.34 |
| 163084105 | High | No | Yes | No | No | No | No | No | -8.04 | 1 | 3 | 0 | 1 | 0 | 0.55 | 0 | 2 | 6.7 |
| 163085934 | High | No | No | No | No | No | No | No | -5.15 | 1 | 4 | 0 | 0 | 1 | 0.55 | 0 | 1 | 6.55 |
| 163086875 | High | No | No | No | No | No | No | Yes | -8 | 1 | 3 | 0 | 0 | 0 | 0.55 | 0 | 2 | 6.4 |
| 163105183 | High | No | Yes | No | No | No | No | No | -8.56 | 0 | 0 | 0 | 0 | 0 | 0.55 | 0 | 0 | 5.95 |
| 163105607 | High | No | No | No | No | Yes | No | No | -3.71 | 1 | 0 | 0 | 0 | 1 | 0.85 | 0 | 1 | 3.57 |
| 163115075 | Low | No | No | No | No | No | No | No | -9.1 | 2 | 3 | 1 | 1 | 2 | 0.17 | 0 | 2 | 6.83 |
| 163185371 | High | No | Yes | No | No | No | No | No | -7.3 | 1 | 3 | 0 | 0 | 0 | 0.55 | 0 | 3 | 6.86 |
| 163185419 | High | No | Yes | No | No | No | No | No | -5.74 | 0 | 3 | 0 | 0 | 1 | 0.55 | 0 | 1 | 6.96 |
| 163185622 | High | Yes | Yes | Yes | Yes | Yes | Yes | Yes | -5.35 | 0 | 0 | 0 | 0 | 0 | 0.55 | 0 | 2 | 3.51 |
| 163185892 | Low | No | No | No | No | No | No | No | -10.7 | 3 | 4 | 1 | 1 | 3 | 0.11 | 0 | 0 | 6.76 |
| 163186393 | High | Yes | No | No | No | No | Yes | No | -4.87 | 0 | 0 | 0 | 0 | 0 | 0.55 | 0 | 1 | 4.97 |
| 163186826 | Low | No | No | No | No | No | No | No | -10.6 | 3 | 3 | 1 | 1 | 3 | 0.11 | 0 | 0 | 6.6 |
| 163186897 | High | Yes | No | Yes | Yes | Yes | Yes | Yes | -5.91 | 0 | 0 | 0 | 0 | 0 | 0.55 | 0 | 1 | 3.19 |
| 163187092 | High | No | No | No | No | Yes | No | Yes | -5.2 | 0 | 0 | 0 | 0 | 1 | 0.55 | 0 | 1 | 4.6 |
| 163187632 | High | Yes | Yes | Yes | Yes | Yes | Yes | Yes | -5.65 | 0 | 0 | 0 | 0 | 0 | 0.55 | 0 | 2 | 3.34 |
| 163188089 | High | No | Yes | No | No | No | No | No | -6.74 | 0 | 1 | 0 | 0 | 0 | 0.55 | 0 | 1 | 6.4 |
| 163188272 | High | No | Yes | Yes | No | Yes | Yes | Yes | -6.37 | 0 | 0 | 0 | 0 | 0 | 0.55 | 0 | 2 | 3.76 |
| 163188791 | Low | No | Yes | No | No | No | No | No | -7.15 | 1 | 3 | 0 | 0 | 0 | 0.55 | 0 | 3 | 7.04 |
| 163189088 | High | Yes | Yes | Yes | Yes | Yes | Yes | Yes | -5.5 | 0 | 0 | 1 | 0 | 0 | 0.55 | 0 | 2 | 3.65 |
| 163190772 | Low | No | Yes | No | No | No | No | No | -13.4 | 3 | 2 | 1 | 1 | 4 | 0.11 | 0 | 0 | 6.19 |
| 163192272 | High | Yes | No | No | No | No | No | No | -5.74 | 0 | 0 | 0 | 0 | 0 | 0.55 | 0 | 0 | 3.77 |
| 163193473 | Low | No | Yes | No | No | No | No | No | -9.62 | 3 | 3 | 1 | 1 | 3 | 0.11 | 0 | 1 | 6.7 |
| 163193561 | High | No | No | No | No | No | No | Yes | -8.32 | 0 | 1 | 0 | 0 | 0 | 0.55 | 0 | 2 | 6.28 |
| 163193927 | Low | No | Yes | No | No | No | No | Yes | -8.63 | 2 | 3 | 1 | 1 | 3 | 0.17 | 0 | 2 | 7.31 |
| 163194738 | Low | No | Yes | No | No | No | No | Yes | -6.36 | 1 | 3 | 1 | 1 | 2 | 0.55 | 0 | 1 | 7.02 |
| 163195533 | Low | No | Yes | No | No | No | No | No | -6.71 | 1 | 3 | 0 | 0 | 1 | 0.55 | 0 | 3 | 7.17 |

*P stands for property, where P1 - GI Absorption, P2 – BBB Permeant, P3 - Pgp substrate, P4 - CYP1A2 inhibitor, P5 - CYP2C19 inhibitor, P6 - CYP2C9 inhibitor, P7 - CYP2D6 inhibitor, P8 - CYP3A4 inhibitor, P9 – Skin permeation in log Kp (cm/s), P10 – No. of violations in Lipinski rule, P11 – No. of violations in Ghose rule, P12 – No. of violations in Veber rule, P13 – No. of violations in Egan rule, P14 – No. of violations in Muegge rule, P15 - Bioavailability Score, P16 – No. of alerts for PAINS, P17 – No. of alerts for Brenk, P18 - Synthetic Accessibility score
