# Supplementary material for: Antidiabetic potential of a novel hydroxyphenyl-bi-benzopyran-hexol compound from Cassia fistula as an α-amylase inhibitor: Integrated in silico screening and in vitro validation using a nonlinear regression model
Source: J Ayurveda Integr Med. 2026 Jul 24;17(4):101366. doi: 10.1016/j.jaim.2026.101366 (PMC13420717; doi:10.1016/j.jaim.2026.101366)
Supplement: Multimedia component 3 [file mmc3.docx]

**Table S3** Probability of toxicity and carcinogenicity of natural compounds

| **CID** | **DILI** | **Ames** | **AOT** | **Car** | **CID** | **DILI** | **Ames** | **AOT** | **Car** |
| --- | --- | --- | --- | --- | --- | --- | --- | --- | --- |
| 305 | 0.00 | 0.15 | 0.55 | 0.43 | 10207 | 0.91 | 0.92 | 0.36 | 0.83 |
| 335 | 0.16 | 0.37 | 0.45 | 0.73 | 10208 | 0.87 | 0.91 | 0.48 | 0.77 |
| 342 | 0.21 | 0.35 | 0.49 | 0.82 | 10212 | 0.77 | 0.53 | 0.60 | 0.79 |
| 370 | 0.70 | 0.48 | 0.25 | 0.22 | 10393 | 0.04 | 0.32 | 0.09 | 0.46 |
| 460 | 0.25 | 0.50 | 0.33 | 0.70 | 10416 | 0.11 | 0.09 | 0.19 | 0.33 |
| 753 | 0.01 | 0.33 | 0.03 | 0.18 | 10469 | 0.14 | 0.01 | 0.09 | 0.17 |
| 860 | 0.01 | 0.38 | 0.09 | 0.23 | 10582 | 0.17 | 0.11 | 0.11 | 0.63 |
| 965 | 0.01 | 0.08 | 0.07 | 0.17 | 10639 | 0.96 | 0.93 | 0.53 | 0.84 |
| 1183 | 0.28 | 0.46 | 0.21 | 0.58 | 10964 | 0.53 | 0.64 | 0.25 | 0.38 |
| 1203 | 0.18 | 0.53 | 0.50 | 0.38 | 11005 | 0.20 | 0.06 | 0.13 | 0.29 |
| 2263 | 0.64 | 0.97 | 0.85 | 0.25 | 11197 | 0.15 | 0.01 | 0.09 | 0.19 |
| 2353 | 0.18 | 0.42 | 0.87 | 0.81 | 11230 | 0.16 | 0.32 | 0.25 | 0.60 |
| 2355 | 0.89 | 0.60 | 0.67 | 0.84 | 11463 | 0.31 | 0.33 | 0.33 | 0.73 |
| 2879 | 0.20 | 0.34 | 0.49 | 0.85 | 11622 | 0.17 | 0.09 | 0.15 | 0.42 |
| 3220 | 0.83 | 0.91 | 0.50 | 0.77 | 11636 | 0.11 | 0.01 | 0.16 | 0.16 |
| 3248 | 0.07 | 0.62 | 0.25 | 0.37 | 11850 | 0.00 | 0.39 | 0.02 | 0.06 |
| 3893 | 0.21 | 0.08 | 0.14 | 0.32 | 12177 | 0.28 | 0.48 | 0.27 | 0.54 |
| 3931 | 0.00 | 0.13 | 0.06 | 0.10 | 12366 | 0.15 | 0.05 | 0.08 | 0.39 |
| 4114 | 0.86 | 0.57 | 0.67 | 0.84 | 12377 | 0.99 | 0.43 | 0.57 | 0.15 |
| 5793 | 0.23 | 0.64 | 0.28 | 0.29 | 12405 | 0.12 | 0.03 | 0.19 | 0.21 |
| 5984 | 0.01 | 0.27 | 0.02 | 0.26 | 12409 | 0.10 | 0.01 | 0.15 | 0.15 |
| 5988 | 0.24 | 0.60 | 0.20 | 0.22 | 12410 | 0.10 | 0.01 | 0.14 | 0.13 |
| 5997 | 0.07 | 0.10 | 0.13 | 0.40 | 12575 | 0.58 | 0.40 | 0.28 | 0.43 |
| 6054 | 0.05 | 0.27 | 0.07 | 0.39 | 12921 | 0.10 | 0.08 | 0.18 | 0.32 |
| 6214 | 0.00 | 0.91 | 0.10 | 0.72 | 13187 | 0.19 | 0.14 | 0.18 | 0.47 |
| 6466 | 0.56 | 0.55 | 0.41 | 0.38 | 14896 | 0.32 | 0.07 | 0.13 | 0.60 |
| 6549 | 0.07 | 0.37 | 0.19 | 0.61 | 16592 | 1.00 | 0.46 | 0.52 | 0.14 |
| 6654 | 0.33 | 0.02 | 0.05 | 0.63 | 18818 | 0.18 | 0.48 | 0.44 | 0.63 |
| 6656 | 0.57 | 0.35 | 0.39 | 0.52 | 19009 | 0.09 | 0.29 | 0.87 | 0.66 |
| 6760 | 0.75 | 0.63 | 0.55 | 0.82 | 22311 | 0.28 | 0.19 | 0.21 | 0.69 |
| 6923 | 0.11 | 0.20 | 0.37 | 0.33 | 22383 | 1.00 | 0.39 | 0.60 | 0.02 |
| 6987 | 0.43 | 0.26 | 0.28 | 0.71 | 26305 | 0.64 | 0.74 | 0.42 | 0.92 |
| 7213 | 0.21 | 0.40 | 0.27 | 0.46 | 26519 | 0.09 | 0.00 | 0.12 | 0.12 |
| 7460 | 0.29 | 0.39 | 0.39 | 0.66 | 31238 | 0.16 | 0.09 | 0.18 | 0.17 |
| 7461 | 0.31 | 0.20 | 0.37 | 0.70 | 31245 | 0.24 | 0.18 | 0.19 | 0.35 |
| 7463 | 0.13 | 0.24 | 0.25 | 0.66 | 31253 | 0.18 | 0.27 | 0.33 | 0.54 |
| 7478 | 0.70 | 0.40 | 0.22 | 0.50 | 31289 | 0.27 | 0.12 | 0.21 | 0.12 |
| 7800 | 0.17 | 0.09 | 0.09 | 0.45 | 31291 | 0.28 | 0.07 | 0.18 | 0.11 |
| 8163 | 0.19 | 0.12 | 0.16 | 0.46 | 35349 | 1.00 | 0.38 | 0.46 | 0.26 |
| 8181 | 0.15 | 0.09 | 0.15 | 0.47 | 60961 | 0.95 | 0.81 | 0.50 | 0.55 |
| 8193 | 0.14 | 0.12 | 0.14 | 0.39 | 64971 | 0.16 | 0.02 | 0.10 | 0.28 |
| 8201 | 0.14 | 0.06 | 0.14 | 0.44 | 65084 | 0.34 | 0.58 | 0.49 | 0.29 |
| 8203 | 0.00 | 0.22 | 0.08 | 0.21 | 68066 | 0.37 | 0.20 | 0.14 | 0.36 |
| 8222 | 0.13 | 0.03 | 0.20 | 0.23 | 68171 | 0.09 | 0.02 | 0.08 | 0.22 |
| **CID** | **DILI** | **Ames** | **AOT** | **Car** | **CID** | **DILI** | **Ames** | **AOT** | **Car** |
| 8417 | 0.74 | 0.54 | 0.48 | 0.80 | 68406 | 0.09 | 0.01 | 0.08 | 0.20 |
| 8468 | 0.52 | 0.38 | 0.25 | 0.41 | 69502 | 0.85 | 0.66 | 0.89 | 0.67 |
| 9064 | 0.18 | 0.53 | 0.50 | 0.38 | 69894 | 0.69 | 0.58 | 0.46 | 0.75 |
| 10168 | 0.99 | 0.90 | 0.50 | 0.64 | 70954 | 0.28 | 0.20 | 0.22 | 0.56 |
| 10205 | 0.63 | 0.79 | 0.58 | 0.74 | 72276 | 0.18 | 0.53 | 0.50 | 0.38 |
| 72277 | 0.34 | 0.58 | 0.49 | 0.29 | 189404 | 0.23 | 0.48 | 0.40 | 0.52 |
| 73111 | 1.00 | 0.95 | 0.35 | 0.11 | 189660 | 0.44 | 0.38 | 0.40 | 0.58 |
| 73145 | 0.06 | 0.08 | 0.18 | 0.35 | 189704 | 0.26 | 0.41 | 0.31 | 0.46 |
| 73170 | 0.10 | 0.06 | 0.10 | 0.50 | 189706 | 0.38 | 0.45 | 0.39 | 0.51 |
| 73337 | 0.00 | 0.05 | 0.95 | 0.47 | 189726 | 0.78 | 0.39 | 0.35 | 0.40 |
| 74138 | 0.06 | 0.05 | 0.22 | 0.29 | 189727 | 0.79 | 0.37 | 0.34 | 0.37 |
| 75997 | 0.08 | 0.01 | 0.09 | 0.14 | 189728 | 0.68 | 0.26 | 0.24 | 0.28 |
| 76015 | 0.01 | 0.32 | 0.24 | 0.55 | 193405 | 0.10 | 0.08 | 0.29 | 0.21 |
| 76295 | 0.92 | 0.95 | 0.56 | 0.79 | 216283 | 0.76 | 0.76 | 0.51 | 0.62 |
| 77409 | 0.69 | 0.61 | 0.57 | 0.84 | 222284 | 0.10 | 0.04 | 0.09 | 0.35 |
| 79089 | 0.86 | 0.55 | 0.33 | 0.57 | 237332 | 0.85 | 0.81 | 0.43 | 0.94 |
| 80048 | 0.32 | 0.25 | 0.40 | 0.66 | 259846 | 0.07 | 0.04 | 0.08 | 0.56 |
| 81696 | 0.23 | 0.64 | 0.28 | 0.29 | 267137 | 0.72 | 0.43 | 0.22 | 0.48 |
| 83412 | 0.78 | 0.60 | 0.25 | 0.67 | 283510 | 0.07 | 0.04 | 0.16 | 0.28 |
| 91440 | 1.00 | 0.95 | 0.35 | 0.11 | 304040 | 0.85 | 0.41 | 0.19 | 0.42 |
| 92139 | 0.11 | 0.26 | 0.20 | 0.43 | 330573 | 0.28 | 0.18 | 0.23 | 0.64 |
| 92221 | 0.48 | 0.17 | 0.22 | 0.54 | 334704 | 0.64 | 0.74 | 0.42 | 0.92 |
| 94162 | 0.27 | 0.25 | 0.39 | 0.35 | 361512 | 0.87 | 0.92 | 0.37 | 0.82 |
| 94221 | 0.28 | 0.18 | 0.23 | 0.64 | 400073 | 0.70 | 0.33 | 0.28 | 0.70 |
| 94249 | 0.25 | 0.23 | 0.14 | 0.55 | 439242 | 0.33 | 0.73 | 0.18 | 0.09 |
| 94403 | 0.11 | 0.20 | 0.07 | 0.60 | 439357 | 0.23 | 0.64 | 0.28 | 0.29 |
| 98570 | 0.44 | 0.72 | 0.77 | 0.92 | 439503 | 0.09 | 0.48 | 0.08 | 0.32 |
| 100017 | 0.73 | 0.25 | 0.87 | 0.32 | 439531 | 0.43 | 0.83 | 0.17 | 0.04 |
| 101977 | 0.09 | 0.17 | 0.08 | 0.45 | 440917 | 0.28 | 0.19 | 0.21 | 0.69 |
| 104285 | 1.00 | 0.37 | 0.56 | 0.02 | 440967 | 0.32 | 0.07 | 0.13 | 0.60 |
| 104884 | 0.81 | 0.28 | 0.88 | 0.22 | 440968 | 0.33 | 0.02 | 0.05 | 0.63 |
| 106648 | 0.79 | 0.64 | 0.59 | 0.81 | 441005 | 0.57 | 0.21 | 0.24 | 0.58 |
| 107526 | 0.00 | 0.50 | 0.03 | 0.11 | 442015 | 0.93 | 0.30 | 0.23 | 0.76 |
| 107936 | 0.74 | 0.70 | 0.54 | 0.80 | 442068 | 0.94 | 0.44 | 0.36 | 0.48 |
| 108058 | 0.73 | 0.25 | 0.87 | 0.31 | 442127 | 0.55 | 0.48 | 0.53 | 0.77 |
| 122738 | 0.24 | 0.47 | 0.70 | 0.09 | 442153 | 0.52 | 0.77 | 0.85 | 0.46 |
| 125213 | 0.13 | 0.49 | 0.16 | 0.49 | 442154 | 0.11 | 0.45 | 0.46 | 0.47 |
| 125468 | 0.57 | 0.35 | 0.39 | 0.52 | 442359 | 0.37 | 0.13 | 0.26 | 0.48 |
| 126566 | 0.91 | 0.30 | 0.51 | 0.44 | 442731 | 0.91 | 0.89 | 0.35 | 0.59 |
| 129754 | 0.30 | 0.19 | 0.58 | 0.18 | 443158 | 0.07 | 0.37 | 0.19 | 0.61 |
| 138824 | 0.02 | 0.17 | 0.06 | 0.41 | 443639 | 0.11 | 0.45 | 0.46 | 0.47 |
| 157277 | 0.53 | 0.33 | 0.86 | 0.46 | 444539 | 0.25 | 0.42 | 0.27 | 0.16 |
| 159931 | 0.05 | 0.11 | 0.07 | 0.48 | 444899 | 0.00 | 0.46 | 0.05 | 0.12 |
| 167718 | 0.00 | 0.08 | 0.72 | 0.34 | 445638 | 0.01 | 0.08 | 0.07 | 0.12 |
| 173183 | 0.08 | 0.05 | 0.12 | 0.37 | 445639 | 0.01 | 0.08 | 0.07 | 0.17 |
| **CID** | **DILI** | **Ames** | **AOT** | **Car** | **CID** | **DILI** | **Ames** | **AOT** | **Car** |
| 176920 | 0.20 | 0.45 | 0.55 | 0.36 | 480764 | 0.44 | 0.72 | 0.81 | 0.51 |
| 176996 | 0.66 | 0.23 | 0.89 | 0.28 | 484588 | 0.63 | 0.81 | 0.90 | 0.42 |
| 177090 | 0.18 | 0.26 | 0.26 | 0.56 | 500060 | 0.85 | 0.21 | 0.70 | 0.48 |
| 178770 | 0.84 | 0.19 | 0.85 | 0.45 | 518975 | 0.29 | 0.51 | 0.35 | 0.75 |
| 180429 | 0.25 | 0.65 | 0.67 | 0.74 | 520895 | 0.99 | 0.21 | 0.51 | 0.21 |
| 180932 | 0.94 | 0.44 | 0.30 | 0.25 | 521941 | 0.99 | 0.36 | 0.50 | 0.15 |
| 184937 | 0.73 | 0.23 | 0.55 | 0.54 | 522458 | 0.98 | 0.30 | 0.50 | 0.23 |
| 188289 | 0.93 | 0.30 | 0.23 | 0.76 | 525330 | 1.00 | 0.46 | 0.65 | 0.07 |
| 189403 | 0.25 | 0.49 | 0.41 | 0.52 | 529904 | 1.00 | 0.43 | 0.57 | 0.15 |
| 575174 | 0.10 | 0.08 | 0.29 | 0.21 | 5288377 | 0.73 | 0.44 | 0.28 | 0.09 |
| 584354 | 0.52 | 0.63 | 0.38 | 0.77 | 5315396 | 0.80 | 0.71 | 0.70 | 0.68 |
| 595524 | 0.75 | 0.59 | 0.38 | 0.77 | 5315851 | 0.80 | 0.85 | 0.32 | 0.60 |
| 600671 | 0.55 | 0.48 | 0.53 | 0.77 | 5316525 | 0.78 | 0.67 | 0.71 | 0.75 |
| 611513 | 0.76 | 0.76 | 0.51 | 0.62 | 5316800 | 0.85 | 0.90 | 0.48 | 0.78 |
| 636663 | 0.70 | 0.83 | 0.34 | 0.90 | 5316860 | 0.18 | 0.63 | 0.21 | 0.31 |
| 636837 | 0.65 | 0.20 | 0.56 | 0.37 | 5316891 | 0.71 | 0.71 | 0.56 | 0.83 |
| 638072 | 0.01 | 0.01 | 0.01 | 0.16 | 5318565 | 0.62 | 0.48 | 0.43 | 0.74 |
| 643654 | 0.04 | 0.53 | 0.68 | 0.52 | 5318767 | 0.75 | 0.73 | 0.42 | 0.18 |
| 1550607 | 0.70 | 0.33 | 0.28 | 0.70 | 5319336 | 0.85 | 0.13 | 0.71 | 0.62 |
| 1713001 | 0.13 | 0.11 | 0.09 | 0.50 | 5319406 | 0.52 | 0.27 | 0.32 | 0.64 |
| 1742210 | 0.36 | 0.35 | 0.39 | 0.61 | 5319500 | 0.76 | 0.56 | 0.55 | 0.65 |
| 1795390 | 0.70 | 0.33 | 0.28 | 0.70 | 5319706 | 0.00 | 0.54 | 0.11 | 0.42 |
| 1810796 | 0.02 | 0.15 | 0.06 | 0.39 | 5319765 | 1.00 | 0.47 | 0.60 | 0.04 |
| 2723872 | 0.05 | 0.58 | 0.15 | 0.40 | 5320171 | 0.97 | 0.56 | 0.82 | 0.09 |
| 3034112 | 0.67 | 0.23 | 0.54 | 0.59 | 5320250 | 0.27 | 0.60 | 0.52 | 0.60 |
| 3084213 | 0.49 | 0.48 | 0.19 | 0.75 | 5352019 | 1.00 | 0.88 | 0.55 | 0.13 |
| 4482272 | 0.81 | 0.28 | 0.88 | 0.22 | 5353609 | 0.07 | 0.62 | 0.25 | 0.37 |
| 5260170 | 0.68 | 0.26 | 0.89 | 0.19 | 5363269 | 0.01 | 0.08 | 0.05 | 0.26 |
| 5280343 | 0.78 | 0.59 | 0.48 | 0.60 | 5367462 | 0.00 | 0.54 | 0.11 | 0.42 |
| 5280435 | 0.01 | 0.10 | 0.03 | 0.28 | 5459840 | 0.01 | 0.26 | 0.11 | 0.43 |
| 5280443 | 0.74 | 0.62 | 0.52 | 0.79 | 5742590 | 0.16 | 0.08 | 0.15 | 0.17 |
| 5280450 | 0.00 | 0.13 | 0.06 | 0.10 | 6324923 | 0.90 | 0.89 | 0.33 | 0.58 |
| 5280459 | 0.73 | 0.63 | 0.43 | 0.29 | 6434062 | 0.27 | 0.58 | 0.45 | 0.60 |
| 5280460 | 0.73 | 0.59 | 0.49 | 0.79 | 6437066 | 0.81 | 0.28 | 0.88 | 0.22 |
| 5280489 | 0.12 | 0.84 | 0.86 | 0.87 | 6442484 | 0.93 | 0.55 | 0.87 | 0.11 |
| 5280537 | 0.13 | 0.49 | 0.16 | 0.49 | 6442906 | 0.42 | 0.44 | 0.35 | 0.25 |
| 5280704 | 0.88 | 0.66 | 0.31 | 0.51 | 6450230 | 0.49 | 0.48 | 0.19 | 0.75 |
| 5280794 | 0.05 | 0.02 | 0.11 | 0.19 | 6451151 | 0.88 | 0.58 | 0.24 | 0.67 |
| 5280804 | 0.66 | 0.60 | 0.29 | 0.27 | 6451598 | 0.35 | 0.35 | 0.27 | 0.50 |
| 5280805 | 0.81 | 0.75 | 0.41 | 0.11 | 6453932 | 0.12 | 0.09 | 0.12 | 0.03 |
| 5280862 | 0.67 | 0.54 | 0.47 | 0.77 | 6482976 | 0.53 | 0.45 | 0.12 | 0.41 |
| 5280863 | 0.70 | 0.55 | 0.49 | 0.72 | 6918743 | 0.27 | 0.48 | 0.09 | 0.22 |
| 5280934 | 0.01 | 0.38 | 0.09 | 0.23 | 9548705 | 0.31 | 0.06 | 0.11 | 0.72 |
| 5281119 | 0.01 | 0.08 | 0.06 | 0.13 | 9796891 | 0.75 | 0.59 | 0.46 | 0.73 |
| 5281303 | 0.64 | 0.97 | 0.85 | 0.25 | 10031185 | 0.41 | 0.31 | 0.29 | 0.26 |
| **CID** | **DILI** | **Ames** | **AOT** | **Car** | **CID** | **DILI** | **Ames** | **AOT** | **Car** |
| 5281310 | 0.81 | 0.28 | 0.88 | 0.22 | 10065647 | 0.95 | 0.87 | 0.65 | 0.91 |
| 5281426 | 0.67 | 0.61 | 0.48 | 0.83 | 10098738 | 0.88 | 0.29 | 0.34 | 0.37 |
| 5281515 | 0.38 | 0.17 | 0.21 | 0.56 | 10108651 | 0.46 | 0.37 | 0.14 | 0.45 |
| 5281520 | 0.24 | 0.06 | 0.21 | 0.61 | 10181133 | 0.03 | 0.11 | 0.28 | 0.14 |
| 5281522 | 0.38 | 0.17 | 0.21 | 0.56 | 10212035 | 0.06 | 0.00 | 0.03 | 0.04 |
| 5281553 | 0.27 | 0.60 | 0.52 | 0.60 | 10239837 | 0.14 | 0.42 | 0.70 | 0.12 |
| 5281672 | 0.84 | 0.66 | 0.44 | 0.50 | 10263440 | 0.29 | 0.37 | 0.29 | 0.71 |
| 5281876 | 0.62 | 0.97 | 0.86 | 0.17 | 10348278 | 0.41 | 0.31 | 0.29 | 0.26 |
| 5282102 | 0.57 | 0.57 | 0.29 | 0.40 | 10392456 | 0.88 | 0.29 | 0.34 | 0.37 |
| 5282822 | 0.01 | 0.38 | 0.09 | 0.23 | 10438246 | 0.25 | 0.31 | 0.24 | 0.22 |
| 5283640 | 0.07 | 0.03 | 0.14 | 0.31 | 10442609 | 0.51 | 0.57 | 0.28 | 0.53 |
| 5284421 | 0.00 | 0.22 | 0.08 | 0.21 | 10483388 | 0.41 | 0.31 | 0.29 | 0.26 |
| 5288340 | 0.46 | 0.71 | 0.25 | 0.17 | 10505484 | 0.66 | 0.23 | 0.89 | 0.28 |
| 10601920 | 0.05 | 0.20 | 0.14 | 0.48 | 13370049 | 0.19 | 0.15 | 0.49 | 0.27 |
| 10628287 | 0.69 | 0.98 | 0.80 | 0.32 | 13458955 | 0.06 | 0.03 | 0.07 | 0.37 |
| 10767085 | 0.01 | 0.26 | 0.11 | 0.43 | 13821181 | 0.68 | 0.69 | 0.28 | 0.47 |
| 10884852 | 0.56 | 0.37 | 0.07 | 0.37 | 13834020 | 0.84 | 0.19 | 0.85 | 0.45 |
| 10906239 | 0.76 | 0.14 | 0.78 | 0.43 | 13856086 | 0.88 | 0.60 | 0.65 | 0.59 |
| 10977864 | 0.39 | 0.52 | 0.08 | 0.77 | 13856092 | 0.87 | 0.07 | 0.38 | 0.11 |
| 11077057 | 0.45 | 0.23 | 0.09 | 0.61 | 13858079 | 0.93 | 0.35 | 0.21 | 0.46 |
| 11087935 | 0.36 | 0.52 | 0.72 | 0.62 | 13875741 | 0.77 | 0.25 | 0.75 | 0.52 |
| 11088324 | 0.75 | 0.59 | 0.46 | 0.73 | 13875755 | 0.79 | 0.33 | 0.63 | 0.31 |
| 11095397 | 0.94 | 0.85 | 0.59 | 0.73 | 13875766 | 0.65 | 0.50 | 0.69 | 0.72 |
| 11119228 | 0.16 | 0.34 | 0.29 | 0.40 | 13875774 | 0.66 | 0.34 | 0.37 | 0.44 |
| 11192900 | 0.68 | 0.77 | 0.03 | 0.89 | 13875775 | 0.78 | 0.52 | 0.78 | 0.14 |
| 11209134 | 0.74 | 0.56 | 0.26 | 0.42 | 13965525 | 0.53 | 0.39 | 0.31 | 0.73 |
| 11243273 | 0.92 | 0.76 | 0.30 | 0.90 | 14015932 | 0.02 | 0.19 | 0.88 | 0.12 |
| 11334829 | 0.24 | 0.31 | 0.24 | 0.28 | 14015943 | 0.09 | 0.34 | 0.66 | 0.16 |
| 11403749 | 0.46 | 0.37 | 0.14 | 0.45 | 14015944 | 0.09 | 0.34 | 0.66 | 0.16 |
| 11482406 | 0.56 | 0.64 | 0.43 | 0.82 | 14015948 | 0.15 | 0.39 | 0.66 | 0.12 |
| 11767849 | 0.01 | 0.01 | 0.01 | 0.01 | 14015949 | 0.15 | 0.39 | 0.66 | 0.12 |
| 11770062 | 0.28 | 0.20 | 0.19 | 0.60 | 14015956 | 0.13 | 0.61 | 0.33 | 0.29 |
| 11798426 | 0.74 | 0.53 | 0.68 | 0.75 | 14015959 | 0.14 | 0.42 | 0.70 | 0.12 |
| 11813223 | 0.71 | 0.63 | 0.19 | 0.50 | 14015964 | 0.02 | 0.23 | 0.78 | 0.39 |
| 11818411 | 0.16 | 0.27 | 0.14 | 0.38 | 14015965 | 0.02 | 0.23 | 0.78 | 0.39 |
| 11870456 | 0.10 | 0.04 | 0.09 | 0.35 | 14015967 | 0.03 | 0.21 | 0.79 | 0.37 |
| 12004512 | 0.81 | 0.21 | 0.51 | 0.59 | 14015968 | 0.03 | 0.21 | 0.79 | 0.37 |
| 12011153 | 0.55 | 0.24 | 0.57 | 0.58 | 14136864 | 0.80 | 0.58 | 0.93 | 0.30 |
| 12046149 | 0.28 | 0.20 | 0.19 | 0.60 | 14194023 | 0.69 | 0.42 | 0.89 | 0.43 |
| 12069125 | 0.69 | 0.32 | 0.69 | 0.60 | 14194109 | 0.85 | 0.29 | 0.22 | 0.38 |
| 12072821 | 0.63 | 0.27 | 0.53 | 0.34 | 14213968 | 0.72 | 0.59 | 0.51 | 0.77 |
| 12272224 | 0.79 | 0.73 | 0.50 | 0.83 | 14218028 | 0.53 | 0.77 | 0.77 | 0.55 |
| 12302222 | 0.34 | 0.30 | 0.13 | 0.72 | 14287157 | 0.33 | 0.33 | 0.25 | 0.47 |
| 12302226 | 0.34 | 0.30 | 0.13 | 0.72 | 14287159 | 0.26 | 0.33 | 0.40 | 0.55 |
| 12302227 | 0.34 | 0.30 | 0.13 | 0.72 | 14309784 | 0.67 | 0.50 | 0.09 | 0.67 |
| **CID** | **DILI** | **Ames** | **AOT** | **Car** | **CID** | **DILI** | **Ames** | **AOT** | **Car** |
| 12302228 | 0.34 | 0.30 | 0.13 | 0.72 | 14458886 | 0.68 | 0.26 | 0.89 | 0.19 |
| 12302243 | 0.29 | 0.50 | 0.28 | 0.65 | 14467538 | 0.72 | 0.33 | 0.91 | 0.23 |
| 12303902 | 0.61 | 0.13 | 0.17 | 0.66 | 14492795 | 0.74 | 0.79 | 0.89 | 0.56 |
| 12305246 | 0.48 | 0.21 | 0.13 | 0.79 | 14563366 | 0.91 | 0.41 | 0.80 | 0.10 |
| 12305247 | 0.48 | 0.21 | 0.13 | 0.79 | 14752824 | 0.73 | 0.48 | 0.25 | 0.22 |
| 12306053 | 0.62 | 0.19 | 0.21 | 0.54 | 14807789 | 0.64 | 0.54 | 0.24 | 0.59 |
| 12308714 | 0.85 | 0.21 | 0.70 | 0.48 | 14845542 | 0.05 | 0.03 | 0.10 | 0.20 |
| 12308716 | 0.85 | 0.21 | 0.70 | 0.48 | 14845550 | 0.48 | 0.21 | 0.39 | 0.38 |
| 12309449 | 0.28 | 0.15 | 0.46 | 0.63 | 15008366 | 0.57 | 0.52 | 0.31 | 0.76 |
| 12309491 | 0.11 | 0.45 | 0.46 | 0.47 | 15108321 | 0.08 | 0.66 | 0.22 | 0.34 |
| 12310089 | 0.85 | 0.58 | 0.36 | 0.35 | 15215479 | 0.80 | 0.23 | 0.34 | 0.68 |
| 12312690 | 0.01 | 0.18 | 0.09 | 0.37 | 15485379 | 0.62 | 0.33 | 0.85 | 0.54 |
| 12313023 | 0.51 | 0.19 | 0.16 | 0.63 | 15560114 | 0.49 | 0.48 | 0.19 | 0.75 |
| 12313376 | 0.73 | 0.25 | 0.87 | 0.32 | 15560276 | 0.41 | 0.24 | 0.30 | 0.65 |
| 12376292 | 0.28 | 0.48 | 0.27 | 0.54 | 15560423 | 0.28 | 0.04 | 0.17 | 0.23 |
| 12443210 | 0.61 | 0.59 | 0.26 | 0.38 | 15768008 | 0.97 | 0.62 | 0.36 | 0.55 |
| 12677834 | 1.00 | 0.11 | 0.31 | 0.11 | 15768009 | 0.97 | 0.62 | 0.36 | 0.55 |
| 15840160 | 0.10 | 0.25 | 0.17 | 0.11 | 44583637 | 0.98 | 0.92 | 0.49 | 0.81 |
| 15885442 | 0.52 | 0.20 | 0.25 | 0.27 | 44584063 | 0.64 | 0.97 | 0.85 | 0.25 |
| 15885443 | 0.69 | 0.06 | 0.42 | 0.11 | 44614139 | 0.21 | 0.14 | 0.34 | 0.14 |
| 15934443 | 0.44 | 0.42 | 0.24 | 0.39 | 44631202 | 0.73 | 0.25 | 0.87 | 0.32 |
| 16126804 | 0.64 | 0.97 | 0.85 | 0.25 | 44715635 | 0.80 | 0.16 | 0.89 | 0.34 |
| 16396350 | 0.38 | 0.26 | 0.26 | 0.65 | 45103626 | 0.99 | 0.90 | 0.53 | 0.94 |
| 16722121 | 0.83 | 0.97 | 0.87 | 0.28 | 45268397 | 0.74 | 0.79 | 0.89 | 0.56 |
| 16722130 | 0.86 | 0.97 | 0.85 | 0.37 | 45272307 | 0.56 | 0.59 | 0.20 | 0.77 |
| 20056138 | 0.59 | 0.42 | 0.41 | 0.42 | 46173826 | 0.06 | 0.11 | 0.14 | 0.16 |
| 20488062 | 0.13 | 0.13 | 0.29 | 0.97 | 46201020 | 0.95 | 0.84 | 0.40 | 0.89 |
| 20976991 | 0.09 | 0.00 | 0.07 | 0.18 | 46211187 | 0.36 | 0.53 | 0.30 | 0.46 |
| 21581301 | 0.80 | 0.97 | 0.77 | 0.39 | 46224590 | 0.45 | 0.70 | 0.45 | 0.13 |
| 21581584 | 0.98 | 0.28 | 0.66 | 0.42 | 46919586 | 0.38 | 0.36 | 0.61 | 0.26 |
| 21592304 | 0.64 | 0.42 | 0.54 | 0.40 | 49863985 | 0.64 | 0.33 | 0.80 | 0.62 |
| 21594203 | 0.05 | 0.03 | 0.14 | 0.17 | 49864004 | 0.49 | 0.31 | 0.40 | 0.22 |
| 21597549 | 0.73 | 0.71 | 0.56 | 0.67 | 49864005 | 0.14 | 0.18 | 0.38 | 0.10 |
| 21600035 | 0.48 | 0.03 | 0.05 | 0.06 | 49864006 | 0.67 | 0.18 | 0.17 | 0.08 |
| 21603566 | 0.05 | 0.03 | 0.14 | 0.17 | 51402807 | 0.66 | 0.60 | 0.29 | 0.27 |
| 21625636 | 0.93 | 0.39 | 0.41 | 0.41 | 51694242 | 0.26 | 0.41 | 0.31 | 0.46 |
| 21626436 | 0.07 | 0.29 | 0.81 | 0.04 | 52951756 | 0.84 | 0.13 | 0.68 | 0.36 |
| 21632833 | 0.43 | 0.32 | 0.42 | 0.48 | 52951892 | 0.80 | 0.28 | 0.64 | 0.58 |
| 21632843 | 0.06 | 0.17 | 0.22 | 0.44 | 52951893 | 0.64 | 0.12 | 0.67 | 0.60 |
| 21725519 | 0.60 | 0.95 | 0.86 | 0.09 | 52951894 | 0.80 | 0.28 | 0.64 | 0.58 |
| 21725521 | 0.84 | 0.97 | 0.91 | 0.27 | 52951895 | 0.81 | 0.07 | 0.43 | 0.34 |
| 21725522 | 0.84 | 0.97 | 0.89 | 0.24 | 52952011 | 0.71 | 0.26 | 0.33 | 0.50 |
| 23256847 | 0.80 | 0.98 | 0.90 | 0.18 | 52952012 | 0.58 | 0.32 | 0.44 | 0.37 |
| 24796982 | 0.60 | 0.95 | 0.86 | 0.09 | 52952013 | 0.94 | 0.39 | 0.81 | 0.31 |
| 24867638 | 0.90 | 0.70 | 0.63 | 0.56 | 52952112 | 0.81 | 0.07 | 0.33 | 0.57 |
| **CID** | **DILI** | **Ames** | **AOT** | **Car** | **CID** | **DILI** | **Ames** | **AOT** | **Car** |
| 24879663 | 0.68 | 0.56 | 0.18 | 0.47 | 52952113 | 0.55 | 0.24 | 0.57 | 0.58 |
| 25769005 | 0.90 | 0.39 | 0.24 | 0.38 | 52952216 | 0.61 | 0.32 | 0.86 | 0.44 |
| 40469553 | 0.67 | 0.23 | 0.54 | 0.59 | 52952322 | 0.80 | 0.31 | 0.81 | 0.04 |
| 40469561 | 0.81 | 0.21 | 0.51 | 0.59 | 52952323 | 0.77 | 0.15 | 0.81 | 0.13 |
| 42433469 | 0.18 | 0.30 | 0.09 | 0.82 | 52952435 | 0.84 | 0.74 | 0.55 | 0.60 |
| 42607958 | 0.45 | 0.77 | 0.63 | 0.71 | 52952436 | 0.78 | 0.38 | 0.46 | 0.51 |
| 42608071 | 0.53 | 0.68 | 0.79 | 0.64 | 52952437 | 0.78 | 0.38 | 0.46 | 0.51 |
| 42608075 | 0.79 | 0.75 | 0.75 | 0.69 | 53438729 | 0.99 | 0.78 | 0.50 | 0.66 |
| 42608116 | 0.59 | 0.77 | 0.74 | 0.38 | 54580354 | 0.81 | 0.08 | 0.52 | 0.50 |
| 42626428 | 0.09 | 0.52 | 0.54 | 0.62 | 54580355 | 0.82 | 0.53 | 0.17 | 0.42 |
| 44146779 | 0.62 | 0.69 | 0.41 | 0.87 | 54581351 | 0.54 | 0.72 | 0.82 | 0.09 |
| 44259428 | 0.87 | 0.80 | 0.37 | 0.08 | 54581352 | 0.84 | 0.74 | 0.55 | 0.60 |
| 44566526 | 0.94 | 0.39 | 0.81 | 0.31 | 54583360 | 0.54 | 0.72 | 0.82 | 0.09 |
| 44567124 | 0.09 | 0.05 | 0.20 | 0.19 | 54583361 | 0.68 | 0.26 | 0.89 | 0.19 |
| 44567142 | 0.09 | 0.11 | 0.18 | 0.30 | 54585273 | 0.65 | 0.35 | 0.86 | 0.39 |
| 44575502 | 0.14 | 0.79 | 0.06 | 0.10 | 54586223 | 0.84 | 0.96 | 0.88 | 0.09 |
| 44575793 | 0.21 | 0.18 | 0.46 | 0.21 | 54587249 | 0.81 | 0.28 | 0.88 | 0.22 |
| 44579695 | 0.63 | 0.64 | 0.26 | 0.45 | 54670067 | 0.38 | 0.31 | 0.05 | 0.36 |
| 44579696 | 0.48 | 0.51 | 0.15 | 0.46 | 54758525 | 0.05 | 0.22 | 0.29 | 0.22 |
| 44579743 | 0.80 | 0.63 | 0.31 | 0.19 | 54758526 | 0.14 | 0.32 | 0.46 | 0.16 |
| 44579744 | 0.80 | 0.63 | 0.31 | 0.19 | 56841069 | 0.53 | 0.10 | 0.21 | 0.44 |
| 56958440 | 0.78 | 0.80 | 0.57 | 0.76 | 90470576 | 0.12 | 0.46 | 0.45 | 0.33 |
| 56958777 | 0.54 | 0.63 | 0.44 | 0.76 | 91884898 | 0.91 | 0.30 | 0.20 | 0.29 |
| 70689030 | 0.05 | 0.66 | 0.39 | 0.84 | 91886694 | 0.84 | 0.29 | 0.92 | 0.13 |
| 70697879 | 0.87 | 0.66 | 0.43 | 0.40 | 100926540 | 0.46 | 0.56 | 0.35 | 0.47 |
| 70697889 | 0.74 | 0.53 | 0.68 | 0.75 | 100926541 | 0.55 | 0.59 | 0.33 | 0.18 |
| 71338636 | 0.99 | 0.32 | 0.59 | 0.21 | 100996181 | 0.03 | 0.14 | 0.13 | 0.53 |
| 71413104 | 0.90 | 0.91 | 0.32 | 0.84 | 101026859 | 0.94 | 0.26 | 0.59 | 0.20 |
| 71584574 | 0.61 | 0.36 | 0.11 | 0.77 | 101034965 | 0.39 | 0.38 | 0.53 | 0.48 |
| 71584688 | 0.58 | 0.25 | 0.09 | 0.72 | 101153492 | 0.42 | 0.41 | 0.25 | 0.05 |
| 71584689 | 0.54 | 0.13 | 0.08 | 0.66 | 101153494 | 0.61 | 0.26 | 0.37 | 0.16 |
| 71584690 | 0.02 | 0.31 | 0.04 | 0.36 | 101280240 | 0.48 | 0.25 | 0.80 | 0.53 |
| 71584691 | 0.04 | 0.16 | 0.04 | 0.38 | 101289833 | 0.66 | 0.19 | 0.76 | 0.50 |
| 71717738 | 0.34 | 0.49 | 0.27 | 0.75 | 101355584 | 0.97 | 0.30 | 0.62 | 0.11 |
| 71720036 | 0.74 | 0.68 | 0.63 | 0.78 | 101529198 | 0.28 | 0.35 | 0.44 | 0.43 |
| 72738894 | 0.73 | 0.48 | 0.25 | 0.22 | 101570727 | 0.83 | 0.72 | 0.66 | 0.75 |
| 73076982 | 0.92 | 0.50 | 0.69 | 0.46 | 101602319 | 0.05 | 0.08 | 0.20 | 0.36 |
| 73187989 | 0.68 | 0.69 | 0.28 | 0.47 | 101602320 | 0.52 | 0.23 | 0.44 | 0.26 |
| 73356511 | 0.84 | 0.19 | 0.85 | 0.45 | 101602321 | 0.53 | 0.20 | 0.38 | 0.19 |
| 73797339 | 0.48 | 0.03 | 0.05 | 0.06 | 101634707 | 0.53 | 0.33 | 0.86 | 0.46 |
| 73804953 | 0.80 | 0.97 | 0.77 | 0.39 | 101676207 | 0.92 | 0.17 | 0.22 | 0.23 |
| 73813111 | 0.83 | 0.40 | 0.80 | 0.29 | 101676208 | 0.76 | 0.18 | 0.23 | 0.25 |
| 73824950 | 0.60 | 0.95 | 0.86 | 0.09 | 101676711 | 0.92 | 0.17 | 0.22 | 0.23 |
| 73824951 | 0.68 | 0.98 | 0.78 | 0.10 | 101915817 | 0.81 | 0.39 | 0.44 | 0.48 |
| 73824953 | 0.84 | 0.97 | 0.89 | 0.24 | 101916313 | 0.91 | 0.22 | 0.23 | 0.36 |
| **CID** | **DILI** | **Ames** | **AOT** | **Car** | **CID** | **DILI** | **Ames** | **AOT** | **Car** |
| 74073445 | 0.54 | 0.49 | 0.40 | 0.43 | 101919043 | 0.98 | 0.93 | 0.89 | 0.22 |
| 74075981 | 0.03 | 0.08 | 0.19 | 0.17 | 101936072 | 0.72 | 0.33 | 0.91 | 0.23 |
| 74336648 | 0.70 | 0.44 | 0.12 | 0.58 | 101999884 | 0.60 | 0.98 | 0.86 | 0.27 |
| 74978378 | 0.89 | 0.75 | 0.26 | 0.13 | 102034873 | 0.45 | 0.58 | 0.56 | 0.50 |
| 75050399 | 0.63 | 0.64 | 0.26 | 0.45 | 102063005 | 0.69 | 0.98 | 0.80 | 0.32 |
| 75050400 | 0.48 | 0.51 | 0.15 | 0.46 | 102149247 | 0.37 | 0.54 | 0.60 | 0.32 |
| 75111036 | 0.11 | 0.84 | 0.11 | 0.43 | 102285347 | 0.71 | 0.15 | 0.90 | 0.35 |
| 75971805 | 0.64 | 0.12 | 0.67 | 0.60 | 102316534 | 0.29 | 0.05 | 0.24 | 0.12 |
| 75971891 | 0.81 | 0.07 | 0.33 | 0.57 | 102316535 | 0.33 | 0.15 | 0.18 | 0.24 |
| 75972015 | 0.84 | 0.74 | 0.55 | 0.60 | 118855989 | 0.94 | 0.44 | 0.30 | 0.25 |
| 76152128 | 0.82 | 0.53 | 0.17 | 0.42 | 123981968 | 0.83 | 0.72 | 0.66 | 0.75 |
| 76153908 | 0.65 | 0.35 | 0.86 | 0.39 | 124305339 | 0.18 | 0.30 | 0.09 | 0.82 |
| 76311433 | 0.15 | 0.11 | 0.26 | 0.20 | 124629574 | 0.73 | 0.25 | 0.87 | 0.32 |
| 76316558 | 0.83 | 0.15 | 0.76 | 0.48 | 129010007 | 0.91 | 0.86 | 0.50 | 0.19 |
| 76316561 | 0.86 | 0.62 | 0.93 | 0.22 | 129712290 | 0.83 | 0.37 | 0.73 | 0.07 |
| 76317961 | 0.83 | 0.15 | 0.76 | 0.48 | 131676058 | 0.81 | 0.28 | 0.88 | 0.22 |
| 76327056 | 0.83 | 0.97 | 0.87 | 0.28 | 131698851 | 0.64 | 0.97 | 0.85 | 0.25 |
| 76685092 | 0.69 | 0.25 | 0.50 | 0.30 | 131705161 | 0.18 | 0.30 | 0.09 | 0.82 |
| 77916018 | 0.87 | 0.66 | 0.43 | 0.40 | 131875206 | 0.60 | 0.06 | 0.51 | 0.24 |
| 78148404 | 0.80 | 0.09 | 0.74 | 0.15 | 132990894 | 0.43 | 0.83 | 0.17 | 0.04 |
| 78157935 | 0.74 | 0.68 | 0.63 | 0.78 | 135369651 | 0.16 | 0.06 | 0.12 | 0.31 |
| 78167002 | 0.05 | 0.03 | 0.05 | 0.15 | 136360357 | 0.93 | 0.79 | 0.67 | 0.93 |
| 85302768 | 0.77 | 0.63 | 0.91 | 0.11 | 138115244 | 0.73 | 0.25 | 0.87 | 0.32 |
| 85596052 | 0.07 | 0.00 | 0.05 | 0.11 | 139057051 | 0.15 | 0.04 | 0.12 | 0.28 |
| 85776164 | 0.07 | 0.29 | 0.24 | 0.32 | 139057501 | 0.84 | 0.19 | 0.85 | 0.45 |
| 152743364 | 0.12 | 0.84 | 0.86 | 0.87 | 162917693 | 0.86 | 0.62 | 0.77 | 0.32 |
| 154496877 | 0.37 | 0.26 | 0.23 | 0.68 | 162918748 | 0.64 | 0.33 | 0.80 | 0.62 |
| 154497094 | 0.73 | 0.25 | 0.87 | 0.32 | 162920427 | 0.81 | 0.95 | 0.93 | 0.03 |
| 154497120 | 0.15 | 0.11 | 0.26 | 0.20 | 162920428 | 0.81 | 0.95 | 0.93 | 0.03 |
| 154497153 | 0.18 | 0.26 | 0.18 | 0.66 | 162920595 | 0.37 | 0.03 | 0.02 | 0.26 |
| 154497731 | 0.26 | 0.20 | 0.29 | 0.48 | 162921038 | 0.83 | 0.50 | 0.86 | 0.56 |
| 162819794 | 0.04 | 0.16 | 0.04 | 0.38 | 162921039 | 0.83 | 0.50 | 0.86 | 0.56 |
| 162842140 | 0.30 | 0.40 | 0.32 | 0.53 | 162921836 | 0.20 | 0.15 | 0.20 | 0.26 |
| 162845059 | 0.01 | 0.11 | 0.08 | 0.13 | 162921838 | 0.20 | 0.15 | 0.20 | 0.26 |
| 162845288 | 0.45 | 0.40 | 0.62 | 0.84 | 162922533 | 0.78 | 0.52 | 0.78 | 0.14 |
| 162847066 | 0.34 | 0.49 | 0.27 | 0.75 | 162922614 | 0.15 | 0.86 | 0.23 | 0.31 |
| 162847297 | 0.37 | 0.19 | 0.32 | 0.27 | 162925476 | 0.71 | 0.44 | 0.31 | 0.83 |
| 162847298 | 0.37 | 0.19 | 0.32 | 0.27 | 162925999 | 0.64 | 0.42 | 0.54 | 0.40 |
| 162853040 | 0.68 | 0.26 | 0.89 | 0.19 | 162934403 | 0.58 | 0.07 | 0.22 | 0.18 |
| 162853682 | 0.78 | 0.51 | 0.71 | 0.19 | 162935328 | 0.98 | 0.45 | 0.24 | 0.39 |
| 162853683 | 0.78 | 0.51 | 0.71 | 0.19 | 162935329 | 0.98 | 0.45 | 0.24 | 0.39 |
| 162857811 | 0.44 | 0.32 | 0.10 | 0.42 | 162938666 | 0.53 | 0.56 | 0.56 | 0.53 |
| 162859638 | 0.16 | 0.10 | 0.28 | 0.24 | 162940876 | 0.85 | 0.60 | 0.20 | 0.52 |
| 162867112 | 0.65 | 0.50 | 0.40 | 0.75 | 162944734 | 0.26 | 0.79 | 0.20 | 0.32 |
| 162867118 | 0.26 | 0.48 | 0.27 | 0.80 | 162946727 | 0.91 | 0.36 | 0.15 | 0.41 |
| **CID** | **DILI** | **Ames** | **AOT** | **Car** | **CID** | **DILI** | **Ames** | **AOT** | **Car** |
| 162867119 | 0.63 | 0.58 | 0.52 | 0.79 | 162949507 | 0.68 | 0.36 | 0.66 | 0.15 |
| 162867134 | 0.95 | 0.06 | 0.06 | 0.04 | 162951523 | 0.88 | 0.64 | 0.96 | 0.13 |
| 162867348 | 0.80 | 0.63 | 0.31 | 0.19 | 162952996 | 0.65 | 0.20 | 0.56 | 0.37 |
| 162874247 | 0.29 | 0.33 | 0.62 | 0.12 | 162953557 | 0.87 | 0.07 | 0.38 | 0.11 |
| 162874358 | 0.87 | 0.51 | 0.54 | 0.20 | 162955224 | 0.78 | 0.35 | 0.52 | 0.16 |
| 162876410 | 0.83 | 0.97 | 0.87 | 0.28 | 162956849 | 0.72 | 0.46 | 0.84 | 0.56 |
| 162876411 | 0.83 | 0.97 | 0.87 | 0.28 | 162957338 | 0.40 | 0.43 | 0.03 | 0.35 |
| 162878377 | 0.25 | 0.56 | 0.38 | 0.60 | 162963098 | 0.24 | 0.41 | 0.16 | 0.39 |
| 162878378 | 0.25 | 0.56 | 0.38 | 0.60 | 162963358 | 0.93 | 0.20 | 0.23 | 0.08 |
| 162880289 | 0.80 | 0.04 | 0.61 | 0.42 | 162963628 | 0.73 | 0.25 | 0.87 | 0.32 |
| 162884805 | 0.63 | 0.27 | 0.53 | 0.34 | 162964435 | 0.85 | 0.62 | 0.95 | 0.16 |
| 162885142 | 0.13 | 0.07 | 0.14 | 0.23 | 162965363 | 0.10 | 0.04 | 0.09 | 0.35 |
| 162885807 | 0.70 | 0.22 | 0.13 | 0.30 | 162968547 | 0.72 | 0.53 | 0.17 | 0.45 |
| 162888038 | 0.15 | 0.11 | 0.26 | 0.20 | 162970859 | 0.80 | 0.16 | 0.89 | 0.34 |
| 162890111 | 0.80 | 0.97 | 0.77 | 0.39 | 162971118 | 0.22 | 0.09 | 0.24 | 0.14 |
| 162893251 | 0.71 | 0.15 | 0.90 | 0.35 | 162971309 | 0.93 | 0.77 | 0.26 | 0.10 |
| 162895380 | 0.05 | 0.08 | 0.20 | 0.36 | 162971728 | 0.65 | 0.05 | 0.02 | 0.48 |
| 162895538 | 0.68 | 0.56 | 0.18 | 0.47 | 162973005 | 0.76 | 0.14 | 0.78 | 0.43 |
| 162897369 | 0.62 | 0.33 | 0.85 | 0.54 | 162973509 | 0.53 | 0.33 | 0.86 | 0.46 |
| 162898546 | 0.07 | 0.38 | 0.54 | 0.38 | 162973510 | 0.53 | 0.33 | 0.86 | 0.46 |
| 162899427 | 0.26 | 0.70 | 0.18 | 0.33 | 162974828 | 0.94 | 0.99 | 0.92 | 0.84 |
| 162900804 | 0.81 | 0.33 | 0.64 | 0.56 | 162975776 | 0.97 | 0.40 | 0.68 | 0.23 |
| 162902728 | 0.61 | 0.32 | 0.86 | 0.44 | 162975883 | 0.72 | 0.33 | 0.91 | 0.23 |
| 162903500 | 0.35 | 0.10 | 0.20 | 0.33 | 162976009 | 0.79 | 0.23 | 0.82 | 0.22 |
| 162905858 | 0.79 | 0.33 | 0.63 | 0.31 | 162981932 | 0.05 | 0.18 | 0.26 | 0.25 |
| 162910735 | 0.94 | 0.52 | 0.39 | 0.47 | 162983832 | 0.79 | 0.65 | 0.17 | 0.02 |
| 162910955 | 0.83 | 0.40 | 0.80 | 0.29 | 162984585 | 0.14 | 0.18 | 0.38 | 0.10 |
| 162912527 | 0.46 | 0.56 | 0.35 | 0.47 | 162993729 | 0.69 | 0.98 | 0.80 | 0.32 |
| 162913077 | 0.76 | 0.55 | 0.27 | 0.03 | 162995221 | 0.21 | 0.14 | 0.34 | 0.14 |
| 162995965 | 0.69 | 0.25 | 0.50 | 0.30 | 163082706 | 0.52 | 0.27 | 0.47 | 0.35 |
| 162999362 | 0.84 | 0.74 | 0.55 | 0.60 | 163084105 | 0.76 | 0.62 | 0.83 | 0.58 |
| 163003104 | 0.02 | 0.31 | 0.04 | 0.36 | 163085934 | 0.59 | 0.22 | 0.41 | 0.25 |
| 163003554 | 0.41 | 0.22 | 0.31 | 0.13 | 163086875 | 0.55 | 0.22 | 0.76 | 0.37 |
| 163003555 | 0.41 | 0.22 | 0.31 | 0.13 | 163105183 | 0.60 | 0.45 | 0.24 | 0.44 |
| 163004540 | 0.15 | 0.05 | 0.10 | 0.26 | 163105607 | 0.88 | 0.33 | 0.29 | 0.30 |
| 163004763 | 0.03 | 0.08 | 0.02 | 0.28 | 163115075 | 0.70 | 0.46 | 0.71 | 0.26 |
| 163005368 | 0.59 | 0.38 | 0.28 | 0.35 | 163185371 | 0.68 | 0.26 | 0.89 | 0.19 |
| 163010578 | 0.74 | 0.53 | 0.68 | 0.75 | 163185419 | 0.03 | 0.11 | 0.23 | 0.15 |
| 163011590 | 0.63 | 0.59 | 0.74 | 0.63 | 163185622 | 0.75 | 0.53 | 0.21 | 0.37 |
| 163012984 | 0.28 | 0.35 | 0.44 | 0.43 | 163185892 | 0.20 | 0.15 | 0.20 | 0.26 |
| 163014941 | 0.04 | 0.39 | 0.05 | 0.06 | 163186393 | 0.26 | 0.07 | 0.10 | 0.34 |
| 163015292 | 0.91 | 0.39 | 0.18 | 0.01 | 163186826 | 0.37 | 0.19 | 0.32 | 0.27 |
| 163016882 | 0.86 | 0.38 | 0.34 | 0.54 | 163186897 | 0.73 | 0.53 | 0.26 | 0.34 |
| 163017755 | 0.32 | 0.48 | 0.39 | 0.48 | 163187092 | 0.47 | 0.69 | 0.44 | 0.78 |
| 163018012 | 0.88 | 0.23 | 0.84 | 0.09 | 163187632 | 0.47 | 0.32 | 0.12 | 0.38 |
| **CID** | **DILI** | **Ames** | **AOT** | **Car** | **CID** | **DILI** | **Ames** | **AOT** | **Car** |
| 163018356 | 0.91 | 0.23 | 0.20 | 0.78 | 163188089 | 0.87 | 0.56 | 0.82 | 0.48 |
| 163021866 | 0.76 | 0.18 | 0.23 | 0.25 | 163188272 | 0.44 | 0.32 | 0.10 | 0.42 |
| 163025008 | 0.45 | 0.80 | 0.42 | 0.07 | 163188791 | 0.81 | 0.28 | 0.88 | 0.22 |
| 163026334 | 0.91 | 0.30 | 0.20 | 0.29 | 163189088 | 0.84 | 0.56 | 0.22 | 0.42 |
| 163026908 | 0.65 | 0.50 | 0.69 | 0.72 | 163190772 | 0.81 | 0.54 | 0.21 | 0.04 |
| 163028748 | 0.56 | 0.34 | 0.13 | 0.32 | 163192272 | 0.62 | 0.53 | 0.42 | 0.35 |
| 163029829 | 0.95 | 0.65 | 0.55 | 0.32 | 163193473 | 0.94 | 0.25 | 0.26 | 0.29 |
| 163030723 | 0.74 | 0.21 | 0.85 | 0.41 | 163193561 | 0.61 | 0.32 | 0.86 | 0.44 |
| 163030830 | 0.45 | 0.42 | 0.89 | 0.35 | 163193927 | 0.91 | 0.89 | 0.97 | 0.53 |
| 163033851 | 0.79 | 0.93 | 0.71 | 0.37 | 163194738 | 0.20 | 0.42 | 0.17 | 0.07 |
| 163036215 | 0.65 | 0.21 | 0.76 | 0.33 | 163195533 | 0.91 | 0.42 | 0.81 | 0.46 |
| 163040884 | 0.88 | 0.97 | 0.88 | 0.14 | 163063328 | 0.74 | 0.39 | 0.80 | 0.54 |
| 163042119 | 0.88 | 0.60 | 0.65 | 0.59 | 163067369 | 0.94 | 0.45 | 0.25 | 0.39 |
| 163042754 | 0.16 | 0.08 | 0.15 | 0.17 | 163069224 | 0.94 | 0.25 | 0.26 | 0.29 |
| 163045194 | 0.27 | 0.48 | 0.44 | 0.65 | 163070745 | 0.88 | 0.30 | 0.75 | 0.47 |
| 163046290 | 0.74 | 0.19 | 0.86 | 0.12 | 163072561 | 0.60 | 0.38 | 0.42 | 0.57 |
| 163046350 | 0.06 | 0.10 | 0.27 | 0.35 | 163075353 | 0.09 | 0.15 | 0.23 | 0.38 |
| 163047849 | 0.77 | 0.63 | 0.91 | 0.11 | 163075826 | 0.95 | 0.48 | 0.60 | 0.19 |
| 163049962 | 0.77 | 0.28 | 0.83 | 0.51 | 163075861 | 0.62 | 0.37 | 0.89 | 0.43 |
| 163057991 | 0.29 | 0.05 | 0.24 | 0.12 | 163078747 | 0.05 | 0.22 | 0.29 | 0.22 |
| 163059685 | 0.53 | 0.39 | 0.31 | 0.73 | 163080755 | 0.74 | 0.36 | 0.65 | 0.35 |
| 163063327 | 0.74 | 0.39 | 0.80 | 0.54 | 163082705 | 0.52 | 0.27 | 0.47 | 0.35 |

*DILI – Drug Induced Liver Injury, Ames - Ames Mutagenesis, AOT – Acute Oral Toxicity, and Car - Carcinogenicity
